# Postoperative sore throat: a systematic review*

**DOI:** 10.1111/anae.70048

**Published:** 2025-10-28

**Authors:** Zachary J. Moulder, Jason Mann, Paul Bramley, Jana Heinz, Matthew D. Wiles

**Affiliations:** ^1^ Department of Academic Anaesthesia Sheffield Teaching Hospitals NHS Foundation Trust Sheffield UK; ^2^ University of Sheffield Sheffield UK; ^3^ Centre for Applied Health and Social Care Research (CARe) Sheffield Hallam University Sheffield UK

**Keywords:** anaesthesia, adverse effects, anaesthesia, general, postoperative sore throat, supraglottic airway device, tracheal intubation

## Abstract

**Introduction:**

Postoperative sore throat is a common complaint with an incidence of up to 62%. While anaesthetists often perceive this as a minor and self‐limiting complication, postoperative sore throat is one of the leading causes of postoperative anaesthesia‐related discomfort. Preventative strategies for postoperative sore throat have been studied extensively, but well‐evidenced recommendations are lacking.

**Methods:**

We performed a systematic review to summarise interventions which may prevent postoperative sore throat. Two independent reviewers assessed studies against inclusion criteria and completed a Cochrane Risk of Bias 2 assessment for randomised controlled trials. The results were synthesised narratively due to extensive methodological heterogeneity (populations, interventions and outcomes).

**Results:**

We identified 1883 studies, of which 162 met the inclusion criteria (enrolling 21,199 patients). The pooled incidence of postoperative sore throat at 1 h was 32.4% (95%CI 26.9–38.5%) in 43 studies involving tracheal intubation and 29.4% (95%CI 20.5–40.2%) in 18 studies that used a supraglottic airway device. At 24 h, the pooled incidence of postoperative sore throat was 16.4% (95%CI 13.6–19.8%) in 93 studies involving tracheal intubation and 9.9% (95%CI 6.7–14.4%) in 23 studies that used supraglottic airway devices. Interventions with evidence of benefit included maintaining cuff pressure ≤ 60 cmH_2_O for supraglottic airway devices and ≤ 30 cmH_2_O for tracheal tubes. For tracheal tubes only, other interventions with benefit included use of topical ketamine; intravenous or topical steroids; and topical non‐steroidal anti‐inflammatory drugs.

**Discussion:**

Despite the high incidence of postoperative sore throat, the current literature lacks high‐quality randomised controlled trials on treatments that prevent a complication that is of importance to patients and their recovery. New research will only add value to this area if studies adequately control for confounders.

## Plain Language Summary

Getting a sore throat after surgery is very common, happening in more than half of patients. Doctors often think of it as a small problem, but for patients, it can be very uncomfortable. Many studies have looked at ways to a stop sore throat after surgery, but we still don't have clear advice that works for everyone. We looked at many studies to see what treatments might help prevent a sore throat after surgery. Two researchers checked the studies carefully to make sure they were of good quality. Because the studies were all a bit different, we described the results using words instead of combining them into one big calculation. We found 1883 studies. Out of these, 162 were good enough to include; more than 21,000 patients took part in these studies. After 1 hour, about 32 out of 100 patients who had a breathing tube, and about 29 out of 100 who had a special airway mask, got a sore throat. After 24 hours, about 16 out of 100 with a breathing tube and about 10 out of 100 with a special airway mask still had a sore throat. Treatments that seemed to help reduce a sore throat included keeping the pressure in the airway tube or mask low. For breathing tubes, medicines such as ketamine, steroids or anti‐inflammatory drugs (given as sprays, creams or through a drip) also helped. Sore throat after surgery happens often, but we still don't have good research on how to stop it. Future studies need to be better planned and look at patient comfort while taking into account different factors.

## Introduction

Postoperative sore throat (POST) is a common complaint with an incidence of up to 62% [1, 2]. Whilst anaesthetists often perceive this as a minor and self‐limiting complication, POST is one of the leading causes of postoperative anaesthesia‐related discomfort [3] and may be a cause of reduced patient satisfaction with anaesthetic care [4]. Some patients still complain of POST up to 96 h after surgery [5] and this may prevent them from resuming normal activities, including eating and drinking [6, 7], which is a key component of modern enhanced peri‐operative care pathways [8].

Risk factors for POST include: female sex; older age; size and cuff pressure of airway device; duration of airway device placement; number attempts to insert an airway device; and smoking history [1, 2]. There have been multiple randomised controlled trials investigating preventative treatments for POST, but the cuff pressure of tracheal tubes or supraglottic airway devices (SAD), a known risk factor for POST, is often not controlled for.

We performed a systematic review to investigate interventions that prevent POST in randomised controlled trials, which controlled for major confounders, including oral surgery; insertion of nasogastric tubes or pharyngeal packs; and airway device cuff pressure.

## Methods

This study was conducted according to PRISMA and other methodological guidelines [9, 10].

We searched MEDLINE and Embase from inception on 6 January 2025 (search strategy available in online Supporting Information Appendix S1). We also reviewed references for any relevant omitted trials before importing them into an online systematic review tool (Covidence, Melbourne, Australia). We included published, peer‐reviewed randomised controlled trials with a primary outcome of incidence of POST, performed in adults (≥ 18 y) or children (< 18 y) in any language if translation was available. We did not study trials with any of the following criteria: insertion of nasogastric tubes or throat packs; surgeries involving the larynx, pharynx or oral cavity; and absence of airway device cuff pressure control.

Our primary outcome was the incidence of sore throat in the first 24–48 h after surgery. Secondary outcomes included incidence of POST at any other time point considered by the study authors; pain scores for POST (measured using whatever tool the study authors chose, e.g. visual analogue scale or Likert rating scale); incidence of postoperative nausea and vomiting; and any adverse effects associated with interventions. After full text screening, we limited our analysis to outcomes pertaining specifically to POST and did not assess postoperative nausea and vomiting or adverse effects; this was due to the large number of studies identified, the review team size and the time available for this study.

Each study was assessed by two reviewers (ZM, JH, JM or MW) who performed abstract and full‐text screening independently. Disagreements were resolved by discussion with a third reviewer until a consensus was reached. Data were then extracted into an Excel spreadsheet (Microsoft Corporation, Redmond, WA, USA) using a template (online Supporting Information Table S1). For imputation of means and standard deviations, we used Meta‐Analysis Accelerator [11]. When data extraction was necessary from graphs, we used https://automeris.io. Measurement of POST in the post‐anaesthesia care unit (PACU) was treated as a 1‐h time point unless a specific hour after surgery was stated. Measurement at an epoch of <1 h was rounded appropriately to the nearest hour. Incomplete or missing data were recorded as not reported, and therefore, no assumptions were made. Risk of bias assessments were performed by two independent reviewers using the Cochrane Risk of Bias‐2 tool [12].

We decided whether to perform a meta‐analysis based on whether studies were adequately similar in terms of population, intervention and outcome. This decision was made using clinical expertise. For studies that used a tracheal tube or SAD, we used a random effects model to calculate a pooled estimate of the incidence of POST at 1 h and 24 h, using a mixed‐effects logistic regression and intervals calculated using the normal distribution. Random‐effects analysis was used due to clear residual clinical heterogeneity in included studies. Statistical analysis was undertaken in R 4.4.1 (R Foundation, Vienna, Austria), using the metafor package (version 4.8.0, https://metafor‐project.org/doku.php/metafor). The weight of each study was visualised via forest plots for each time point calculated.

## Results

**Figure 1:**
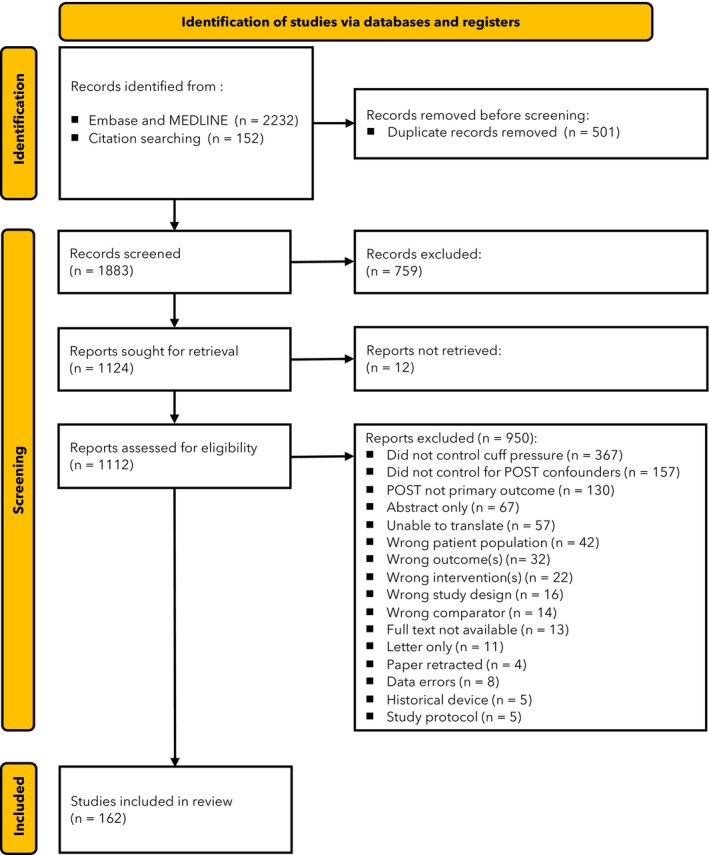
Study flow diagram. POST, postoperative sore throat.

We identified 1883 trials; 162 studies enrolling 21,199 patients met the inclusion criteria (Fig. 1). Full details of included studies are available in online Supporting Information Table S2. Due to extensive methodological heterogeneity in patient populations, interventions and outcome measurement, meta‐analysis of effect was not appropriate; we therefore reported the review findings narratively.

Overall, the randomised controlled trials identified and included were of mixed quality and showed a high degree of heterogeneity across interventions and outcomes. The majority involved small patient populations, with variable control of confounders and mixed estimations of effect. Studies varied in country of origin and surgical specialty, but few were multicentre or multinational.

Many studies were at high risk of bias (68/162, 42.0%) (online Supporting Information Figure S1) and often reported contradictory results. Only 5/162 (3.1%) studies were considered at low risk of bias [13, 14, 15, 16, 17]. Due to the significant number of studies judged to be at some or high risk of bias, and high levels of conflict between studies on evidence of effect, we were unable to rate the evidence using GRADE recommendations. We therefore summarised the main interventions for the reduction of POST in tabular form without a strength of recommendation (Table 1).

**Table 1:** Potential interventions to reduce the incidence of postoperative sore throat.

Tracheal intubation (single‐lumen tube)	Cuff pressure maintenance ≤ 30 cmH_2_O [18, 19, 20, 21, 22, 23] Intravenous/topical corticosteroids^†^ [14, 24, 25, 26, 27, 28, 29, 30, 31, 32, 33, 34, 35, 36, 37, 38, 39, 40] Nebulised/topical ketamine [27, 28, 34, 41, 42, 43, 44, 45, 46, 47, 48, 49, 50, 51, 52] Topical NSAIDs [42, 45, 53, 54, 55, 56, 57, 58, 59, 60, 61] Smaller tracheal tube internal diameter: 7.0 mm (male); 6.0 mm female [5] Use of a supraglottic airway device as an alternative* [62, 63, 64]
Tracheal intubation (double‐lumen tube)	Pre‐warming tracheal tube to ≥ 40°C^†^ [16] Topical NSAIDs [65, 66]
Supraglottic airway devices	Cuff pressure maintenance ≤ 60 cmH_2_O [67, 68, 69]

NSAIDs, nonsteroidal anti‐inflammatory drugs.

* Where clinically appropriate.

^†^ Denotes studies judged to be at low risk of bias.

The pooled incidence of POST at 1 h was 32.4% (95%CI 26.9–38.5%) in 43 studies involving tracheal intubation and 29.4% (95%CI 20.5–40.2%) in 18 studies that used a SAD. At 24 h, the pooled incidence of POST was 16.4% (95%CI 13.6–19.8%) in 93 studies involving a tracheal tube and 9.9% (95%CI 6.7–14.4%) in 23 studies that used a SAD. Forest plots are available in online Supporting Information Figure S2.

### Pharmacological interventions – tracheal tube

#### Lidocaine

The main applications for lidocaine have been topical (lubricating gel on tracheal tube cuff or aerosolised) or as a medium for tracheal cuff inflation. Lidocaine gel applied to the tracheal tube and cuff was associated with an increased incidence of POST [70, 71, 72, 73] and was inferior to a pre‐operative gargle with ketamine 40 mg [74]. When compared with betamethasone gel, the incidence of POST with lidocaine gel has been shown to be similar [72] or worse [24].

Intracuff lidocaine has two proposed mechanisms of action: diffusion of lidocaine through the tracheal tube cuff providing direct local anaesthesia to the tracheal mucosa; and reduction in intra‐operative tracheal cuff pressures, especially in the presence of nitrous oxide. Eleven studies studied intracuff lidocaine: 10 used lidocaine alkalinised with sodium bicarbonate to increase lidocaine ionisation and thereby enhance diffusion (typically 2% lidocaine with 7.5% sodium bicarbonate in a ratio 19:1) [75, 76, 77, 78, 79, 80, 81, 82, 83, 84]; one used only 10% lidocaine [85]. All studies included nitrous oxide in the anaesthetic gas mixture. Duration of surgeries ranged from 80 min to > 220 min; this is an important consideration as the diffusion of lidocaine out of the cuff is gradual, with one study showing peak mean plasma concentration at 90 min [85]. Tracheal cuff pressures were lower when inflated with fluid (either lidocaine or saline) compared with air [76, 78, 79, 81, 82]. This contributed to a lower incidence of POST at 24 h in studies comparing intracuff air with lidocaine [75, 76, 78, 79, 82], except the study by Prajapati et al., who showed no difference [81]. The benefits of intracuff lidocaine compared with saline were less clear; although cuff pressures with lidocaine were lower or similar compared with saline, the incidence of POST at 24 h with intracuff lidocaine was reduced in three studies [83, 84, 85] and unchanged in four [77, 78, 80, 82].

Three studies assessed intracuff alkalinised lidocaine in children. In patients aged 3–13 y undergoing surgery predicted to be > 60 min, the use of intracuff 0.5% and 1% alkalinised lidocaine compared with air appeared to reduce the risk of POST at 8 h, but not in the PACU, in one of five studies judged to be at low risk of bias [13]. There was no evidence of an effect of intracuff lidocaine compared with saline, despite nitrous oxide use and intra‐operative cuff pressure being maintained <20 cmH_2_O. Two studies used 2% lidocaine in children with an average anaesthetic time around 150 min; one showed a significant reduction in POST in PACU and at 24 h [86], whilst the other showed no reduction but may have been underpowered [87].

There are conflicting reports regarding the effect of an intravenous bolus of lidocaine 1.5 mg.kg^‐1^ with dexamethasone 8 mg before tracheal intubation on the incidence of POST, with one study judged to be at some risk of bias suggesting benefit [88] but another study judged to be at low risk of bias finding that the benefit was due to the dexamethasone alone [14]. Although the same dose of intravenous lidocaine was found to be superior to placebo in terms of the incidence and severity of POST in the first 24 h, this was inferior to direct topicalisation of the larynx [89].

#### Steroids

Several different steroid formulations were studied via a range of routes, including nebulised; intravenous; topical; and gargle.

Beclomethasone has been used as a topical spray and as a gel applied directly to the tracheal tube. The use of 50% beclomethasone spray reduced the incidence of POST in the first 24 h compared with control and was superior to 10% lidocaine spray [25]. Similarly, when compared with lidocaine gel, 0.05% beclomethasone gel applied to the tracheal tube was more effective in reducing the incidence of POST in the first 24 h [24, 26]. However, one randomised controlled trial found the application of distilled water was more effective in reducing the incidence of early POST at 1–6 h than lidocaine or beclomethasone gel, but this difference was no longer significant at 24 h [72]. Beclomethasone gel has also been shown to be as effective as ketamine gargle [27, 28] and intravenous dexamethasone [28] in reducing the incidence of POST. Similarly, lubrication of tracheal tubes with 0.1% triamcinolone paste reduced the incidence and severity of POST compared with a control group who received chlorhexidine gel as a lubricant [29].

Inhaled budesonide was administered in three studies and was associated with a reduction in the incidence of POST in the first 24 h [30, 31, 32].

Dexamethasone is the steroid most studied to prevent POST. Most studies investigating intravenous dexamethasone used weight‐adjusted (0.1–0.2 mg.kg^‐1^) or single bolus (8–10 mg) doses. All doses reduced the incidence and severity of POST within the first 24 h [14, 33, 34, 35, 36] for studies using single‐lumen tracheal tubes. Only one study investigated double‐lumen tubes and showed that dexamethasone 0.2 mg.kg^‐1^ was superior to 0.1 mg.kg^‐1^ in reducing the incidence of POST [37]. The prophylactic administration of intravenous dexamethasone (i.e. before tracheal intubation vs. intra‐operatively) may reduce the incidence of early (≤ 6 h) but not late (24 h) POST [38, 39]. When combined with intravenous dexamethasone, ketamine gargle reduces the incidence of POST further [34, 36]. There are conflicting data regarding the additional benefit of co‐administration with intravenous lidocaine 1.5 mg.kg^‐1^ [14, 88].

When compared with nebulised magnesium, nebulised dexamethasone 8 mg significantly reduced the incidence and severity of early POST (6–12 h), but this was not sustained at 24 h [40, 90].

The use of 0.05% dexamethasone gargle has been reported as having similar efficacy to intravenous dexamethasone 0.1 mg.kg^‐1^ to reduce the incidence of POST [91]. The same dose has also been shown to reduce the severity of POST in a single trial, although pain scores were generally low [92].

#### Ketamine

Ketamine has been investigated widely to prevent POST, primarily as a gargle or via a nebuliser before tracheal intubation. Intravenous low‐dose ketamine was not effective [93], whereas ketamine 40–50 mg gargle has been shown to reduce the incidence of POST compared with placebo [27, 28, 34, 41, 42, 43, 44, 45, 46, 47, 94]. In most studies, the severity of POST was also reduced. Only one small randomised controlled trial found that a ketamine gargle did not reduce the incidence of POST relative to a control group (normal saline gargle) [44]. Larger doses of ketamine have also been investigated: 0.5 mg.kg^‐1^ gargle reduced the incidence of POST compared with control, although 50 mg and 100 mg had similar incidences. Co‐administering intravenous dexamethasone 0.2 mg.kg^‐1^ with ketamine gargle may offer an additional benefit to reduce the incidence of POST [34, 36]. Ketamine gargle had a similar effect to betamethasone gel applied to the tracheal tube cuff to reduce the incidence of POST [27, 28]. However, comparing a ketamine gargle with a 0.5 mg.kg^‐1^ magnesium gargle has shown conflicting results [48, 94].

Nebulised ketamine 50 mg has been compared with nebulised magnesium 250 mg; both drugs were shown to be superior to saline in reducing the incidence of POST at 4–24 h [49, 50]. Direct comparison of the same doses of nebulised magnesium and ketamine suggests ketamine is more effective at reducing the incidence of POST [50, 51, 52]. Nebulised ketamine 50 mg reduced the early incidence of POST to a greater degree than nebulised lidocaine 40 mg, but this difference was not sustained at 12–24 h [95]. A single study suggested that the studied dose of nebulised ketamine may be too low, with doses ≥ 1.0 mg.kg^‐1^ being more effective in reducing the incidence and severity of POST [96].

#### Magnesium

Several studies investigated the effect of pre‐operative nebulisation of magnesium on the incidence of POST. Compared with placebo, nebulised magnesium 225 mg reduced the incidence and severity of POST at 1 h, 6 h and 24 h in patients undergoing lumbar spine surgery who also received dexamethasone 8 mg intravenously [97]. However, a study that used the same dose of magnesium, but did not administer dexamethasone, showed that the incidence of POST was reduced at 24 h but not at ≤ 4 h [98].

When nebulised magnesium was compared directly with nebulised dexamethasone 8 mg, there were conflicting results: 250 mg was shown to be superior [90], but 1000 mg was inferior [40] to dexamethasone for reducing the incidence of POST. Nebulised budesonide 250 μg reduced the incidence of POST at 48 h to a similar degree to nebulised magnesium 250 mg [31]. Four studies compared nebulised magnesium 250 mg to nebulised ketamine 25–50 mg; both appeared to reduce POST, but results were conflicting, with one favouring magnesium [49] and three favouring ketamine [50, 51, 52]. Compared with nebulised lidocaine 100 mg, nebulised magnesium 250 mg reduced the incidence and severity of POST for up to 24 h [99].

Magnesium has also been administered by gargle, lozenges and intravenously. Magnesium gargle 30 mg.kg^‐1^ reduced the incidence and severity of POST at 2 h, 4 h and 24 h but not immediately after arrival in the PACU [94]. When compared with ketamine 0.5 mg.kg^‐1^ gargle, results were conflicted, but both drugs were more effective than placebo [48, 94]. Intravenous magnesium (30 mg.kg^‐1^ bolus then continuous infusion of 10 mg.kg^‐1^.h^‐1^) was similar to 8 mg intravenous bolus of dexamethasone in reducing the incidence and severity of POST for up to 48 h [100]. Magnesium lozenges reduced the incidence and severity of early POST at ≤ 4 h and ≤ 2 h, respectively, but were similar to placebo at 24 h [101].

#### Nonsteroidal anti‐inflammatory drugs

Nonsteroidal anti‐inflammatory drugs (NSAIDs) to prevent POST have been investigated widely. Benzydamine has been used topically as a gargle or an oropharyngeal spray, and reduces the incidence and severity of POST when both single‐ and double‐lumen tracheal tubes are used [42, 45, 53, 54, 55, 56, 57, 58, 59]. Benzydamine spray applied to the vocal cords and trachea was found to have no effect on POST in a paediatric population in one of five studies judged to be at low risk of bias [15]. Other topical NSAIDs, such as flurbiprofen pharyngeal spray [56, 60] and diclofenac gel on the tracheal tube cuff, have shown similar efficacy [61]. The benefits of NSAIDs in terms of attenuating POST may relate to a systemic effect, as transdermal NSAIDs [102, 103] and lozenges containing flurbiprofen [104] have also shown benefits.

Liquorice is derived from the root of *Glycyrrhiza glabra* and is believed to have an anti‐inflammatory effect. When diluted with water and used as a gargle pre‐operatively, liquorice reduced the early (< 24 h) incidence and severity of POST after tracheal intubation with single‐ [105] and double‐lumen tracheal tubes [65, 66]. One study compared liquorice and ketamine gargles with placebo and showed liquorice to have a similar efficacy to ketamine in terms of reductions in incidence and severity of POST [66].

Aescin is an extract of horse chestnut (*Aesculus hippocastanum*) that is taken orally and may have anti‐inflammatory properties. However, a pre‐operative dose of aescin had no effect on the incidence or severity of POST in the first 24 h [106]. Azulene, a chamomile extract, also has anti‐inflammatory effects, and when administered as a pre‐operative gargle, reduced the incidence and severity of POST for the first 24 h [107].

#### Maintenance anaesthetic and neuromuscular blocking drugs

Reports are conflicted regarding the efficacy of neuromuscular blocking drugs in reducing the incidence of POST. One study using rocuronium vs. placebo showed a reduction in POST at 2 h and 24 h (42% vs. 57% and 26% vs. 38%, respectively) [108]. A continuous infusion of neuromuscular blocking drugs (with train of four target ≤ 1) reduced the incidence of POST compared with intermittent boluses [109]. However, a study that compared cisatracurium with placebo in addition to a moderate dose of remifentanil (2 μg.kg^‐1^) showed a similar incidence of POST at 24 h and 48 h [110]. High‐dose remifentanil may be associated with hyperalgesia, with one randomised controlled trial showing an increased incidence of POST in patients who received a high‐dose intra‐operative infusion (starting at 0.25 μg.kg^‐1^.min^‐1^ and then titrated to blood pressure and processed electroencephalogram) compared with a fixed, low‐dose regimen (0.05 μg.kg^‐1^.min^‐1^) [111]. Similarly, when compared with remifentanil, the use of dexmedetomidine as an intra‐operative analgesic adjuvant reduced the incidence of POST within the first 24 h [112]. Maintenance of anaesthesia with sevoflurane vs. TIVA did not impact the incidence of POST [113].

#### Others

Lubricating the tracheal tube cuff with drugs other than local anaesthetics is of variable efficacy: diclofenac gel reduced the incidence of POST [61], while water and chamomile extract had no effect [114, 115]. Dexpanthenol is an alcohol derivative of pantothenic acid, a component of the B complex vitamins, and is an important mediator of normal epithelial function; it is used widely to manage dermatological conditions. When administered pre‐operatively as a pastille, dexpanthenol reduced the incidence of POST for up to 24 h compared with placebo and benzydamine spray [57]. Compared with placebo and a sham intervention, acupuncture at the Korean hand acupuncture point reduced the incidence, but not the severity, of POST at 24 h [116].

### Non‐pharmacological interventions – tracheal tubes

Thirty‐one studies investigated non‐pharmacological methods to prevent POST: nine examined cuff pressure management methods; seven focused on airway insertion techniques; seven focused on double‐lumen tubes; and eight compared type of tracheal tube.

#### Insertion techniques

Compared with a bevel orientated to the patient's left, insertion of a Shiley™ Lo‐Contour flexible reinforced tracheal tube (Covidien, Tullamore, Ireland) with the bevel orientated dorsally was associated with reduced POST severity at 24 h [117]. Pre‐warming tracheal tubes to 40°C reduced the incidence of POST at 1 h (48/91 vs. 33/94, p = 0.02) but not at 24 h (37/91 vs. 36/94, p = 0.77) [118]. Jaw thrust during tracheal intubation also showed a significant reduction in the incidence and severity of POST in the first 24 h [119].

Compared with direct laryngoscopy, videolaryngoscopy using a GlideScope (Verathon, Bothell, WA, USA) significantly reduced the incidence and severity of early (0 h and 6 h) but not late (24 h) POST [120]. The use of a rigid stylet (Hansraj Nayyar Medical, Mumbai, India) in conjunction with direct laryngoscopy reduced the incidence and severity of early (≤ 4 h) but not late (24 h) POST. The use of a bougie (Eschmann multiple‐use introducer; Smith's Medical International, Hythe, UK) had no effect on the incidence of POST [121]. A similar result was obtained when a stylet was used with a McGrath® MAC videolaryngoscope (Medtronic, Minneapolis, MN, USA) [122].

#### Tracheal tube factors

The use of tracheal tubes with a tapered cuff (TaperGuard™; Medtronic) has been shown to reduce the incidence and severity of POST [123, 124]. Tapered tracheal tube cuffs (cuff pressure controlled at 25 cmH_2_O) significantly reduced the incidence and severity at 6 h, but not at 1 h or 24 h [123]. In a similar study, tapered tracheal tube cuffs significantly reduced the incidence of POST at all time points up to 24 h [124]. Ozhan‐Akdemir et al. showed that, after having initially been set to 25 cmH_2_O, cylindrical tracheal tube cuff pressure increased more compared with tapered tracheal tubes (mean (SD) 37.2 (3.65) cmH_2_O vs. 28.6 (2.79) cmH_2_O, respectively, p < 0.05), which may account for some of this effect [124].

Several studies investigated the effect of reducing contact between the tracheal tube and the tracheal mucosa [5, 62, 63, 64]. Incidence of POST was significantly reduced with smaller tracheal tube size use in women (internal diameter 6.0 vs. 7.0 mm, risk ratio 0.56, 95%CI 0.35‐0.90, p = 0.02) but not men (7.0 vs 8.0 mm, risk ratio 0.74, 95%CI 0.43–1.26, p = 0.27) [5]. By reducing the requirement for oropharyngeal suction, the use of a Suction Above Cuff Endotracheal Tube (SACETT™, Smiths Medical International) reduced the incidence of POST in the PACU compared with a standard tracheal tube (2/66 vs. 9/66, p = 0.027) [125]. Incidence of POST is significantly reduced with the use of an SAD compared with a tracheal tube. A third‐generation SAD, the Baska Mask (Baska, Strathfield, Australia), reduced the incidence of POST within the first 24 h (0/60 vs. 12/60, p = 0.024) [62]. Similar benefits were attributed to the use of the LMA® Classic™ (Teleflex Medical, Athlone, Ireland) [63] and LMA Supreme™ (Teleflex Medical) [64]; both these studies only studied women and the comparator was a tracheal tube of internal diameter 6.5–7.0 mm.

#### Tracheal tube cuff pressure monitoring

Continuous tracheal tube cuff pressure monitoring is reported to reduce POST [18, 19, 20]. Automated cuff pressure maintenance at 25–30 cmH_2_O in a low‐pressure, high‐volume tracheal tube cuff significantly reduced the incidence of POST at 24 h when compared with hourly adjustment or unmonitored cuff pressure (13.2% vs. 29.7% vs. 52.8%, p = 0.001) [18]. Manual adjustment of cuff pressure to 25–30 cmH_2_O significantly reduced the incidence of POST in the first 24 h (1/30 vs. 8/30, p = 0.013) compared with initial pilot balloon palpation and no further adjustment of cuff pressure [19]. One study examined the recommended range of tracheal cuff pressure (20–30 cmH_2_O) when adjusted every 10 min [20]. There was no significant difference in the 48‐h incidence of POST with tracheal tube cuff pressures of 20 cmH_2_O, 25 cmH_2_O and 30 cmH_2_O. However, a cuff pressure of 15 cmH_2_O significantly reduced the incidence of POST at 24 h compared with 30 cmH_2_O (4/25 vs. 15/25, p < 0.05), despite a longer duration of tracheal intubation (11.8 min difference, p < 0.05) [20].

Multiple studies have compared minimally manipulated tracheal tube cuff pressure with common inflation techniques [21, 22, 23, 126]. In a study that used nitrous oxide‐based anaesthesia, the incidence and severity of POST were similar with cuff inflation to a minimal seal pressure (lowest volume of air to prevent a leak with an intra‐airway pressure of 20 cmH_2_O) compared with a set cuff inflation pressure of 25 cmH_2_O [126]. Similarly, no difference in the incidence of POST was noted between a preset cuff pressure of 25 cmH_2_O and a sealing pressure of 20 cmH_2_O in oxygen/air‐based anaesthesia [21]. However, both groups were noted to have a significantly lower incidence of POST compared with pilot balloon palpation, which averaged a cuff pressure of 48 cmH_2_O.

Palpation of the tracheal tube pilot balloon is a very poor way to assess cuff pressure. Measurement and adjustment of a cuff inflated using pilot balloon palpation reduced the mean (SD) pressure from 58 cmH_2_O to 27 (4) cmH_2_O and was associated with a reduced incidence of POST [22]. A similarly reduced incidence of POST at 24 h was seen when cuff pressure was adjusted using a manometer after initial inflation using pilot balloon palpation [23]. Of note, only 43% of patients in this study had an initial cuff pressure > 30 cmH_2_O. Both studies did not measure cuff pressure continuously intra‐operatively, and one had uneven group sizes [22, 23]. In all these studies, the duration of tracheal intubation was 1.5–4.5 h, which may have further increased the incidence of POST [21, 22, 23, 126].

Other tracheal tube cuff pressure management methods have also reduced POST. In patients having cardiac surgery (14 h average tracheal intubation time), cuff inflation guided by volume‐time curve analysis reduced the incidence and severity of POST at 24 h (90/222 vs. 43/228, p < 0.001) [127].

#### Double‐lumen tracheal tubes

Given their increased diameter, deeper insertion and greater tracheal mucosal contact, double‐lumen tracheal tubes are expected to have higher rates of POST compared with single‐lumen tubes. We found seven studies that examined specific aspects of double‐lumen tube insertion or structure, all conducted in patients undergoing elective thoracic surgery [16, 17, 128, 129, 130, 131, 132].

Three studies examined the effect of softening double‐lumen tubes by immersing them in saline at 40°C [16, 128] or 50°C [129] for 10 min before insertion. All reported reduced POST on postoperative day one and reduced observed vocal cord injury based on visual assessment by a blinded observer. Only one study examined POST in PACU following thermal softening, finding comparable rates of reduction in POST at 1 and 6 h compared with 24 h [129]; however, the reported advantages on postoperative days two and three were inconsistent [16, 128] with substantial risk of bias in two of the studies (online Supporting Information Figure S1) [128, 129]. Another approach to softening the structure of the tracheal tube was the use of a silicon double lumen tube (SILBRONCHO™; Fugi, Tokyo, Japan), which has a more flexible tip and different curvature from a traditional double‐lumen tube. This study was judged to be at low risk of bias and found that the silicon double lumen tube had a lower incidence of POST at 1 h and 24 h [17].

An alternative approach to choosing the size of a left‐sided double‐lumen tube, which permitted substantially smaller tubes (37‐ or 39‐Fr tubes in the intervention group), resulted in a lower incidence of POST immediately after tracheal extubation, but not at 24 h [130].

Different insertion techniques have also been investigated, with jaw thrust given during tracheal intubation and exchanging the stylet for a fibreoptic bronchoscope after passing through the glottis to guide bronchial placement both reducing evidence of visible vocal cord injury and POST severity at 1 h and 24 h [131, 132].

### Non‐pharmacological interventions – supraglottic airway devices

A total of 32 studies were identified which investigated POST after SAD insertion. Overall, the quality of these studies was poor, with varying risk of bias (online Supporting Information Figure S1). Cuff pressure interventions were well studied, but other interventions were limited.

#### Comparison of device

Few studies compared different devices: one showed reduced POST at all time points having compared two SADs; two studies compared devices but essentially studied differences in cuff pressure; and one compared a single‐use and a reusable version of a historical device. Kihara et al. compared the LMA Fastrach™ (Teleflex Medical) and the LMA Classic in women undergoing gynaecological surgery and showed that the LMA Fastrach increased the incidence of POST at 2 h, 24 h and 48 h [133]. Deepak et al. compared three types of SAD in patients having laparoscopic surgery: i‐gel® (Intersurgical, Wokingham, UK); AuraGain™ (Ambu, Ballerup, Denmark) with a cuff pressure set at 60 cmH_2_O; and AuraGain with a cuff pressure set at 25 cmH_2_O [134]. There was no difference in the incidence of POST (5.7% vs. 17.9% vs. 14.9%, respectively, p = 0.135), but dysphagia was noted in four patients allocated to the AuraGain high pressure group [134].

Wong et al. compared the Ultra CPV™ (AES, Inc., Black Diamond, WA, USA), which has an inbuilt cuff pressure indicator, and the LMA Classic [135]. The Ultra CPV had lower incidences of POST at 1 h, 2 h and 24 h. Although well conducted, the study reported that reduced cuff pressure reduced the incidence of POST rather than the device itself; mean cuff pressures at 5 min were 60 cmH_2_O vs. 118 cmH_2_O in patients allocated to the Ultra CPV and LMA Classic groups, respectively.

A comparison of the LMA ProSeal™ (Teleflex Medical) and LMA Classic in children aged 6–12 y found a lower incidence of POST at 6 h with the use of the LMA ProSeal, although the devices used appeared to be smaller on average in the intervention group [136].

Overall, there is insufficient evidence to recommend a particular SAD definitively, and we suggest SADs are chosen based on user familiarity, availability of device, individual patient factors and cost.

#### Supraglottic airway device cuff pressure

Cuff pressure was the most explored intervention in SADs to reduce the incidence and severity of POST. Many current SAD manufacturers recommend maintaining cuff pressure < 60 cmH_2_O; the included studies assessed a range of devices, which either compared a defined cuff pressure with routine care or a comparison of target pressures, with conflicting results.

The LMA Classic was the most studied, including devices derived from it and with a similar design. Burgard et al. showed no cases of POST in a sample of 200 patients when the cuff pressure of the LMA Classic was reduced to the minimum required for ventilation (approximately ≤ 50 cmH_2_O) [137]. In contrast, Rieger et al. did not show any difference in the incidence of POST when comparing a low (41 cmH_2_O) and high cuff pressure (245 cmH_2_O), although the incidence was high in both groups (50% vs. 42%, respectively) [138]. Seet et al. showed a reduction in the incidence of POST at 24 h when the LMA Classic cuff pressure was kept < 60 cmH_2_O compared with routine care [67]. Kang et al. also showed a significant reduction in the incidence of POST at 24 h with the LMA Supreme cuff pressure kept at 25 cmH_2_O compared with 60 cmH_2_O (3/49 vs 12/49, p = 0.012) [68]. Neither study showed a benefit in terms of reducing early POST. Conversely, Jeon et al. showed a reduced incidence of POST at 1 h but not at 24 h with the LMA Classic cuff pressure regulated at 28 cmH_2_O compared with 74 cmH_2_O [139].

One of the few studies that evaluated the incidence of POST beyond 24 h was a small study of 30 patients using the Well Lead™ SAD (Well Lead™ Medical Co. Ltd., Guangzhou, China), a first‐generation SAD similar to the LMA Classic [69]. The incidence of POST was reduced at 24 h and 48 h when using a minimum effective cuff pressure compared with usual care (pressure range 56–63 cmH_2_O vs. 126–139 cmH_2_O, respectively) [69]. Waruingi et al. showed a reduction in the incidence of POST at 2 h, 6 h and 12 h when comparing low cuff pressure of 30–32 cmH_2_O vs. routine care with the AuraOnce™ SAD (Ambu®); however, the study was at high risk of bias due to missing outcome data [140]. Limiting the cuff pressure in the LMA Unique™ to 60 cmH_2_O reduced the incidence and severity of POST at 24 h in adults aged > 65 y [141].

For studies using other SADs not based on the LMA Classic, there is limited evidence that cuff pressure reduction affects the incidence of POST. Joe et al. compared mean pressures of 60 cmH_2_O with 24 cmH_2_O using the CobraPLA® (Engineered Medical Systems, Inc., Indianapolis, IN, USA) and showed a reduced rate of moderate severity POST at 1 h, but the effect did not extend to other severity scores or at 24 h [142]. Similar results were found in a study of the LMA ProSeal with the cuff inflated to just above oropharyngeal leak pressure compared with cuff pressure adjusted to < 60 cmH_2_O [143]. There was no difference in the incidence of POST at any time point up to 24 h, but there was also no significant difference in cuff pressure between groups (mean (SD) 68 (26.8) cmH_2_O vs. 72 (28.7) cmH_2_O).

#### Supraglottic airway device factors

Five studies were identified which assessed different SAD insertion and anaesthesia maintenance techniques and their effects on the incidence of POST. Four studies showed no difference across treatment groups which included humidified vs. non‐humidified anaesthetic circuits [144]; external laryngeal lift with pre‐inflated cuff compared to standard insertion with and without inflated cuff [145]; the use of laryngoscope‐guided vs. standard insertion [146]; and the effect of varying concentrations of nitrous oxide plus spontaneous vs. controlled ventilation [144, 145, 146, 147]. However, these latter studies reported SAD cuff pressures > 120 cmH_2_O, which may have masked any effect of the interventions. Li et al. showed the incidence of POST was significantly reduced in PACU when the pilot balloon blocker was left in place during removal of the LMA Supreme, thereby keeping the cuff partially inflated [148]. The incidence of late POST was not reported.

### Pharmacological interventions – supraglottic airway devices

#### Lidocaine

Lidocaine was investigated in five studies, all with different treatment methods and/or comparators. Chandra et al. allocated 128 patients randomly to either nebulised lidocaine 1.5 mg.kg^‐1^ or intravenous dexamethasone 10 mg and showed no difference in the incidence of POST at 2 h [149]. Varying results have been reported with topical lidocaine gargles, jellies and lozenges. There was no difference between gargle and topical application of lidocaine to the SAD in the incidence of POST at 1 h [150]. A pre‐operative lozenge that included lidocaine reduced the incidence of POST at 30 min when compared with placebo, but the benefit did not extend to 24 h [151]. Taghavi Gilani et al. compared four groups: saline wash of the SAD (device not specified); saline oral wash before emergence; lidocaine gel applied to the SAD before insertion; and control [152]. The incidence and severity of POST did not differ between groups. Finally, Kiran et al. compared 2% lidocaine gel to 0.05% betamethasone gel to lubricate the LMA ProSeal [153]. There were no cases of mild POST reported at 24 h in patients allocated to the betamethasone group compared with 5/30 in those allocated to the lidocaine gel group (p = 0.036).

#### Non‐steroidal anti‐inflammatory drugs

Three studies investigated the effect of topical application of benzydamine. Two studies looking at short‐term incidence of POST differed in how benzydamine was applied: one involved topical application to the pharynx at 5 min and 30 min before induction; and the other involved direct application to the SAD. Both reported significant decreases in the incidence of POST at 4 h [154, 155]. A chlorhexidine‐benzydamine spray applied 15 min before induction decreased the incidence of early (1 h), but not late, (6–24 h) POST [156]. Uzture et al. compared a flurbiprofen 8.75 mg lozenge with a placebo and showed reduced POST severity at 30 min, which was not maintained at any later time points [157].

#### Others

Other interventions studied included different formulations of turmeric lozenges; chewing gum; and superior laryngeal nerve block. Naseem et al. compared two turmeric‐containing lozenges (turmeric vs. turmeric with menthol and eucalyptus) [158]. The turmeric/menthol/eucalyptus lozenge reduced the incidence across all time points up to 24 h, but the study reported a very high global incidence of POST (77–96% at 30 min) [158]. Rashwan et al. showed a reduction in the incidence of POST with a 2 mg.kg^‐1^ tramadol gargle compared with control, but it is unclear if this was a local effect or secondary to systemic absorption [159]. Chewing gum may have other benefits, such as reduced ileus and postoperative nausea and vomiting, and was shown to reduce the incidence of moderate/severe POST at 24 h in a well‐conducted study that used a third‐generation SAD (Streamlined Liner of the Pharynx Airway, SLIPA™; Slipa Medical Ltd., London, UK) [160]. Finally, Lv et al. compared bilateral superior laryngeal nerve blocks vs. SAD lubrication with tetracaine syrup and showed a reduced incidence and severity of POST at 30 min, 6 h and 24 h [161]. However, this study was not powered for safety outcomes.

## Discussion

Our systematic review shows there remains a paucity of high‐quality evidence to prevent a common clinical problem; we were only able to identify five randomised controlled trials that were at low risk of bias [13, 14, 15, 16, 17]. This is surprising given the frequency of POST and its importance to patients, and could be due to several factors including no clear agreed‐upon definition of POST; variance in measurement mechanisms; and POST being perceived as a mild, self‐limiting condition and thus not a research priority.

Postoperative pain, of which POST is a common cause, is one of the leading reasons for patient dissatisfaction with their anaesthetic care and therefore should be considered important and worthy of high‐quality research [4, 162]. Avoidance of sore throat has been judged by patients to be a priority in terms of postoperative complications [163, 164], but this view is not shared by anaesthetists, who place greater importance on less frequent but more serious adverse outcomes (e.g. death, accidental awareness and peripheral nerve injury) [163, 165]. This may reflect that anaesthetists perceive POST as a self‐limiting, relatively minor complaint. However, POST can persist for up to 96 h in a small proportion of patients [5], which may not allow them to resume eating and drinking [6, 7] and impede enhanced recovery peri‐operative care pathways (e.g. Drinking, Eating Mobilising – DrEaMing) [8]. In one study, around 30% of patients still complained of moderate to severe throat pain and difficulty in swallowing at 17–24 h postoperatively [7]. In a study of 220 patients having maxillofacial surgery, 81% complained of a sore throat and difficulty eating, which persisted for 3–7 days postoperatively [166].

Due to the heterogeneity of the patient population, surgical speciality, interventions and comparators in included studies, we were unable to perform a meta‐analysis or make strong recommendations. How POST is defined and its severity measured is also variable, which makes it challenging to compare interventions. What patients describe as ‘sore throat’ encompasses a wide range of experiences and diagnoses, including pharyngitis; laryngitis; tracheitis; cough; hoarseness; and dysphagia. However, there were some key interventions that we believe can be used in routine clinical practice, many of which can be implemented with minimal patient risk or cost (Table 1).

Choosing the smallest tracheal tube size that is clinically appropriate will help reduce the risk of POST [5]. In clinical practice, tracheal tube size is often selected generically based on patient sex, despite wide variation in tracheal dimensions. For many patients undergoing elective procedures, smaller tracheal tubes (e.g. internal diameter 6.0–6.5 mm) will allow effective positive pressure ventilation without increasing the risk of ventilator‐induced lung injury [167]. This aligns with the findings of a recent systematic review, albeit with the caveat that only six trials were included, all of which were at risk of bias [168].

Similarly, checking cuff pressure of tracheal tubes and SADs after insertion and then intra‐operatively at regular intervals will avoid excessively high cuff pressures, which increase the risk of POST. It is noteworthy that the pooled incidence of POST in the studies we included, in which measurement of cuff pressure was an eligibility criterion, had a lower incidence of POST (29.4% at 1 h and 16.4% at 24 h for tracheal intubation) than other studies where cuff pressure was not controlled, where the incidence of POST often exceeds 40% [2]. Not using a manometer to check cuff pressures of both tracheal tubes and SADs is difficult to justify given the tendency for airway operators to overinflate the cuff [22, 23].

There is a need for more well‐conducted, randomised controlled trials to define the role of pharmacological interventions more precisely and to determine the optimal dose and route. It is important that any future studies reflect contemporary anaesthetic practice, notably the increasing use of videolaryngoscopy in both the emergent and elective settings [169, 170, 171, 172], and the use of second‐ and third‐generation SADs [173]. Given the potential benefits of these interventions to increase first pass tracheal intubation success and the absence of an inflatable cuff, respectively, it is likely that both will reduce the incidence of POST. It is also important that future work includes patients that reflect the current clinical surgical population [174], namely the inclusion of older patients and those with comorbid conditions. A final consideration is avoiding nitrous oxide and the attendant risk of increasing cuff pressures over the course of a case. Given the move towards eliminating this drug from anaesthetic practice due to environmental concerns in both high‐, low‐ and middle‐income countries [175, 176], the use of air/oxygen‐based anaesthetics in future studies is recommended.

This review has several limitations, most notably the low quality of the evidence which precluded us from making strong recommendations. The heterogeneity of included studies also did not allow pooling of the data and meta‐analysis. Furthermore, we only included studies written in English, as there were no translated versions available for those published in other languages. However, by only including studies which controlled for a major confounder for POST development, namely airway device cuff pressure, we have derived a more accurate incidence of POST and have been able to suggest areas that current clinical practice and future research could target. We have progressed the evidence base from a previous update by providing risk of bias assessments and capturing more studies [1]. This has provided a wider synthesis of the current evidence base for interventions to decrease POST, rather than simply identifying risk factors [1]. We have highlighted the paucity of randomised controlled trials at a low risk of bias.

In conclusion, given the high incidence of POST, it is surprising that there are so few randomised controlled trials at low risk of bias that address a frequent postoperative complication. This may reflect the mismatch between patient and anaesthetist priorities [163]. Further research will add value to this area if studies control for confounders adequately.

## Supporting information


**Appendix S1.** MEDLINE and Embase search strategies.
**Appendix S2.** Statistical code.

**Figure S1.** Risk of bias assessment of included studies.

**Figure S2.** Forest plots of the incidence of postoperative sore throat at 1 h and 24 h for studies involving tracheal tubes and supraglottic airway devices.

**Table S1.** Data extraction template.
**Table S2.** Characteristics of included studies.
