# Supplementary material for: Postoperative sore throat: a systematic review*
Source: Anaesthesia. 2025 Oct 28;81(1):116–33. doi: 10.1111/anae.70048 (PMC12747620; doi:10.1111/anae.70048)
Supplement: Supplementary file 1 — Appendix S1. MEDLINE and Embase search strategies. Appendix S2. Statistical code. [file ANAE-81-116-s001.docx]

**Appendix S1.** MEDLINE and EMBASE Search Strategy

Embase <1974 to 2025 January 03>

Ovid MEDLINE(R) ALL <1946 to January 03, 2025>

1 pharyngitis.tw. 15391

2 (throat* adj2 (sore or inflam* or infect*)).tw. 22603

3 pharyngitis/ 27926

4 1 or 2 or 3 51980

5 postoperative complication/ or postoperative pain/ 989757

6 (post-op* or postop* or post-surg* or postsurg*).tw. 2024862

7 ((follow* or after) adj2 (surg* or oper*)).tw. 1233526

8 5 or 6 or 7 3181656

9 (prevent* or prophyla* or reduc* or incidence or rate).mp. [mp=ti, ab, hw, tn, ot, dm, mf, dv, kf, fx, dq, bt, nm, ox, px, rx, ui, sy, ux, mx] 22939503

10 (trial* or investig* or stud* or rev*).mp. [mp=ti, ab, hw, tn, ot, dm, mf, dv, kf, fx, dq, bt, nm, ox, px, rx, ui, sy, ux, mx] 52415121

11 4 and 8 and 9 and 10 2587

12 remove duplicates from 11 1798*

*second iteration of search strategy, combined with original as documented on PROSPERO. Duplicates removed, accounting for discrepancy with PRISMA flow diagram.

**Appendix S2.** R code used to perform meta-analysis of incidence of POST across tracheal tube and SAD studies.

###############################################################################

# Script 1: Meta‑Analysis Using the Logit Transformation (rma.glmm)

###############################################################################

# --- Step 1: Load Required Packages ---

if (!requireNamespace("readxl", quietly = TRUE)) install.packages("readxl")

if (!requireNamespace("dplyr", quietly = TRUE)) install.packages("dplyr")

if (!requireNamespace("metafor", quietly = TRUE)) install.packages("metafor")

if (!requireNamespace("stringr", quietly = TRUE)) install.packages("stringr")

library(readxl)

library(dplyr)

library(metafor)

library(stringr)

cat("metafor version:", as.character(packageVersion("metafor")), "\n\n")

# --- Step 2: Load Excel File & Select Sheet ---

file_path <- file.choose()

sheets <- excel_sheets(file_path)

sheet_name <- select.list(sheets, title = "Please select an Excel sheet", graphics = TRUE)

df <- as.data.frame(read_excel(file_path, sheet = sheet_name))

# --- Step 3: Rename and Convert Columns ---

names(df) <- str_trim(names(df))

names(df)[names(df) == "Study ID"] <- "StudyID"

numeric_cols <- c("Result data: POST Dichotomous (1 (1H)) Events",

"Result data: POST Dichotomous (1 (1H)) Total",

"Result data: POST Dichotomous (10(24H)) Events",

"Result data: POST Dichotomous (10(24H)) Total")

df[numeric_cols] <- lapply(df[numeric_cols], function(x) as.numeric(as.character(x)))

df <- df %>%

rename(

D1_events = `Result data: POST Dichotomous (1 (1H)) Events`,

D1_total = `Result data: POST Dichotomous (1 (1H)) Total`,

D10_events = `Result data: POST Dichotomous (10(24H)) Events`,

D10_total = `Result data: POST Dichotomous (10(24H)) Total`

)

# --- Step 4: Aggregate by StudyID ---

df_study_level <- df %>%

group_by(StudyID) %>%

summarise(

D1_events = sum(D1_events, na.rm = TRUE),

D1_total = sum(D1_total, na.rm = TRUE),

D10_events = sum(D10_events, na.rm = TRUE),

D10_total = sum(D10_total, na.rm = TRUE)

) %>% ungroup()

df_study_1hr <- df_study_level %>% filter(D1_total > 0)

df_study_24hr <- df_study_level %>% filter(D10_total > 0)

xi1 <- df_study_1hr$D1_events

ni1 <- df_study_1hr$D1_total

xi2 <- df_study_24hr$D10_events

ni2 <- df_study_24hr$D10_total

# --- Step 5: Run Meta-Analysis ---

if(length(xi1) >= 2) {

res_1h <- rma.glmm(measure = "PLO", xi = xi1, ni = ni1,

add = 0.5, method = "ML", nAGQ = 7, test = "z")

pred_1h <- predict(res_1h, transf = transf.ilogit)

cat("1‑Hour Outcome:\n")

cat(" Pooled incidence:", round(pred_1h$pred * 100, 1), "%\n")

cat(" 95% CI:", round(pred_1h$ci.lb * 100, 1), "% to", round(pred_1h$ci.ub * 100, 1), "%\n\n")

}

if(length(xi2) >= 2) {

res_24h <- rma.glmm(measure = "PLO", xi = xi2, ni = ni2,

add = 0.5, method = "ML", nAGQ = 7, test = "z")

pred_24h <- predict(res_24h, transf = transf.ilogit)

cat("24‑Hour Outcome:\n")

cat(" Pooled incidence:", round(pred_24h$pred * 100, 1), "%\n")

cat(" 95% CI:", round(pred_24h$ci.lb * 100, 1), "% to", round(pred_24h$ci.ub * 100, 1), "%\n")

}

pdf(file='forestplot1.pdf') # Open PDF device with specific file name

forest(res_1h, atransf = transf.ilogit, shade = TRUE, at = transf.logit(c(0.01, 0.25, 0.5, 0.75, 0.99)),

       slab = df_study_1hr$StudyID)

dev.off() # Turn the PDF device off
