# Supplementary material for: Postoperative sore throat: a systematic review*
Source: Anaesthesia. 2025 Oct 28;81(1):116–33. doi: 10.1111/anae.70048 (PMC12747620; doi:10.1111/anae.70048)

**Figure S1.** Intention to treat - Cochrane Risk of Bias 2 Tool for Randomised Controlled Trial Results. Risk of bias assessment outcome reported in tabular fashion below.

Key:


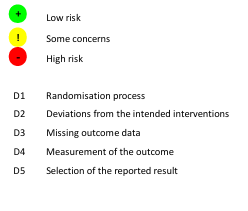


# *Tracheal Tube – Pharmacological*


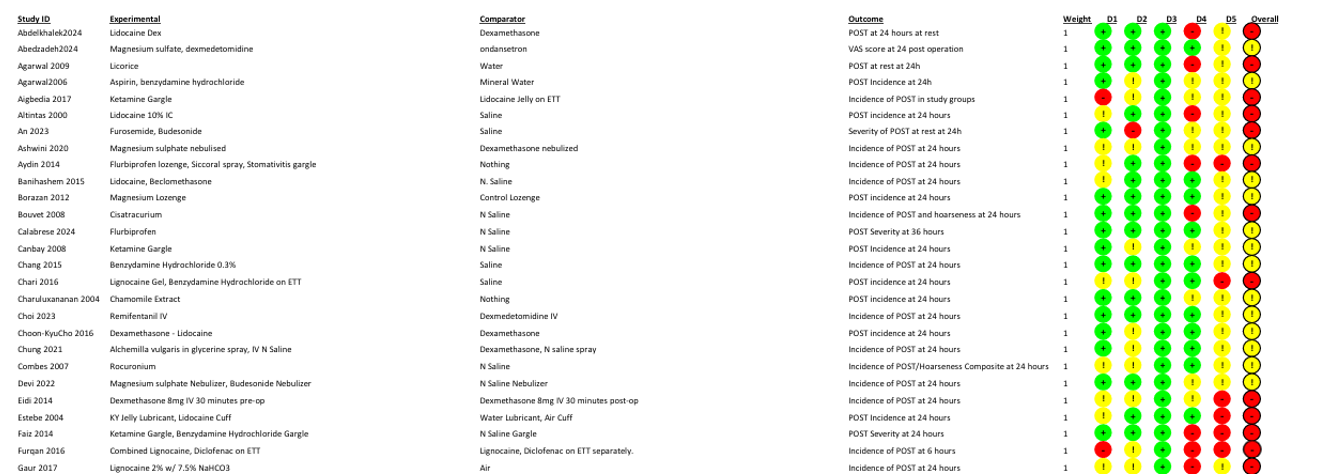


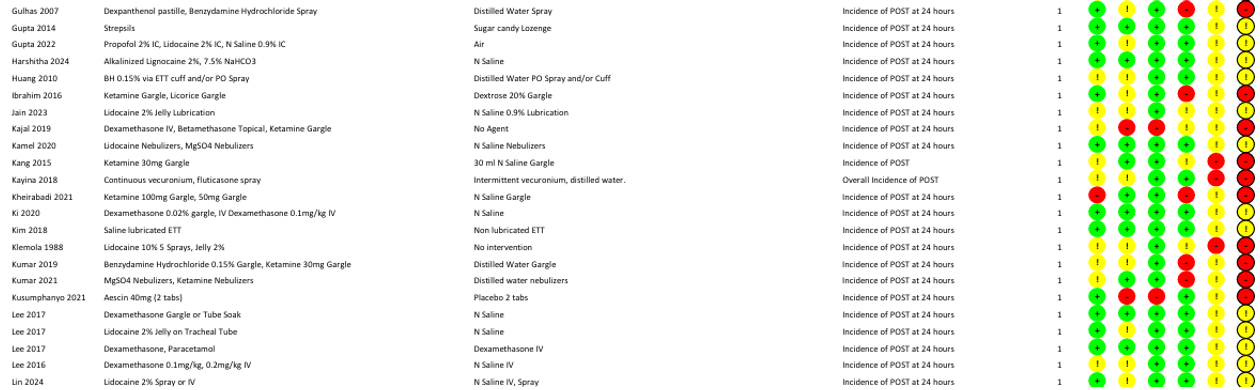


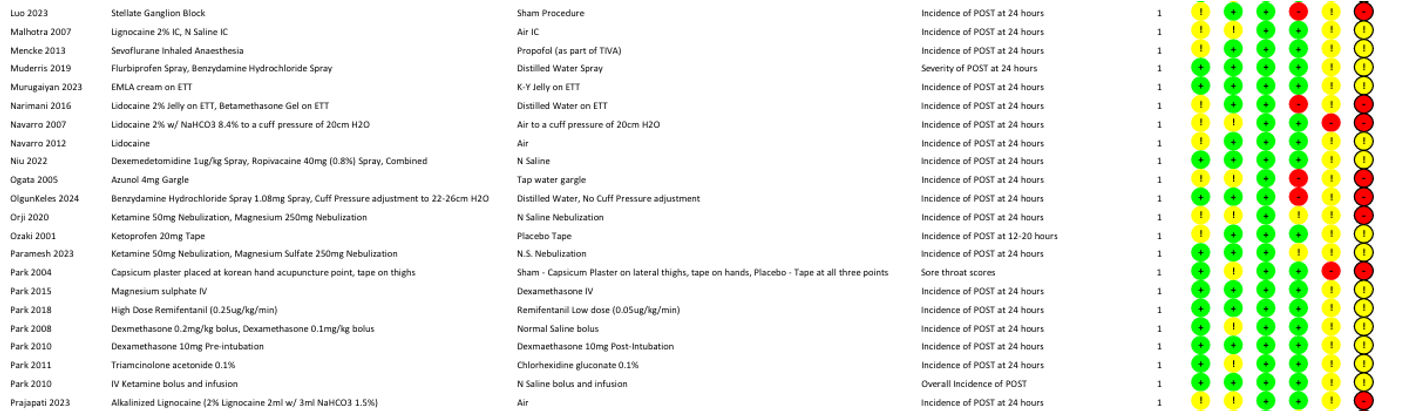


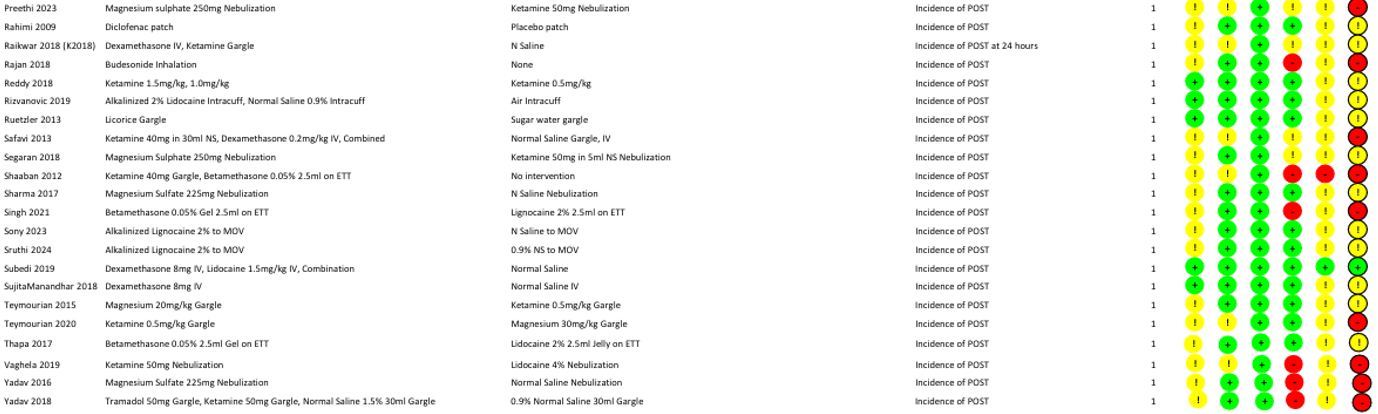


#
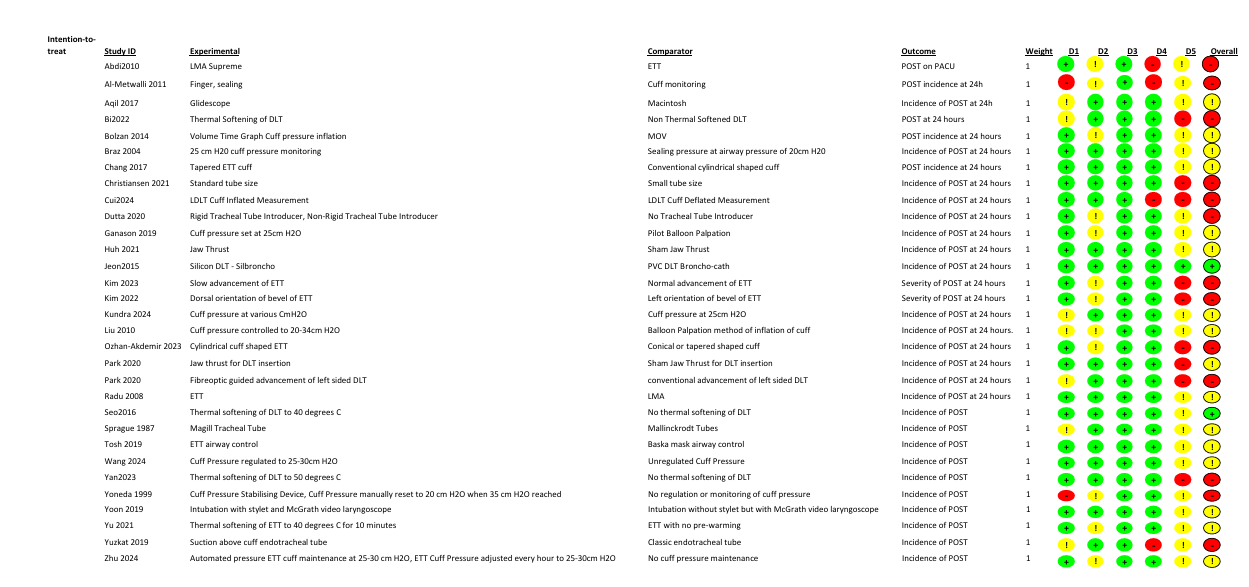
*Tracheal Tube – Non- pharmacological*

# *Supraglottic Airway Devices*


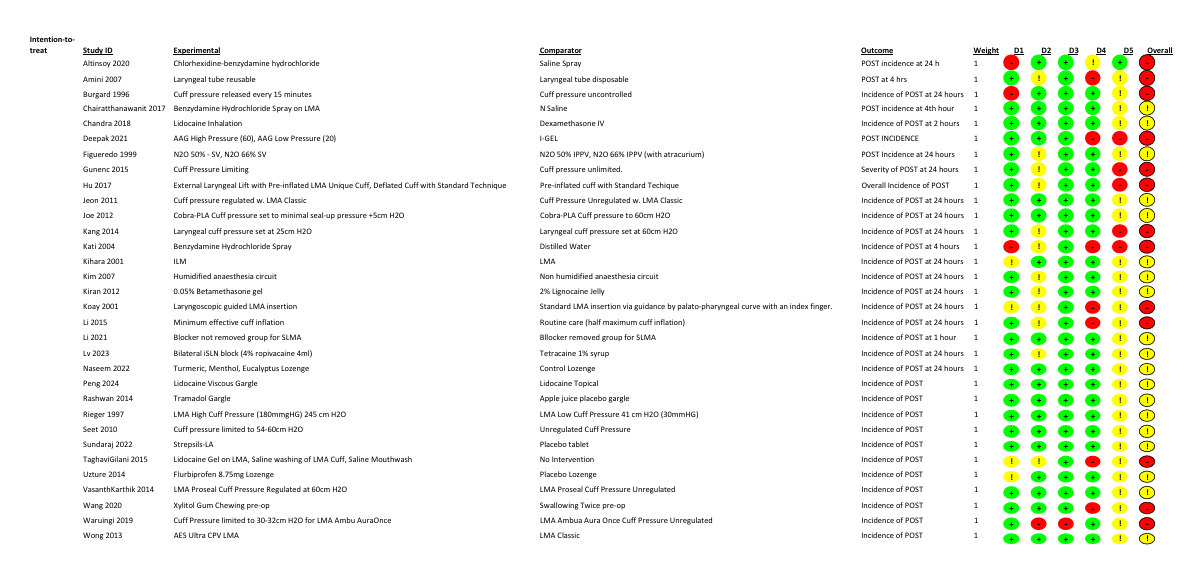


# *Paediatric*


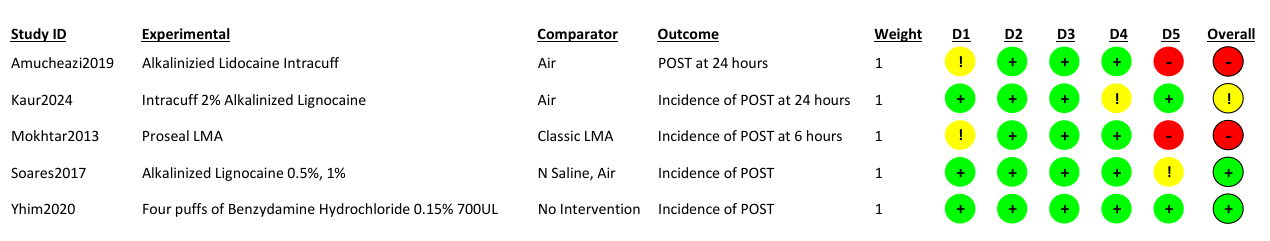

Supplement: Supplementary file 2 — Figure S1. Risk of bias assessment of included studies. [file ANAE-81-116-s003.docx]
