# Supplementary material for: Postoperative sore throat: a systematic review*
Source: Anaesthesia. 2025 Oct 28;81(1):116–33. doi: 10.1111/anae.70048 (PMC12747620; doi:10.1111/anae.70048)
Supplement: Supplementary file 3 — Figure S2. Forest plots of the incidence of postoperative sore throat at 1 h and 24 h for studies involving tracheal tubes and supraglottic airway devices. [file ANAE-81-116-s002.pptx]

## Slide 1
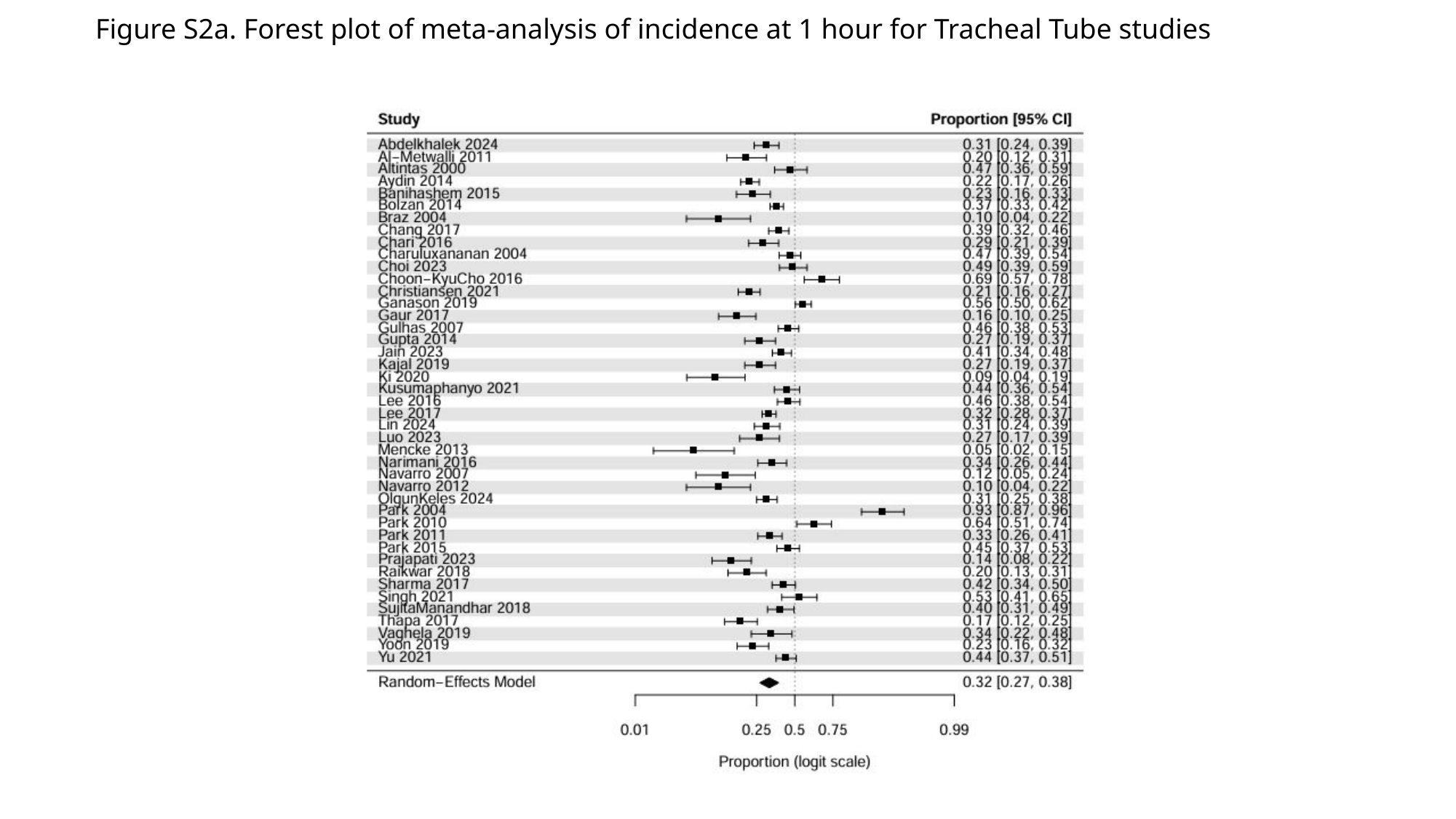

Figure S2a. Forest plot of meta-analysis of incidence at 1 hour for Tracheal Tube studies

## Slide 2
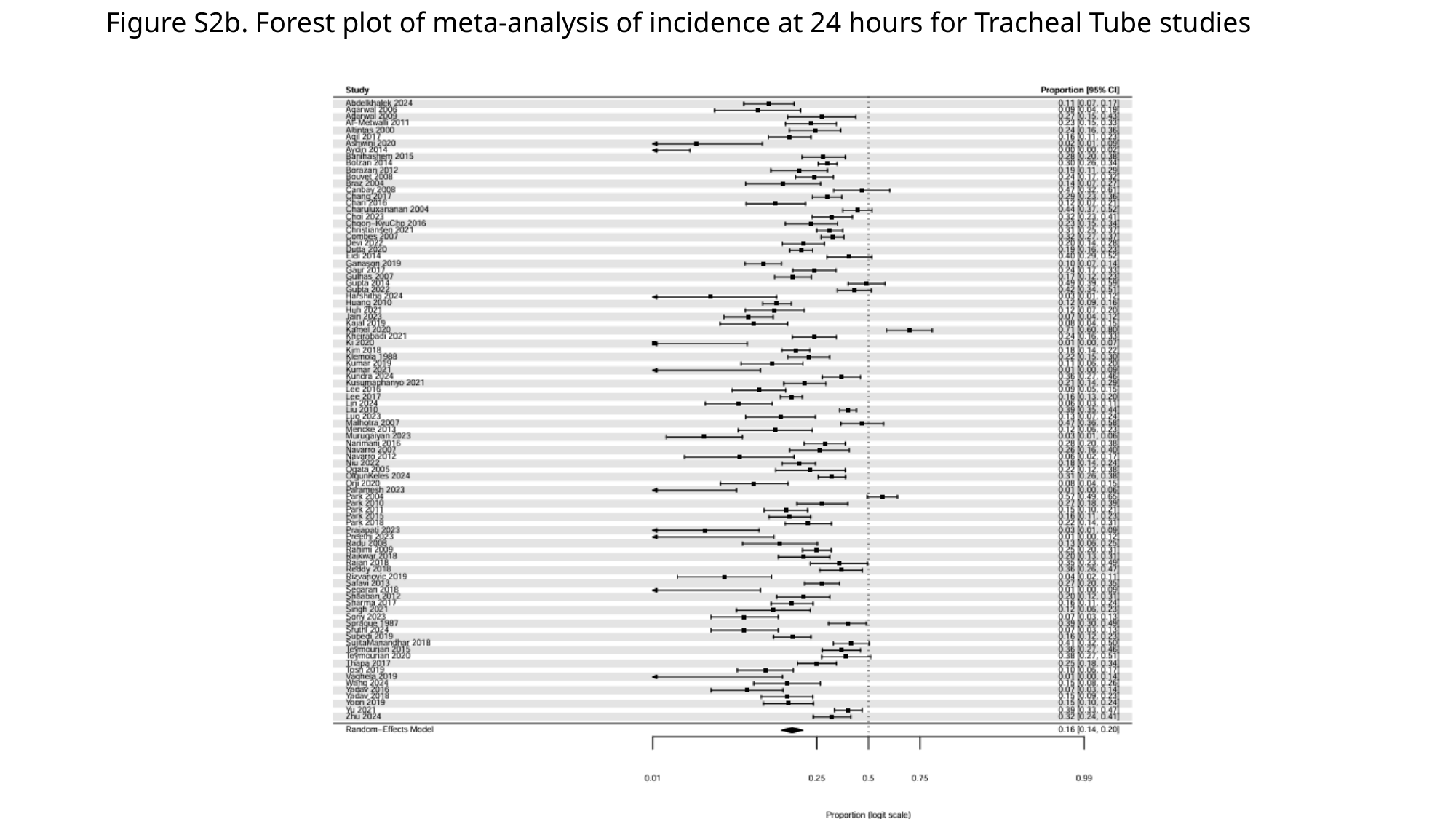

Figure S2b. Forest plot of meta-analysis of incidence at 24 hours for Tracheal Tube studies

## Slide 3
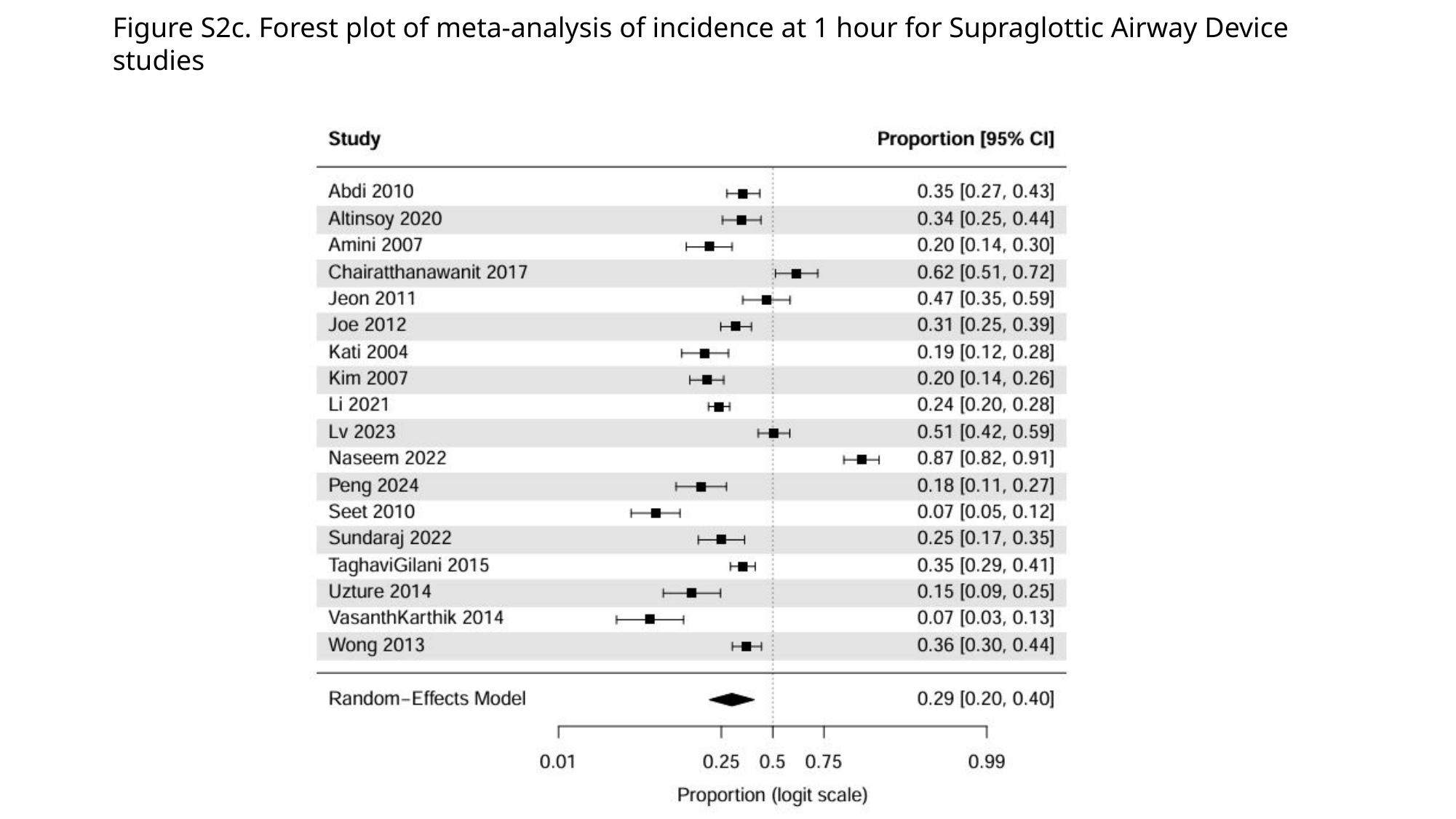

Figure S2c. Forest plot of meta-analysis of incidence at 1 hour for Supraglottic Airway Device studies

## Slide 4
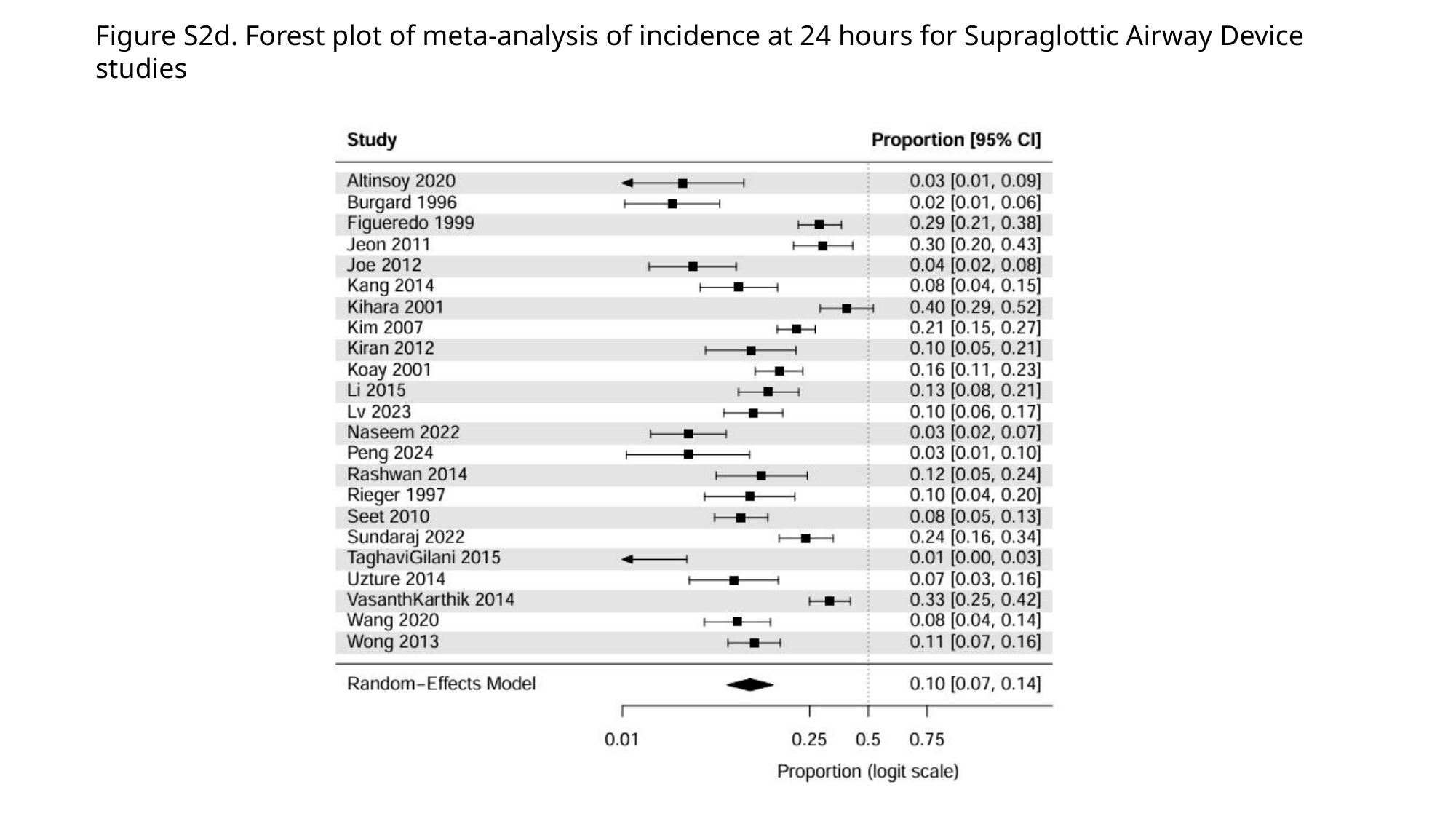

Figure S2d. Forest plot of meta-analysis of incidence at 24 hours for Supraglottic Airway Device studies
