# Supplementary material for: Postoperative sore throat: a systematic review*
Source: Anaesthesia. 2025 Oct 28;81(1):116–33. doi: 10.1111/anae.70048 (PMC12747620; doi:10.1111/anae.70048)
Supplement: Supplementary file 4 — Table S1. Data extraction template. Table S2. Characteristics of included studies. [file ANAE-81-116-s004.docx]

**Table S1.** Data extraction template. Items listed below were extracted from all included studies if possible, otherwise if not reported they were recorded as such.

| **Identification** | Sponsorship Source  Country  Setting  Name of Author  Institution  Classification of Study |
| --- | --- |
| **Methods** | Design  Group  Length of follow-up (hours)  Method of capturing POST (VAS/Questions/ Severity Index)  Tracheal tube cuff pressure (cm H_2_O)  SAD Cuff Pressure (cmH_2_O)  Size of airway used for Men (mm for Tracheal tube/number for SAD)  Size of airway used for women (mm for tracheal tube/ number for SAD)  Method of airway control  Method of recruitment of patients  Aim of Study (Comparison of Device, Pharmacotherapy etc.)  Method of statistical calculation  Study design (PRCT vs CRCT)  Randomisation Method  Sequence generation  Allocation concealment |
| **Population** | Total Sample size  Number of withdrawals  Reason for withdrawals  Surgical Cohort  Mean, SD of Age, BMI, Duration of anaesthetic, Height and Weight  Ethnicity  Smoking Status (N)  Sex (N of females) |
| **Intervention/ Control** | Number of participants allocated  Dose, frequency, associated monitoring, timing of dose pre-intubation, timing of dose post-intubation, route of medication duration of intervention  Airway device chosen  Inflation of cuff amount (cmH_2_O) |
| **Outcomes** | Continuous measures of POST (mean +/- SD, N in group or mild, moderate, severe)  Incidence of POST (N / Total in Group) |

CRCT, crossover randomised controlled trial. N, total number. PRCT, parallel randomised controlled trial. SAD, supraglottic airway devices. SD, standard deviation.

**Table S2.** Characteristics of included studies

## Tracheal Tube - Pharmacological

Abdelkhalek 2024 – Does the combination of intravenous lidocaine and dexamethasone reduce the incidence of postoperative sore throat? A randomized controlled trial

| **Characteristic** | **Value** |
| --- | --- |
| Country | Egypt |
| Total sample size (N) | 140 |
| Female (N) | 51 |
| Smoking status (N) | 0 |
| Length of follow-up (h) | 24 |
| Airway control | ETT |
| Cuff pressure (cm H₂O) | ≤ 17 |
| Intubation attempts allowed | ≤ 1 |
| Age (mean ± SD, yr) | 37.63 ± 8.90 |
| BMI (mean ± SD) | 26.21 ± 1.74 |
| Duration of anaesthetic (mean ± SD) | 213.75 ± 42.19 |
| Height (cm mean ± SD) | 174 ± 6.08 |
| Weight (kg mean ± SD) | 80.05 ± 6.64 |

Abedzadeh 2024 – Comparison of adding magnesium sulfate, dexmedetomidine and ondansetron to lidocaine for gargling before laryngoscopy and endotracheal intubation to prevent sore throat: A randomized clinical trial

| **Characteristic** | **Value** |
| --- | --- |
| Country | Iran |
| Total sample size (N) | 105 |
| Female (N) | NR |
| Smoking status (N) | NR |
| Length of follow-up (h) | 24 |
| Airway control | ETT |
| Cuff pressure (cm H₂O) | ≤ 25 |
| Intubation attempts allowed | ≤ 1 |
| Age (mean ± SD, yr) | 42.12 ± 6.60 |
| BMI (mean ± SD) | NR |
| Duration of anaesthetic (mean ± SD) | NR |
| Height (cm mean ± SD) | NR |
| Weight (kg mean ± SD) | NR |

Agarwal 2006 – An evaluation of the efficacy of aspirin and benzydamine hydrochloride gargle for attenuating postoperative sore throat: A prospective, randomized, single-blind study

| **Characteristic** | **Value** |
| --- | --- |
| Country | India |
| Total sample size (N) | 60 |
| Female (N) | 58 |
| Smoking status (N) | NR |
| Length of follow-up (h) | 24 |
| Airway control | ETT |
| Cuff pressure (cm H₂O) | 18–22 |
| Intubation attempts allowed | ≤ 1 |
| Age (mean ± SD, yr) | 41.81 ± 14.66 |
| BMI (mean ± SD) | NR |
| Duration of anaesthetic (mean ± SD) | 158.13 ± 86.95 |
| Height (cm mean ± SD) | 155.76 ± 8.01 |
| Weight (kg mean ± SD) | 52.64 ± 9.30 |

Agarwal 2009 – An evaluation of the efficacy of licorice gargle for attenuating postoperative sore throat: A prospective, randomized, single-blind study

| **Characteristic** | **Value** |
| --- | --- |
| Country | India |
| Total sample size (N) | 40 |
| Female (N) | 9 |
| Smoking status (N) | NR |
| Length of follow-up (h) | 24 |
| Airway control | ETT |
| Cuff pressure (cm H₂O) | 18–22 |
| Intubation attempts allowed | ≤ 1 |
| Age (mean ± SD, yr) | 43.06 ± 15.09 |
| BMI (mean ± SD) | NR |
| Duration of anaesthetic (mean ± SD) | 133.97 ± 13.40 |
| Height (cm mean ± SD) | 167.36 ± 6.25 |
| Weight (kg mean ± SD) | 56.91 ± 7.80 |

Aigbedia 2017 – A comparative study of ketamine gargle and lidocaine jelly application for the prevention of postoperative throat pain following general anaesthesia with endotracheal intubation

| **Characteristic** | **Value** |
| --- | --- |
| Country | Nigeria |
| Total sample size (N) | 150 |
| Female (N) | 96 |
| Smoking status (N) | NR |
| Length of follow-up (h) | 72 |
| Airway control | ETT |
| Cuff pressure (cm H₂O) | 25 |
| Intubation attempts allowed | NR |
| Age (mean ± SD, yr) | 39.19 ± 11.43 |
| BMI (mean ± SD) | 26.99 ± 3.96 |
| Duration of anaesthetic (mean ± SD) | 95.70 ± 33.87 |
| Height (cm mean ± SD) | 158.46 ± 7.28 |
| Weight (kg mean ± SD) | 67.78 ± 10.72 |

Altintas 2000 – Lidocaine 10% in the endotracheal tube cuff: Blood concentrations, haemodynamic and clinical effects

| **Characteristic** | **Value** |
| --- | --- |
| Country | Turkey |
| Total sample size (N) | 70 |
| Female (N) | 39 |
| Smoking status (N) | 0 |
| Length of follow-up (h) | 24 |
| Airway control | ETT |
| Cuff pressure (cm H₂O) | Variable |
| Intubation attempts allowed | NR |
| Age (mean ± SD, yr) | 30.33 ± 11.31 |
| BMI (mean ± SD) | NR |
| Duration of anaesthetic (mean ± SD) | 125.42 ± 18.77 |
| Height (cm mean ± SD) | NR |
| Weight (kg mean ± SD) | 68.93 ± 16.82 |

An 2023 – Effects of postoperative nebulized furosemide and budesonide on postoperative sore throat in patients undergoing maxillofacial surgery: A randomized controlled trial

| **Characteristic** | **Value** |
| --- | --- |
| Country | China |
| Total sample size (N) | 90 |
| Female (N) | 73 |
| Smoking status (N) | NR |
| Length of follow-up (h) | 48 |
| Airway control | NTT |
| Cuff pressure (cm H₂O) | 20–25 |
| Intubation attempts allowed | ≤ 2 |
| Age (mean ± SD, yr) | 31.37 ± 8.23 |
| BMI (mean ± SD) | NR |
| Duration of anaesthetic (mean ± SD) | 164.70 ± 42.14 |
| Height (cm mean ± SD) | NR |
| Weight (kg mean ± SD) | NR |

Ashwini 2020 – Comparative study of dexamethasone nebulisation with magnesium sulphate nebulisation in preventing post operative sore throat following endotracheal intubation

| **Characteristic** | **Value** |
| --- | --- |
| Country | India |
| Total sample size (N) | 90 |
| Female (N) | 29 |
| Smoking status (N) | NR |
| Length of follow-up (h) | 24 |
| Airway control | ETT |
| Cuff pressure (cm H₂O) | 20–22 |
| Intubation attempts allowed | ≤ 2 |
| Age (mean ± SD, yr) | 37.27 ± 9.52 |
| BMI (mean ± SD) | NR |
| Duration of anaesthetic (mean ± SD) | 116.38 ± 37.11 |
| Height (cm mean ± SD) | NR |
| Weight (kg mean ± SD) | NR |

Aydin 2014 – Comparison of Siccoral spray, Stomatovis gargle, and Strefen lozenges on postoperative sore throat

| **Characteristic** | **Value** |
| --- | --- |
| Country | Turkey |
| Total sample size (N) | 320 |
| Female (N) | 31 |
| Smoking status (N) | NR |
| Length of follow-up (h) | 24 |
| Airway control | ETT |
| Cuff pressure (cm H₂O) | 18–22 |
| Intubation attempts allowed | ≤ 1 |
| Age (mean ± SD, yr) | 59.50 ± 12.88 |
| BMI (mean ± SD) | NR |
| Duration of anaesthetic (mean ± SD) | 94.50 ± 46.14 |
| Height (cm mean ± SD) | NR |
| Weight (kg mean ± SD) | NR |

Banihashem 2015 – Prophylactic effects of lidocaine or beclomethasone spray on post-operative sore throat and cough after orotracheal intubation

| **Characteristic** | **Value** |
| --- | --- |
| Country | Iran |
| Total sample size (N) | 90 |
| Female (N) | 270 |
| Smoking status (N) | NR |
| Length of follow-up (h) | 24 |
| Airway control | ETT |
| Cuff pressure (cm H₂O) | 20–25 |
| Intubation attempts allowed | ≤ 1 |
| Age (mean ± SD, yr) | 42.56 ± 10.75 |
| BMI (mean ± SD) | NR |
| Duration of anaesthetic (mean ± SD) | 110.23 ± 30.12 |
| Height (cm mean ± SD) | NR |
| Weight (kg mean ± SD) | 72.54 ± 8.39 |

Borazan 2012 – Oral magnesium lozenge reduces postoperative sore throat: a randomized, prospective, placebo-controlled study

| **Characteristic** | **Value** |
| --- | --- |
| Country | Turkey |
| Total sample size (N) | 70 |
| Female (N) | 21 |
| Smoking status (N) | NR |
| Length of follow-up (h) | 24 |
| Airway control | ETT |
| Cuff pressure (cm H₂O) | 20–22 |
| Intubation attempts allowed | ≤ 1 |
| Age (mean ± SD, yr) | 39.50 ± 8.14 |
| BMI (mean ± SD) | NR |
| Duration of anaesthetic (mean ± SD) | 85.00 ± 10.58 |
| Height (cm mean ± SD) | NR |
| Weight (kg mean ± SD) | 57.50 ± 7.48 |

Bouvet 2008 – Laryngeal injuries and intubating conditions with or without muscular relaxation: An equivalence study

| **Characteristic** | **Value** |
| --- | --- |
| Country | France |
| Total sample size (N) | 129 |
| Female (N) | 129 |
| Smoking status (N) | NR |
| Length of follow-up | 30 days |
| Airway control | ETT |
| Cuff pressure (cm H₂O) | 25 |
| Intubation attempts allowed | ≤ 2 |
| Age (mean ± SD, yr) | 41.10 ± 14.06 |
| BMI (mean ± SD) | 23.79 ± 4.60 |
| Duration of anaesthetic (mean ± SD) | 112.44 ± 53.39 |
| Height (cm mean ± SD) | NR |
| Weight (kg mean ± SD) | NR |

Calabrese 2024 – Flurbiprofen in the subglottic space to prevent postoperative sore throat after cardiac surgery: A randomized double-blind study

| **Characteristic** | **Value** |
| --- | --- |
| Country | Italy |
| Total sample size (N) | 70 |
| Female (N) | 11 |
| Smoking status (N) | 0 |
| Length of follow-up (h) | 36 |
| Airway control | ETT |
| Cuff pressure (cm H₂O) | 25 |
| Intubation attempts allowed | NR |
| Age (mean ± SD, yr) | 68.83 ± 6.97 |
| BMI (mean ± SD) | 27.27 ± 3.99 |
| Duration of anaesthetic (mean ± SD) | 842.00 ± 202.68 |
| Height (cm mean ± SD) | NR |
| Weight (kg mean ± SD) | NR |

Canbay 2008 – Ketamine gargle for attenuating postoperative sore throat

| **Characteristic** | **Value** |
| --- | --- |
| Country | Turkey |
| Total sample size (N) | 46 |
| Female (N) | NR |
| Smoking status (N) | 26 |
| Length of follow-up (h) | 24 |
| Airway control | ETT |
| Cuff pressure (cm H₂O) | 18–22 |
| Intubation attempts allowed | ≤ 1 |
| Age (mean ± SD, yr) | 25.31 ± 4.41 |
| BMI (mean ± SD) | NR |
| Duration of anaesthetic (mean ± SD) | 54.89 ± 14.44 |
| Height (cm mean ± SD) | 163.32 ± 8.80 |
| Weight (kg mean ± SD) | 61.50 ± 10.16 |

Chang 2015 – Effect of prophylactic benzydamine hydrochloride on postoperative sore throat and hoarseness after tracheal intubation using a double-lumen endobronchial tube: a randomized controlled trial

| **Characteristic** | **Value** |
| --- | --- |
| Country | Korea (Republic) |
| Total sample size (N) | 95 |
| Female (N) | 31 |
| Smoking status (N) | 32 |
| Length of follow-up (h) | 24 |
| Airway control | DLT |
| Cuff pressure (cm H₂O) | 20 |
| Intubation attempts allowed | NR |
| Age (mean ± SD, yr) | 52.25 ± 12.38 |
| BMI (mean ± SD) | NR |
| Duration of anaesthetic (mean ± SD) | 175.50 ± 102.62 |
| Height (cm mean ± SD) | 166.00 ± 8.53 |
| Weight (kg mean ± SD) | 61.00 ± 9.51 |

Chari 2016 – Comparative study to analyze the incidence of sore throat, cough, and hoarseness of voice after general anesthesia with the use of topical benzydamine hydrochloride and 2% lignocaine gel with placebo

| **Characteristic** | **Value** |
| --- | --- |
| Country | India |
| Total sample size (N) | 90 |
| Female (N) | NR |
| Smoking status (N) | NR |
| Length of follow-up (h) | 24 |
| Airway control | ETT |
| Cuff pressure (cm H₂O) | 20–30 |
| Intubation attempts allowed | ≤ 1 |
| Age (mean ± SD, yr) | 38.58 ± 10.22 |
| BMI (mean ± SD) | NR |
| Duration of anaesthetic (mean ± SD) | 116.44 ± 44.87 |
| Height (cm mean ± SD) | 166.82 ± 8.62 |
| Weight (kg mean ± SD) | 60.31 ± 9.11 |

Charuluxananan 2004 – Effectiveness of lubrication of endotracheal tube cuff with chamomile-extract for prevention of postoperative sore throat and hoarseness

| **Characteristic** | **Value** |
| --- | --- |
| Country | Thailand |
| Total sample size (N) | 161 |
| Female (N) | 118 |
| Smoking status (N) | 34 |
| Length of follow-up (h) | 24 |
| Airway control | NR |
| Cuff pressure (cm H₂O) | 34 |
| Intubation attempts allowed | NR |
| Age (mean ± SD, yr) | 43.44 ± 12.51 |
| BMI (mean ± SD) | NR |
| Duration of anaesthetic (mean ± SD) | 106.42 ± 38.17 |
| Height (cm mean ± SD) | NR |
| Weight (kg mean ± SD) | 56.45 ± 9.77 |

Choi 2023 – Effect of dexmedetomidine and remifentanil infusion on postoperative sore throat after lumbar spine surgery in the prone position

| **Characteristic** | **Value** |
| --- | --- |
| Country | Korea (Republic) |
| Total sample size (N) | 98 |
| Female (N) | 47 |
| Smoking status (N) | 13 |
| Length of follow-up (h) | 24 |
| Airway control | ETT |
| Cuff pressure (cm H₂O) | 20–24 |
| Intubation attempts allowed | ≤ 1 |
| Age (mean ± SD, yr) | 53.50 ± 11.75 |
| BMI (mean ± SD) | NR |
| Duration of anaesthetic (mean ± SD) | 206.65 ± 77.06 |
| Height (cm mean ± SD) | 160.00 ± 9.95 |
| Weight (kg mean ± SD) | 65.30 ± 12.45 |

Choon-KyuCho 2016 – The effect of combining lidocaine with dexamethasone for attenuating postoperative sore throat, cough, and hoarseness

| **Characteristic** | **Value** |
| --- | --- |
| Country | Korea (Republic) |
| Total sample size (N) | 72 |
| Female (N) | 70 |
| Smoking status (N) | NR |
| Length of follow-up (h) | 24 |
| Airway control | ETT |
| Cuff pressure (cm H₂O) | 20 |
| Intubation attempts allowed | ≤ 1 |
| Age (mean ± SD, yr) | 43.60 ± 8.53 |
| BMI (mean ± SD) | NR |
| Duration of anaesthetic (mean ± SD) | 54.80 ± 14.99 |
| Height (cm mean ± SD) | 158.40 ± 5.29 |
| Weight (kg mean ± SD) | 57.50 ± 8.85 |

Chung 2021 – Comparison of prophylactic effect of topical Alchemilla vulgaris in glycerine versus that of dexamethasone on postoperative sore throat after tracheal intubation using a double-lumen endobronchial tube: a randomized controlled study

| **Characteristic** | **Value** |
| --- | --- |
| Country | Korea (Republic) |
| Total sample size (N) | 94 |
| Female (N) | 28 |
| Smoking status (N) | 27 |
| Length of follow-up (h) | 24 |
| Airway control | DLT |
| Cuff pressure (cm H₂O) | 15–25 |
| Intubation attempts allowed | ≤ 3 |
| Age (mean ± SD, yr) | 38.98 ± 17.71 |
| BMI (mean ± SD) | 23.25 ± 4.43 |
| Duration of anaesthetic (mean ± SD) | 135.19 ± 68.21 |
| Height (cm mean ± SD) | 169.02 ± 9.79 |
| Weight (kg mean ± SD) | 66.07 ± 12.18 |

Combes 2007 – Comparison of two induction regimens using or not using muscle relaxant: impact on postoperative upper airway discomfort

| **Characteristic** | **Value** |
| --- | --- |
| Country | France |
| Total sample size (N) | 300 |
| Female (N) | 142 |
| Smoking status (N) | NR |
| Length of follow-up (h) | 24 |
| Airway control | ETT |
| Cuff pressure (cm H₂O) | 20–30 |
| Intubation attempts allowed | NR |
| Age (mean ± SD, yr) | 42.50 ± 9.47 |
| BMI (mean ± SD) | NR |
| Duration of anaesthetic (mean ± SD) | 105.00 ± 49.47 |
| Height (cm mean ± SD) | 168.49 ± 16.08 |
| Weight (kg mean ± SD) | 71.49 ± 13.06 |

Devi 2022 – Comparison of Preoperative Magnesium Sulphate and Budesonide Nebulisation in Reducing the Incidence and Severity of Postoperative Sore Throat: A Randomised Controlled Study

| **Characteristic** | **Value** |
| --- | --- |
| Country | India |
| Total sample size (N) | 120 |
| Female (N) | 51 |
| Smoking status (N) | NR |
| Length of follow-up (h) | 48 |
| Airway control | ETT |
| Cuff pressure (cm H₂O) | 20–25 |
| Intubation attempts allowed | ≤ 1 |
| Age (mean ± SD, yr) | 40.63 ± 12.16 |
| BMI (mean ± SD) | 25.63 ± 5.02 |
| Duration of anaesthetic (mean ± SD) | 68.40 ± 17.87 |
| Height (cm mean ± SD) | NR |
| Weight (kg mean ± SD) | NR |

Eidi 2014 – Comparing the effect of dexamethasone before and after tracheal intubation on sore throat after tympanoplasty surgery: A randomized controlled trial

| **Characteristic** | **Value** |
| --- | --- |
| Country | Iran |
| Total sample size (N) | 70 |
| Female (N) | 36 |
| Smoking status (N) | NR |
| Length of follow-up (h) | 24 |
| Airway control | ETT |
| Cuff pressure (cm H₂O) | 10-20 |
| Intubation attempts allowed | NR |
| Age (mean ± SD, yr) | 40.40 ± 7.77 |
| BMI (mean ± SD) | NR |
| Duration of anaesthetic (mean ± SD) | 115.10 ± 47.44 |
| Height (cm mean ± SD) | NR |
| Weight (kg mean ± SD) | 71.25 ± 8.14 |

Estebe 2004 – Alkalinization of intra-cuff lidocaine and use of gel lubrication protect against tracheal tube-induced emergence phenomena

| **Characteristic** | **Value** |
| --- | --- |
| Country | France |
| Total sample size (N) | 60 |
| Female (N) | 21 |
| Smoking status (N) | NR |
| Length of follow-up (h) | 24 |
| Airway control | ETT |
| Cuff pressure (cm H₂O) | 30 |
| Intubation attempts allowed | ≤ 1 |
| Age (mean ± SD, yr) | 49.08 ± 7.64 |
| BMI (mean ± SD) | NR |
| Duration of anaesthetic (mean ± SD) | 188.00 ± 55.37 |
| Height (cm mean ± SD) | 169.67 ± 9.12 |
| Weight (kg mean ± SD) | 77.33 ± 14.48 |

Faiz 2014 – Comparing the effect of ketamine and benzydamine gargling with placebo on post-operative sore throat: A randomized controlled trial

| **Characteristic** | **Value** |
| --- | --- |
| Country | Iran |
| Total sample size (N) | 60 |
| Female (N) | 60 |
| Smoking status (N) | NR |
| Length of follow-up (h) | 24 |
| Airway control | ETT |
| Cuff pressure (cm H₂O) | 20 |
| Intubation attempts allowed | NR |
| Age (mean ± SD, yr) | 35.43 ± 11.68 |
| BMI (mean ± SD) | NR |
| Duration of anaesthetic (mean ± SD) | NR |
| Height (cm mean ± SD) | 162.43 ± 5.996 |
| Weight (kg mean ± SD) | 65.50 ± 10.06 |

Furqan 2016 – Effect of applying lignocaine gel, diclofenac gel or their combination on endotracheal tube on the hemodynamic response and incidence of postoperative complications in patients undergoing CABG surgery

| **Characteristic** | **Value** |
| --- | --- |
| Country | Pakistan |
| Total sample size (N) | 150 |
| Female (N) | 21 |
| Smoking status (N) | NR |
| Length of follow-up (h) | 6 |
| Airway control | ETT |
| Cuff pressure (cm H₂O) | ≤ 27 |
| Intubation attempts allowed | ≤ 1 |
| Age (mean ± SD, yr) | 54.87 ± 9.31 |
| BMI (mean ± SD) | NR |
| Duration of anaesthetic (mean ± SD) | NR |
| Height (cm mean ± SD) | NR |
| Weight (kg mean ± SD) | NR |

Gaur 2017 – Efficacy and Safety of Using Air Versus Alkalinized 2% Lignocaine for Inflating Endotracheal Tube Cuff and Its Pressure Effects on Incidence of Postoperative Coughing and Sore Throat

| **Characteristic** | **Value** |
| --- | --- |
| Country | India |
| Total sample size (N) | 100 |
| Female (N) | 49 |
| Smoking status (N) | NR |
| Length of follow-up (h) | 24 |
| Airway control | ETT |
| Cuff pressure (cm H₂O) | As per arm |
| Intubation attempts allowed | NR |
| Age (mean ± SD, yr) | 44.62 ± 15.10 |
| BMI (mean ± SD) | NR |
| Duration of anaesthetic (mean ± SD) | 159.90 ± 42.39 |
| Height (cm mean ± SD) | NR |
| Weight (kg mean ± SD) | NR |

Gulhas 2007 – Dexpanthenol pastille and benzydamine hydrochloride spray for the prevention of post-operative sore throat

| **Characteristic** | **Value** |
| --- | --- |
| Country | Turkey |
| Total sample size (N) | 180 |
| Female (N) | 134 |
| Smoking status (N) | 45 |
| Length of follow-up (h) | 24 |
| Airway control | ETT |
| Cuff pressure (cm H₂O) | 27–34 |
| Intubation attempts allowed | NR |
| Age (mean ± SD, yr) | 36.07 ± 12.11 |
| BMI (mean ± SD) | NR |
| Duration of anaesthetic (mean ± SD) | 114.20 ± 70.59 |
| Height (cm mean ± SD) | 162.67 ± 8.25 |
| Weight (kg mean ± SD) | 66.47 ± 13.17 |

Gupta 2014 – Evaluation of preoperative Strepsils lozenges on incidence of postextubation cough and sore throat in smokers undergoing anesthesia with endotracheal intubation

| **Characteristic** | **Value** |
| --- | --- |
| Country | India |
| Total sample size (N) | 100 |
| Female (N) | 7 |
| Smoking status (N) | NR |
| Length of follow-up (h) | 24 |
| Airway control | ETT |
| Cuff pressure (cm H₂O) | ≤ 34 |
| Intubation attempts allowed | ≤ 2 |
| Age (mean ± SD, yr) | 41.01 ± 14.03 |
| BMI (mean ± SD) | NR |
| Duration of anaesthetic (mean ± SD) | 156.45 ± 68.39 |
| Height (cm mean ± SD) | NR |
| Weight (kg mean ± SD) | 61.47 ± 9.69 |

Gupta 2022 – The comparison of inflation of cuff with 1% propofol, 4% lignocaine, or 0.9% saline on laryngotracheal morbidity

| **Characteristic** | **Value** |
| --- | --- |
| Country | India |
| Total sample size (N) | 120 |
| Female (N) | 44 |
| Smoking status (N) | NR |
| Length of follow-up (h) | 24 |
| Airway control | ETT |
| Cuff pressure (cm H₂O) | ≤ 27 |
| Intubation attempts allowed | ≤ 1 |
| Age (mean ± SD, yr) | 46.06 ± 15.84 |
| BMI (mean ± SD) | 23.80 ± 2.50 |
| Duration of anaesthetic (mean ± SD) | 116.00 ± 40.66 |
| Height (cm mean ± SD) | 170.25 ± 11.25 |
| Weight (kg mean ± SD) | 69.13 ± 12.28 |

Harshitha 2024 – Effect of intracuff instillation of alkalinized 2% lignocaine on post-operative sore throat under general anaesthesia

| **Characteristic** | **Value** |
| --- | --- |
| Country | India |
| Total sample size (N) | 120 |
| Female (N) | 68 |
| Smoking status (N) | 0 |
| Length of follow-up (h) | 24 |
| Airway control | ETT |
| Cuff pressure (cm H₂O) | ≤ 25 |
| Intubation attempts allowed | ≤ 1 |
| Age (mean ± SD, yr) | 34.90 ± 8.89 |
| BMI (mean ± SD) | 25.30 ± 3.41 |
| Duration of anaesthetic (mean ± SD) | 82.00 ± 27.16 |
| Height (cm mean ± SD) | NR |
| Weight (kg mean ± SD) | NR |

Huang 2010 – The effectiveness of benzydamine hydrochloride spraying on the endotracheal tube cuff or oral mucosa for postoperative sore throat

| **Characteristic** | **Value** |
| --- | --- |
| Country | Taiwan |
| Total sample size (N) | 380 |
| Female (N) | 196 |
| Smoking status (N) | 155 |
| Length of follow-up (h) | 24 |
| Airway control | ETT |
| Cuff pressure (cm H₂O) | 20–25 |
| Intubation attempts allowed | ≤ 1 |
| Age (mean ± SD, yr) | 47.63 ± 16.80 |
| BMI (mean ± SD) | 23.60 ± 3.60 |
| Duration of anaesthetic (mean ± SD) | 177.99 ± 89.25 |
| Height (cm mean ± SD) | 162.78 ± 8.96 |
| Weight (kg mean ± SD) | 62.70 ± 11.50 |

Ibrahim 2016 – Licorice versus ketamine gargle for postoperative sore throat due to insertion of a double-lumen endobronchial tube

| **Characteristic** | **Value** |
| --- | --- |
| Country | Egypt |
| Total sample size (N) | 90 |
| Female (N) | 21 |
| Smoking status (N) | NR |
| Length of follow-up (h) | 24 |
| Airway control | DLT |
| Cuff pressure (cm H₂O) | ≤ 27 |
| Intubation attempts allowed | ≤ 1 |
| Age (mean ± SD, yr) | 46.47 ± 3.98 |
| BMI (mean ± SD) | NR |
| Duration of anaesthetic (mean ± SD) | 195.80 ± 17.36 |
| Height (cm mean ± SD) | NR |
| Weight (kg mean ± SD) | 70.07 ± 4.87 |

Jain 2023 – The effect of lidocaine jelly on a taper-shaped cuff of an endotracheal tube on postoperative sore throat: a prospective randomized study in Shyam Shah Medical College Rewa

| **Characteristic** | **Value** |
| --- | --- |
| Country | India |
| Total sample size (N) | 210 |
| Female (N) | 133 |
| Smoking status (N) | NR |
| Length of follow-up (h) | 24 |
| Airway control | ETT |
| Cuff pressure (cm H₂O) | 27 |
| Attempts allowed | ≤ 1 |
| Age (mean ± SD, yr) | 57.0 ± 15.50 |
| BMI (mean ± SD) | 24.7 ± 3.75 |
| Duration of anaesthetic (min ± SD) | 65.0 ± 42.65 |
| Height (cm mean ± SD) | 160.5 ± 8.51 |
| Weight (kg mean ± SD) | 64.0 ± 11.02 |

Kajal 2019 – Comparison of three different methods of attenuating postoperative sore throat, cough, and hoarseness of voice in patients undergoing tracheal intubation

| **Characteristic** | **Value** |
| --- | --- |
| Country | India |
| Total sample size (N) | 120 |
| Female (N) | 55 |
| Smoking status (N) | NR |
| Length of follow-up (h) | 24 |
| Airway control | ETT |
| Cuff pressure (cm H₂O) | 18–22 |
| Intubation attempts allowed | ≤ 1 |
| Age (mean ± SD, yr) | 39.23 ± 13.46 |
| BMI (mean ± SD) | NR |
| Duration of anaesthetic (mean ± SD) | 140.07 ± 52.95 |
| Height (cm mean ± SD) | NR |
| Weight (kg mean ± SD) | 58.07 ± 10.46 |

Kamel 2020 – The effect of preoperative nebulized magnesium sulfate versus lidocaine on the prevention of post-intubation sore throat

| **Characteristic** | **Value** |
| --- | --- |
| Country | Egypt |
| Total sample size (N) | 78 |
| Female (N) | 35 |
| Smoking status (N) | 63.8 |
| Length of follow-up (h) | 24 |
| Airway control | ETT |
| Cuff pressure (cm H₂O) | 20–22 |
| Intubation attempts allowed | NR |
| Age (mean ± SD, yr) | 31.80 ± 7.22 |
| BMI (mean ± SD) | 29.17 ± 2.44 |
| Duration of anaesthetic (mean ± SD) | 120.53 ± 118.96 |
| Height (cm mean ± SD) | NR |
| Weight (kg mean ± SD) | NR |

Kang 2015 – Preventive effect of ketamine gargling for postoperative sore throat after endotracheal intubation

| **Characteristic** | **Value** |
| --- | --- |
| Country | Korea (Republic of) |
| Total sample size (N) | 40 |
| Female (N) | 22 |
| Smoking status (N) | NR |
| Length of follow-up (h) | 24 |
| Airway control | ETT |
| Cuff pressure (cm H₂O) | 15–20 |
| Intubation attempts allowed | ≤ 1 |
| Age (mean ± SD, yr) | 42.40 ± 11.65 |
| BMI (mean ± SD) | NR |
| Duration of anaesthetic (mean ± SD) | 100.55 ± 11.14 |
| Height (cm mean ± SD) | 165.85 ± 7.06 |
| Weight (kg mean ± SD) | 61.20 ± 8.36 |

Kayina 2018 – Postintubation sequels: influence of fluticasone and technique of intra-operative muscle relaxation

| **Characteristic** | **Value** |
| --- | --- |
| Country | India |
| Total sample size (N) | 120 |
| Female (N) | 72 |
| Smoking status (N) | 0 |
| Length of follow-up (h) | 24 |
| Airway control | ETT |
| Cuff pressure (cm H₂O) | ≤ 25 |
| Intubation attempts allowed | NR |
| Age (mean ± SD, yr) | 34.83 ± 11.61 |
| BMI (mean ± SD) | NR |
| Duration of anaesthetic (mean ± SD) | NR |
| Height (cm mean ± SD) | NR |
| Weight (kg mean ± SD) | NR |

Kheirabadi 2021 – Comparison prophylactic effects of gargling different doses of ketamine on attenuating postoperative sore throat: a single-blind randomized controlled trial

| **Characteristic** | **Value** |
| --- | --- |
| Country | Iran (Islamic Republic of) |
| Total sample size (N) | 96 |
| Female (N) | 33 |
| Smoking status (N) | NR |
| Length of follow-up (h) | 24 |
| Airway control | ETT |
| Cuff pressure (cm H₂O) | 18–22 |
| Intubation attempts allowed | ≤ 1 |
| Age (mean ± SD, yr) | 30.97 ± 12.64 |
| BMI (mean ± SD) | NR |
| Duration of anaesthetic (mean ± SD) | 96.54 ± 38.85 |
| Height (cm mean ± SD) | NR |
| Weight (kg mean ± SD) | 68.86 ± 11.99 |

Ki 2020 – Effect of dexamethasone gargle, intravenous dexamethasone, and their combination on postoperative sore throat: a randomized controlled trial

| **Characteristic** | **Value** |
| --- | --- |
| Country | Korea (Republic of) |
| Total sample size (N) | 96 |
| Female (N) | 58 |
| Smoking status (N) | NR |
| Length of follow-up (h) | 24 |
| Airway control | ETT |
| Cuff pressure (cm H₂O) | 20–30 |
| Intubation attempts allowed | ≤ 3 |
| Age (mean ± SD, yr) | 47.57 ± 13.31 |
| BMI (mean ± SD) | NR |
| Duration of anaesthetic (mean ± SD) | 52.57 ± 11.33 |
| Height (cm mean ± SD) | 163.40 ± 8.62 |
| Weight (kg mean ± SD) | 66.13 ± 11.69 |

Kim 2018 – Tracheal tubes lubricated with water to reduce sore throat after intubation: a randomized non-inferiority trial

| **Characteristic** | **Value** |
| --- | --- |
| Country | Korea (Republic of) |
| Total sample size (N) | 296 |
| Female (N) | 150 |
| Smoking status (N) | NR |
| Length of follow-up (h) | 24 |
| Airway control | ETT |
| Cuff pressure (cm H₂O) | ≤ 25 |
| Intubation attempts allowed | ≤ 2 |
| Age (mean ± SD, yr) | 53.95 ± 14.14 |
| BMI (mean ± SD) | 24.55 ± 3.89 |
| Duration of anaesthetic (mean ± SD) | 154.06 ± 84.11 |
| Height (cm mean ± SD) | 162.65 ± 8.42 |
| Weight (kg mean ± SD) | 65.20 ± 12.08 |

Klemola 1988 – Post-operative sore throat: effect of lignocaine jelly and spray with endotracheal intubation

| **Characteristic** | **Value** |
| --- | --- |
| Country | Finland |
| Total sample size (N) | 114 |
| Female (N) | 60 |
| Smoking status (N) | NR |
| Length of follow-up (h) | 24 |
| Airway control | ETT |
| Cuff pressure (cm H₂O) | ≤ 25 |
| Intubation attempts allowed | NR |
| Age (mean ± SD, yr) | 39.59 ± 12.44 |
| BMI (mean ± SD) | NR |
| Duration of anaesthetic (mean ± SD) | 158.26 ± 71.07 |
| Height (cm mean ± SD) | 168.95 ± 9.63 |
| Weight (kg mean ± SD) | 67.27 ± 14.11 |

Kumar 2019 – An evaluation of the efficacy of ketamine gargle and benzydamine hydrochloride gargle for attenuating post operative sore throat: a prospective randomized, placebo controlled single-blind study

| **Characteristic** | **Value** |
| --- | --- |
| Country | India |
| Total sample size (N) | 90 |
| Female (N) | 37 |
| Smoking status (N) | NR |
| Length of follow-up (h) | 24 |
| Airway control | ETT |
| Cuff pressure (cm H₂O) | 18–22 |
| Intubation attempts allowed | ≤ 2 |
| Age (mean ± SD, yr) | 39.64 |
| BMI (mean ± SD) | NR |
| Duration of anaesthetic (mean ± SD) | 144.60 |
| Height (cm mean ± SD) | 161.44 |
| Weight (kg mean ± SD) | 58.65 |

Kumar 2021 – Effect of preoperative nebulised dexamethasone and nebulised magnesium sulphate on postoperative sore throat in prone position surgeries: a randomised double-blind study

| **Characteristic** | **Value** |
| --- | --- |
| Country | India |
| Total sample size (N) | 80 |
| Female (N) | 36 |
| Smoking status (N) | 0 |
| Length of follow-up (h) | 24 |
| Airway control | ETT |
| Cuff pressure (cm H₂O) | ≤ 20 |
| Intubation attempts allowed | ≤ 1 |
| Age (mean ± SD, yr) | 37.63 ± 8.21 |
| BMI (mean ± SD) | NR |
| Duration of anaesthetic (mean ± SD) | 109.07 ± 16.15 |
| Height (cm mean ± SD) | 165.39 ± 7.14 |
| Weight (kg mean ± SD) | 61.29 ± 9.24 |

Kusumaphanyo 2021 – The efficacy of single-dose aescin in preventing postoperative sore throat compared to placebos: a double-blinded, randomized controlled trial

| **Characteristic** | **Value** |
| --- | --- |
| Country | Thailand |
| Total sample size (N) | 147 |
| Female (N) | 101 |
| Smoking status (N) | NR |
| Length of follow-up (h) | 24 |
| Airway control | ETT |
| Cuff pressure (cm H₂O) | 20–30 |
| Intubation attempts allowed | ≤ 1 |
| Age (mean ± SD, yr) | 44.24 ± 11.08 |
| BMI (mean ± SD) | NR |
| Duration of anaesthetic (mean ± SD) | 136.36 ± 21.40 |
| Height (cm mean ± SD) | 159.35 ± 7.36 |
| Weight (kg mean ± SD) | 63.15 ± 12.31 |

Lee 2016 – The prophylactic effect of dexamethasone on postoperative sore throat in prone position surgery

| **Characteristic** | **Value** |
| --- | --- |
| Country | Korea (Republic of) |
| Total sample size (N) | 150 |
| Female (N) | 89 |
| Smoking status (N) | NR |
| Length of follow-up (h) | 24 |
| Airway control | ETT |
| Cuff pressure (cm H₂O) | 10-20 |
| Intubation attempts allowed | ≤ 1 |
| Age (mean ± SD, yr) | 62.42 ± 13.02 |
| BMI (mean ± SD) | NR |
| Duration of anaesthetic (mean ± SD) | 161.41 ± 42.97 |
| Height (cm mean ± SD) | 161.05 ± 9.58 |
| Weight (kg mean ± SD) | 63.27 ± 11.19 |

Lee 2017 – Combined intraoperative paracetamol and preoperative dexamethasone reduces postoperative sore throat: a prospective randomized study

| **Characteristic** | **Value** |
| --- | --- |
| Country | Korea (Republic of) |
| Total sample size (N) | 232 |
| Female (N) | 110 |
| Smoking status (N) | NR |
| Length of follow-up (h) | 24 |
| Airway control | ETT |
| Cuff pressure (cm H₂O) | ≤ 27 |
| Intubation attempts allowed | ≤ 1 |
| Age (mean ± SD, yr) | 54.50 ± 15.55 |
| BMI (mean ± SD) | 23.65 ± 3.29 |
| Duration of anaesthetic (mean ± SD) | 172.00 ± 92.00 |
| Height (cm mean ± SD) | 164.00 ± 8.55 |
| Weight (kg mean ± SD) | 63.50 ± 11.50 |

Lee 2017 – Effects of topical dexamethasone in postoperative sore throat

| **Characteristic** | **Value** |
| --- | --- |
| Country | Korea (Republic of) |
| Total sample size (N) | 90 |
| Female (N) | 48 |
| Smoking status (N) | NR |
| Length of follow-up (h) | 24 |
| Airway control | ETT |
| Cuff pressure (cm H₂O) | 20–30 |
| Intubation attempts allowed | ≤ 2 |
| Age (mean ± SD, yr) | 47.67 ± 12.21 |
| BMI (mean ± SD) | 25.00 ± 2.68 |
| Duration of anaesthetic (mean ± SD) | 46.67 ± 11.57 |
| Height (cm mean ± SD) | 157.33 ± 6.30 |
| Weight (kg mean ± SD) | 63.67 ± 9.32 |

Lee 2017 – The effect of lidocaine jelly on a taper-shaped cuff of an endotracheal tube on the postoperative sore throat: a prospective randomized study

| **Characteristic** | **Value** |
| --- | --- |
| Country | Korea (Republic of) |
| Total sample size (N) | 210 |
| Female (N) | 133 |
| Smoking status (N) | NR |
| Length of follow-up (h) | 24 |
| Airway control | ETT |
| Cuff pressure (cm H₂O) | ≤ 27 |
| Intubation attempts allowed | ≤ 1 |
| Age (mean ± SD, yr) | 57.00 ± 15.50 |
| BMI (mean ± SD) | 24.70 ± 3.75 |
| Duration of anaesthetic (mean ± SD) | 65.00 ± 42.65 |
| Height (cm mean ± SD) | 160.50 ± 8.51 |
| Weight (kg mean ± SD) | 64.00 ± 11.02 |

Lin 2024 – Effect of intravenous and topical laryngeal lidocaine on sore throat after extubation: a prospective randomized controlled study

| **Characteristic** | **Value** |
| --- | --- |
| Country | China |
| Total sample size (N) | 144 |
| Female (N) | 91 |
| Smoking status (N) | NR |
| Length of follow-up (h) | 24 |
| Airway control | ETT |
| Cuff pressure (cm H₂O) | ≤ 25 |
| Intubation attempts allowed | ≤ 2 |
| Age (mean ± SD, yr) | 43.61 ± 12.29 |
| BMI (mean ± SD) | 23.86 ± 2.74 |
| Duration of anaesthetic (mean ± SD) | 80.40 ± 23.33 |
| Height (cm mean ± SD) | NR |
| Weight (kg mean ± SD) | NR |

Luo 2023 – Effects of ultrasound-guided stellate ganglion block on postoperative sore throat and postoperative sleep disturbance after lumbar spine surgery: a randomized controlled trial

| **Characteristic** | **Value** |
| --- | --- |
| Country | China |
| Total sample size (N) | 60 |
| Female (N) | 23 |
| Smoking status (N) | NR |
| Length of follow-up (h) | 48 |
| Airway control | ETT |
| Cuff pressure (cm H₂O) | 20–30 |
| Intubation attempts allowed | ≤ 1 |
| Age (mean ± SD, yr) | 52.17 ± 9.25 |
| BMI (mean ± SD) | NR |
| Duration of anaesthetic (mean ± SD) | 140.32 ± 47.13 |
| Height (cm mean ± SD) | 162.85 ± 7.63 |
| Weight (kg mean ± SD) | 66.14 ± 9.71 |

Malhotra 2007 – Tracheal morbidity following tracheal intubation: comparison of air, saline and lignocaine used for inflating cuff

| **Characteristic** | **Value** |
| --- | --- |
| Country | India |
| Total sample size (N) | 75 |
| Female (N) | 26 |
| Smoking status (N) | 0 |
| Length of follow-up (h) | 24 |
| Airway control | ETT |
| Cuff pressure (cm H₂O) | 25–30 |
| Intubation attempts allowed | ≤ 1 |
| Age (mean ± SD, yr) | 43.42 ± 12.64 |
| BMI (mean ± SD) | NR |
| Duration of anaesthetic (mean ± SD) | 109.24 ± 34.37 |
| Height (cm mean ± SD) | NR |
| Weight (kg mean ± SD) | 56.79 ± 8.26 |

Mencke 2013 – Anesthesia with propofol versus sevoflurane: does the longer neuromuscular block under sevoflurane anesthesia reduce laryngeal injuries?

| **Characteristic** | **Value** |
| --- | --- |
| Country | Germany |
| Total sample size (N) | 59 |
| Female (N) | 26 |
| Smoking status (N) | 22 |
| Length of follow-up (h) | 72 |
| Airway control | ETT |
| Cuff pressure (cm H₂O) | ≤ 34 |
| Intubation attempts allowed | NR |
| Age (mean ± SD, yr) | 48.00 ± 15.92 |
| BMI (mean ± SD) | 25.95 ± 3.68 |
| Duration of anaesthetic (mean ± SD) | 98.00 ± 34.94 |
| Height (cm mean ± SD) | 172.85 ± 9.30 |
| Weight (kg mean ± SD) | 78.10 ± 15.73 |

Muderris 2019 – Oral flurbiprofen spray for postoperative sore throat and hoarseness: a prospective, randomized, double-blind, placebo-controlled study

| **Characteristic** | **Value** |
| --- | --- |
| Country | Turkey |
| Total sample size (N) | 150 |
| Female (N) | 65 |
| Smoking status (N) | 35 |
| Length of follow-up (h) | 24 |
| Airway control | ETT |
| Cuff pressure (cm H₂O) | 18–22 |
| Intubation attempts allowed | ≤ 1 |
| Age (mean ± SD, yr) | 37.21 ± 2.69 |
| BMI (mean ± SD) | 27.63 ± 2.51 |
| Duration of anaesthetic (mean ± SD) | 151.93 ± 18.61 |
| Height (cm mean ± SD) | NR |
| Weight (kg mean ± SD) | 73.90 ± 3.26 |

Murugaiyan 2023 – Effect of 5% EMLA cream on postoperative sore throat in adults following general endotracheal anesthesia: a randomized placebo-controlled study

| **Characteristic** | **Value** |
| --- | --- |
| Country | India |
| Total sample size (N) | 206 |
| Female (N) | 95 |
| Smoking status (N) | NR |
| Length of follow-up (h) | 24 |
| Airway control | ETT |
| Cuff pressure (cm H₂O) | 20–30 |
| Intubation attempts allowed | ≤ 2 |
| Age (mean ± SD, yr) | 42.80 ± 11.34 |
| BMI (mean ± SD) | NR |
| Duration of anaesthetic (mean ± SD) | 115.90 ± 30.39 |
| Height (cm mean ± SD) | NR |
| Weight (kg mean ± SD) | NR |

Narimani 2016 – The effect of betamethasone gel and lidocaine jelly applied over tracheal tube cuff on postoperative sore throat, cough, and hoarseness

| **Characteristic** | **Value** |
| --- | --- |
| Country | Iran (Islamic Republic of) |
| Total sample size (N) | 99 |
| Female (N) | 60 |
| Smoking status (N) | NR |
| Length of follow-up (h) | 24 |
| Airway control | ETT |
| Cuff pressure (cm H₂O) | 25–30 |
| Intubation attempts allowed | ≤ 2 |
| Age (mean ± SD, yr) | 33.30 ± 19.86 |
| BMI (mean ± SD) | NR |
| Duration of anaesthetic (mean ± SD) | 33.23 ± 19.85 |
| Height (cm mean ± SD) | NR |
| Weight (kg mean ± SD) | NR |

Navarro 2007 – Effectiveness and safety of endotracheal tube cuffs filled with air versus filled with alkalinized lidocaine: a randomized clinical trial

| **Characteristic** | **Value** |
| --- | --- |
| Country | Brazil |
| Total sample size (N) | 50 |
| Female (N) | 50 |
| Smoking status (N) | 0 |
| Length of follow-up (h) | 24 |
| Airway control | ETT |
| Cuff pressure (cm H₂O) | ≤ 20 |
| Intubation attempts allowed | ≤ 1 |
| Age (mean ± SD, yr) | 45.15 ± 9.95 |
| BMI (mean ± SD) | NR |
| Duration of anaesthetic (mean ± SD) | 219.10 ± 67.80 |
| Height (cm mean ± SD) | 157.85 ± 6.48 |
| Weight (kg mean ± SD) | 67.35 ± 11.74 |

Navarro 2012 – The effect of intracuff alkalinized 2% lidocaine on emergence coughing, sore throat, and hoarseness in smokers

| **Characteristic** | **Value** |
| --- | --- |
| Country | Brazil |
| Total sample size (N) | 50 |
| Female (N) | 37 |
| Smoking status (N) | 50 |
| Length of follow-up (h) | 24 |
| Airway control | ETT |
| Cuff pressure (cm H₂O) | as per trial arm |
| Intubation attempts allowed | ≤ 1 |
| Age (mean ± SD, yr) | 68.30 ± 13.48 |
| BMI (mean ± SD) | NR |
| Duration of anaesthetic (mean ± SD) | 215.10 ± 82.79 |
| Height (cm mean ± SD) | 163.25 ± 9.17 |
| Weight (kg mean ± SD) | 68.30 ± 13.48 |

Niu 2022 – Effect of intratracheal dexmedetomidine combined with ropivacaine on postoperative sore throat: a prospective randomised double-blinded controlled trial

| **Characteristic** | **Value** |
| --- | --- |
| Country | China |
| Total sample size (N) | 200 |
| Female (N) | 114 |
| Smoking status (N) | NR |
| Length of follow-up (h) | 24 |
| Airway control | ETT |
| Cuff pressure (cm H₂O) | 27–34 |
| Intubation attempts allowed | ≤ 1 |
| Age (mean ± SD, yr) | 49.83 ± 10.14 |
| BMI (mean ± SD) | 23.90 ± 2.55 |
| Duration of anaesthetic (mean ± SD) | 182.85 ± 34.86 |
| Height (cm mean ± SD) | 164.45 ± 7.51 |
| Weight (kg mean ± SD) | 64.83 ± 9.92 |

Ogata 2005 – Gargling with sodium azulene sulfonate reduces the postoperative sore throat after intubation of the trachea

| **Characteristic** | **Value** |
| --- | --- |
| Country | Japan |
| Total sample size (N) | 40 |
| Female (N) | 20 |
| Smoking status (N) | NR |
| Length of follow-up (h) | 24 |
| Airway control | ETT |
| Cuff pressure (cm H₂O) | ≤ 20 |
| Intubation attempts allowed | ≤ 2 |
| Age (mean ± SD, yr) | 59.05 ± 12.64 |
| BMI (mean ± SD) | NR |
| Duration of anaesthetic (mean ± SD) | 211.05 ± 78.70 |
| Height (cm mean ± SD) | 157.30 ± 8.41 |
| Weight (kg mean ± SD) | 55.60 ± 9.43 |

OlgunKeles 2024 – The effect of topical benzydamine hydrochloride and cuff pressure monitorization on postoperative sore throat due to intubation

| **Characteristic** | **Value** |
| --- | --- |
| Country | Turkey |
| Total sample size (N) | 210 |
| Female (N) | 119 |
| Smoking status (N) | NR |
| Length of follow-up (h) | 24 |
| Airway control | ETT |
| Cuff pressure (cm H₂O) | 22–26 |
| Intubation attempts allowed | ≤ 1 |
| Age (mean ± SD, yr) | 46.30 ± 11.78 |
| BMI (mean ± SD) | NR |
| Duration of anaesthetic (mean ± SD) | 136.30 ± 62.54 |
| Height (cm mean ± SD) | NR |
| Weight (kg mean ± SD) | 72.07 ± 13.73 |

Orji 2020 – Nebulized magnesium versus ketamine for prevention of post-operative sore throat in patients for general anaesthesia

| **Characteristic** | **Value** |
| --- | --- |
| Country | Nigeria |
| Total sample size (N) | 105 |
| Female (N) | 52 |
| Smoking status (N) | NR |
| Length of follow-up (h) | 24 |
| Airway control | ETT |
| Cuff pressure (cm H₂O) | ≤ 25 |
| Intubation attempts allowed | ≤ 1 |
| Age (mean ± SD, yr) | 43.77 ± 12.91 |
| BMI (mean ± SD) | NR |
| Duration of anaesthetic (mean ± SD) | 157.77 ± 41.43 |
| Height (cm mean ± SD) | NR |
| Weight (kg mean ± SD) | 68.93 ± 9.61 |

Ozaki 2001 – Transdermal ketoprofen mitigates the severity of postoperative sore throat

| **Characteristic** | **Value** |
| --- | --- |
| Country | Japan |
| Total sample size (N) | 63 |
| Female (N) | 46 |
| Smoking status (N) | NR |
| Length of follow-up (h) | 20 |
| Airway control | ETT |
| Cuff pressure (cm H₂O) | ≤ 20 |
| Intubation attempts allowed | ≤ 2 |
| Age (mean ± SD, yr) | 52.49 ± 13.09 |
| BMI (mean ± SD) | NR |
| Duration of anaesthetic (mean ± SD) | 202.59 ± 59.32 |
| Height (cm mean ± SD) | NR |
| Weight (kg mean ± SD) | 57.48 ± 8.08 |

Paramesh 2023 – The effect of preoperative nebulisations with ketamine and magnesium sulfate on incidence of postoperative sore throat

| **Characteristic** | **Value** |
| --- | --- |
| Country | India |
| Total sample size (N) | 120 |
| Female (N) | 73 |
| Smoking status (N) | NR |
| Length of follow-up (h) | 24 |
| Airway control | ETT |
| Cuff pressure (cm H₂O) | ≤ 20 |
| Intubation attempts allowed | ≤ 2 |
| Age (mean ± SD, yr) | 40.07 ± 12.25 |
| BMI (mean ± SD) | NR |
| Duration of anaesthetic (mean ± SD) | NR |
| Height (cm mean ± SD) | NR |
| Weight (kg mean ± SD) | 11.93 ± 2.31 |

Park 2004 – Prevention of postoperative sore throat using capsicum plaster applied at the Korean hand acupuncture point

| **Characteristic** | **Value** |
| --- | --- |
| Country | Korea (Republic of) |
| Total sample size (N) | 154 |
| Female (N) | 150 |
| Smoking status (N) | NR |
| Length of follow-up (h) | 24 |
| Airway control | ETT |
| Cuff pressure (cm H₂O) | ≤ 19 |
| Intubation attempts allowed | ≤ 2 |
| Age (mean ± SD, yr) | 43.88 ± 5.72 |
| BMI (mean ± SD) | NR |
| Duration of anaesthetic (mean ± SD) | 124.63 ± 13.74 |
| Height (cm mean ± SD) | 157.00 ± 6.62 |
| Weight (kg mean ± SD) | 54.27 ± 6.87 |

Park 2008 – Prophylactic dexamethasone decreases the incidence of sore throat and hoarseness after tracheal extubation with a double-lumen endobronchial tube

| **Characteristic** | **Value** |
| --- | --- |
| Country | Korea (Republic of) |
| Total sample size (N) | 168 |
| Female (N) | 56 |
| Smoking status (N) | 47 |
| Length of follow-up (h) | 24 |
| Airway control | DLT |
| Cuff pressure (cm H₂O) | ≤ 20 |
| Intubation attempts allowed | ≤ 3 |
| Age (mean ± SD, yr) | 47.37 ± 19.93 |
| BMI (mean ± SD) | NR |
| Duration of anaesthetic (mean ± SD) | 159.03 ± 65.91 |
| Height (cm mean ± SD) | 166.93 ± 8.24 |
| Weight (kg mean ± SD) | 63.73 ± 11.22 |

Park 2010 – Prophylactic effect of dexamethasone in reducing postoperative sore throat

| **Characteristic** | **Value** |
| --- | --- |
| Country | Korea (Republic of) |
| Total sample size (N) | 70 |
| Female (N) | 47 |
| Smoking status (N) | NR |
| Length of follow-up (h) | 24 |
| Airway control | ETT |
| Cuff pressure (cm H₂O) | ≤ 20 |
| Intubation attempts allowed | ≤ 1 |
| Age (mean ± SD, yr) | 42.09 ± 9.81 |
| BMI (mean ± SD) | NR |
| Duration of anaesthetic (mean ± SD) | 103.19 ± 31.53 |
| Height (cm mean ± SD) | NR |
| Weight (kg mean ± SD) | 62.61 ± 11.77 |

Park 2010 – The effect of intravenous low dose ketamine for reducing postoperative sore throat

| **Characteristic** | **Value** |
| --- | --- |
| Country | Korea (Republic of) |
| Total sample size (N) | 70 |
| Female (N) | 47 |
| Smoking status (N) | 25 |
| Length of follow-up (h) | 24 |
| Airway control | ETT |
| Cuff pressure (cm H₂O) | 10-20 |
| Intubation attempts allowed | ≤ 1 |
| Age (mean ± SD, yr) | 40.92 ± 10.03 |
| BMI (mean ± SD) | NR |
| Duration of anaesthetic (mean ± SD) | 105.10 ± 38.40 |
| Height (cm mean ± SD) | NR |
| Weight (kg mean ± SD) | 63.24 ± 12.84 |

Park 2011 – Application of triamcinolone acetonide paste to the endotracheal tube reduces postoperative sore throat: a randomized controlled trial

| **Characteristic** | **Value** |
| --- | --- |
| Country | Korea (Republic of) |
| Total sample size (N) | 150 |
| Female (N) | 84 |
| Smoking status (N) | NR |
| Length of follow-up (h) | 24 |
| Airway control | ETT |
| Cuff pressure (cm H₂O) | ≤ 20 |
| Intubation attempts allowed | ≤ 1 |
| Age (mean ± SD, yr) | 47.14 ± 9.87 |
| BMI (mean ± SD) | NR |
| Duration of anaesthetic (mean ± SD) | 111.85 ± 41.54 |
| Height (cm mean ± SD) | NR |
| Weight (kg mean ± SD) | 63.69 ± 11.51 |

Park 2015 – A randomized, double-blind, non-inferiority trial of magnesium sulphate versus dexamethasone for prevention of postoperative sore throat after lumbar spinal surgery in the prone position

| **Characteristic** | **Value** |
| --- | --- |
| Country | Korea (Republic of) |
| Total sample size (N) | 146 |
| Female (N) | 63 |
| Smoking status (N) | 27 |
| Length of follow-up (h) | 48 |
| Airway control | ETT |
| Cuff pressure (cm H₂O) | 20–25 |
| Intubation attempts allowed | ≤ 2 |
| Age (mean ± SD, yr) | 51.00 ± 11.96 |
| BMI (mean ± SD) | NR |
| Duration of anaesthetic (mean ± SD) | 187.66 ± 79.22 |
| Height (cm mean ± SD) | 165.50 ± 9.98 |
| Weight (kg mean ± SD) | 67.00 ± 15.84 |

Park 2018 – The influence of high-dose intraoperative remifentanil on postoperative sore throat: a prospective randomized study: a CONSORT-compliant article

| **Characteristic** | **Value** |
| --- | --- |
| Country | Korea (Republic of) |
| Total sample size (N) | 92 |
| Female (N) | 55 |
| Smoking status (N) | NR |
| Length of follow-up (h) | 24 |
| Airway control | ETT |
| Cuff pressure (cm H₂O) | ≤ 27 |
| Intubation attempts allowed | ≤ 1 |
| Age (mean ± SD, yr) | 64.00 ± 10.60 |
| BMI (mean ± SD) | 23.20 ± 3.57 |
| Duration of anaesthetic (mean ± SD) | 172.00 ± 46.84 |
| Height (cm mean ± SD) | 158.50 ± 9.96 |
| Weight (kg mean ± SD) | 58.50 ± 11.94 |

Prajapati 2023 – A hospital-based randomized double-blinded controlled study to assess the efficacy and safety of air versus alkalinized 2% lignocaine for inflating endotracheal tube

| **Characteristic** | **Value** |
| --- | --- |
| Country | India |
| Total sample size (N) | 120 |
| Female (N) | 53 |
| Smoking status (N) | 0 |
| Length of follow-up (h) | 24 |
| Airway control | ETT |
| Cuff pressure (cm H₂O) | ≤ 20 |
| Intubation attempts allowed | ≤ 1 |
| Age (mean ± SD, yr) | 43.25 ± 12.84 |
| BMI (mean ± SD) | 25.95 ± 2.31 |
| Duration of anaesthetic (mean ± SD) | 138.40 ± 11.08 |
| Height (cm mean ± SD) | NR |
| Weight (kg mean ± SD) | NR |

Preethi 2023 – Ketamine nebulization versus magnesium sulphate nebulization for prevention of post operative sore throat in adult patients undergoing general anaesthesia with endotracheal intubation: a comparative study

| **Characteristic** | **Value** |
| --- | --- |
| Country | India |
| Total sample size (N) | 60 |
| Female (N) | 32 |
| Smoking status (N) | NR |
| Length of follow-up (h) | 24 |
| Airway control | ETT |
| Cuff pressure (cm H₂O) | ≤ 20 |
| Intubation attempts allowed | ≤ 2 |
| Age (mean ± SD, yr) | 35.23 ± 10.11 |
| BMI (mean ± SD) | NR |
| Duration of anaesthetic (mean ± SD) | 109.50 ± 36.72 |
| Height (cm mean ± SD) | 163.67 ± 5.99 |
| Weight (kg mean ± SD) | 60.45 ± 9.70 |

Rahimi 2009 – Effects of diclofenac epolamine patch on postoperative sore throat in parturients after cesarean delivery under endotracheal general anesthesia

| **Characteristic** | **Value** |
| --- | --- |
| Country | Iran |
| Total sample size (N) | 220 |
| Female (N) | 220 |
| Smoking status (N) | 0 |
| Length of follow-up (h) | 48 |
| Airway control | ETT |
| Cuff pressure (cm H₂O) | ≤ 20 |
| Intubation attempts allowed | ≤ 1 |
| Age (mean ± SD, yr) | 27.25 ± 5.22 |
| BMI (mean ± SD) | NR |
| Duration of anaesthetic (mean ± SD) | NR |
| Height (cm mean ± SD) | NR |
| Weight (kg mean ± SD) | 68.85 ± 2.91 |

Raikwar 2018 – Comparative study between intravenous dexamethasone versus ketamine gargle versus intravenous dexamethasone combined with ketamine gargle for evaluation of post-operative sore throat and hoarseness in middle ear surgery

| **Characteristic** | **Value** |
| --- | --- |
| Country | India |
| Total sample size (N) | 80 |
| Female (N) | 38 |
| Smoking status (N) | NR |
| Length of follow-up (h) | 24 |
| Airway control | ETT |
| Cuff pressure (cm H₂O) | 18–22 |
| Intubation attempts allowed | ≤ 1 |
| Age (mean ± SD, yr) | 28.61 ± 7.95 |
| BMI (mean ± SD) | NR |
| Duration of anaesthetic (mean ± SD) | 101.15 ± 8.09 |
| Height (cm mean ± SD) | NR |
| Weight (kg mean ± SD) | 49.39 ± 11.08 |

Rajan 2018 – Effect of inhaled budesonide suspension, administered using a metered dose inhaler, on post-operative sore throat, hoarseness of voice and cough

| **Characteristic** | **Value** |
| --- | --- |
| Country | India |
| Total sample size (N) | 46 |
| Female (N) | 29 |
| Smoking status (N) | NR |
| Length of follow-up (h) | 24 |
| Airway control | ETT |
| Cuff pressure (cm H₂O) | 20–22 |
| Intubation attempts allowed | ≤ 2 |
| Age (mean ± SD, yr) | 40.95 ± 11.37 |
| BMI (mean ± SD) | NR |
| Duration of anaesthetic (mean ± SD) | 92.06 ± 20.59 |
| Height (cm mean ± SD) | NR |
| Weight (kg mean ± SD) | 67.00 ± 9.50 |

Reddy 2018 – Dose-dependent effectiveness of ketamine nebulisation in preventing postoperative sore throat due to tracheal intubation

| **Characteristic** | **Value** |
| --- | --- |
| Country | India |
| Total sample size (N) | 90 |
| Female (N) | 50 |
| Smoking status (N) | 0 |
| Length of follow-up (h) | 24 |
| Airway control | ETT |
| Cuff pressure (cm H₂O) | ≤ 20 |
| Intubation attempts allowed | ≤ 2 |
| Age (mean ± SD, yr) | 36.59 ± 9.36 |
| BMI (mean ± SD) | NR |
| Duration of anaesthetic (mean ± SD) | 126.23 ± 31.84 |
| Height (cm mean ± SD) | NR |
| Weight (kg mean ± SD) | 56.02 ± 5.04 |

Rizvanovic 2019 – Effect of intracuff alkalinized 2% lidocaine on endotracheal tube cuff pressure and postoperative throat symptoms in anaesthesia maintained by nitrous oxide

| **Characteristic** | **Value** |
| --- | --- |
| Country | Bosnia and Herzegovina |
| Total sample size (N) | 90 |
| Female (N) | 46 |
| Smoking status (N) | 0 |
| Length of follow-up (h) | 24 |
| Airway control | ETT |
| Cuff pressure (cm H₂O) | As per trial arm |
| Intubation attempts allowed | ≤ 1 |
| Age (mean ± SD, yr) | 49.43 ± 11.29 |
| BMI (mean ± SD) | NR |
| Duration of anaesthetic (mean ± SD) | 88.20 ± 11.94 |
| Height (cm mean ± SD) | NR |
| Weight (kg mean ± SD) | 82.57 ± 11.37 |

Ruetzler 2013 – A randomized, double-blind comparison of licorice versus sugar-water gargle for prevention of postoperative sore throat and postextubation coughing

| **Characteristic** | **Value** |
| --- | --- |
| Country | Austria |
| Total sample size (N) | 236 |
| Female (N) | 95 |
| Smoking status (N) | 138 |
| Length of follow-up (h) | 24 |
| Airway control | DLT |
| Cuff pressure (cm H₂O) | ≤ 27 |
| Intubation attempts allowed | NR |
| Age (mean ± SD, yr) | 57.50 ± 15.48 |
| BMI (mean ± SD) | 26.00 ± 3.99 |
| Duration of anaesthetic (mean ± SD) | NR |
| Height (cm mean ± SD) | NR |
| Weight (kg mean ± SD) | NR |

Safavi 2013 – Intravenous dexamethasone vs. ketamine gargle vs. intravenous dexamethasone combined with ketamine gargle for evaluation of post-operative sore throat and hoarseness: a randomized, placebo-controlled, double-blind clinical trial

| **Characteristic** | **Value** |
| --- | --- |
| Country | Iran |
| Total sample size (N) | 152 |
| Female (N) | 22 |
| Smoking status (N) | NR |
| Length of follow-up (h) | 24 |
| Airway control | ETT |
| Cuff pressure (cm H₂O) | 18–22 |
| Intubation attempts allowed | ≤ 1 |
| Age (mean ± SD, yr) | 32.20 ± 13.18 |
| BMI (mean ± SD) | NR |
| Duration of anaesthetic (mean ± SD) | 80.35 ± 23.67 |
| Height (cm mean ± SD) | NR |
| Weight (kg mean ± SD) | 70.47 ± 8.15 |

Segaran 2018 – Comparison of nebulized ketamine with nebulized magnesium sulfate on the incidence of postoperative sore throat

| **Characteristic** | **Value** |
| --- | --- |
| Country | India |
| Total sample size (N) | 80 |
| Female (N) | 42 |
| Smoking status (N) | NR |
| Length of follow-up (h) | 24 |
| Airway control | ETT |
| Cuff pressure (cm H₂O) | ≤ 20 |
| Intubation attempts allowed | ≤ 2 |
| Age (mean ± SD, yr) | 34.44 ± 11.50 |
| BMI (mean ± SD) | NR |
| Duration of anaesthetic (mean ± SD) | 110.43 ± 25.82 |
| Height (cm mean ± SD) | 163.20 ± 6.05 |
| Weight (kg mean ± SD) | 59.75 ± 10.06 |

Shaaban 2012 – Comparison between betamethasone gel applied over endotracheal tube and ketamine gargle for attenuating postoperative sore throat, cough and hoarseness of voice

| **Characteristic** | **Value** |
| --- | --- |
| Country | Egypt |
| Total sample size (N) | 75 |
| Female (N) | 38 |
| Smoking status (N) | NR |
| Length of follow-up (h) | 24 |
| Airway control | ETT |
| Cuff pressure (cm H₂O) | 18–22 |
| Intubation attempts allowed | ≤ 2 |
| Age (mean ± SD, yr) | 33.27 ± 11.83 |
| BMI (mean ± SD) | NR |
| Duration of anaesthetic (mean ± SD) | 79.67 ± 30.97 |
| Height (cm mean ± SD) | NR |
| Weight (kg mean ± SD) | 58.17 ± 7.02 |

Sharma 2017 – Comparison of magnesium sulfate and normal saline (placebo) nebulization for prevention of postoperative sore throat in patients undergoing lumbar spine surgeries under general anaesthesia with endotracheal intubation in prone position

| **Characteristic** | **Value** |
| --- | --- |
| Country | India |
| Total sample size (N) | 140 |
| Female (N) | 42 |
| Smoking status (N) | NR |
| Length of follow-up (h) | 24 |
| Airway control | ETT |
| Cuff pressure (cm H₂O) | ≤ 20 |
| Intubation attempts allowed | ≤ 1 |
| Age (mean ± SD, yr) | 38.90 ± 12.33 |
| BMI (mean ± SD) | NR |
| Duration of anaesthetic (mean ± SD) | 129.63 ± 9.17 |
| Height (cm mean ± SD) | 166.76 ± 9.18 |
| Weight (kg mean ± SD) | 67.56 ± 7.42 |

Singh 2021 – Comparative study between betamethasone gel and lignocaine jelly applied over the tracheal tube to reduce postoperative airway complications

| **Characteristic** | **Value** |
| --- | --- |
| Country | India |
| Total sample size (N) | 60 |
| Female (N) | 26 |
| Smoking status (N) | NR |
| Length of follow-up (h) | 24 |
| Airway control | ETT |
| Cuff pressure (cm H₂O) | 27–34 |
| Intubation attempts allowed | ≤ 2 |
| Age (mean ± SD, yr) | 36.20 ± 6.28 |
| BMI (mean ± SD) | NR |
| Duration of anaesthetic (mean ± SD) | NR |
| Height (cm mean ± SD) | NR |
| Weight (kg mean ± SD) | 59.20 ± 6.50 |

Sony 2023 – Comparison of normal saline and alkalinized 2% lignocaine to reduce emergence phenomenon and post-intubation morbidities: a prospective, double-blind, randomized study

| **Characteristic** | **Value** |
| --- | --- |
| Country | India |
| Total sample size (N) | 120 |
| Female (N) | NR |
| Smoking status (N) | NR |
| Length of follow-up (h) | 24 |
| Airway control | ETT |
| Cuff pressure (cm H₂O) | ≤ 30 |
| Intubation attempts allowed | NR |
| Age (mean ± SD, yr) | NR |
| BMI (mean ± SD) | NR |
| Duration of anaesthetic (mean ± SD) | NR |
| Height (cm mean ± SD) | NR |
| Weight (kg mean ± SD) | NR |

Sruthi 2024 – Intracuff installation of lignocaine vs normal saline in prevention of post-operative sore throat in patients undergoing surgeries in general anesthesia with endotracheal tube

| **Characteristic** | **Value** |
| --- | --- |
| Country | India |
| Total sample size (N) | 120 |
| Female (N) | NR |
| Smoking status (N) | 0 |
| Length of follow-up (h) | 24 |
| Airway control | ETT |
| Cuff pressure (cm H₂O) | ≤ 30 |
| Intubation attempts allowed | NR |
| Age (mean ± SD, yr) | NR |
| BMI (mean ± SD) | NR |
| Duration of anaesthetic (mean ± SD) | NR |
| Height (cm mean ± SD) | NR |
| Weight (kg mean ± SD) | NR |

Subedi 2019 – Effect of intravenous lidocaine, dexamethasone, and their combination on postoperative sore throat: a randomized controlled trial

| **Characteristic** | **Value** |
| --- | --- |
| Country | Nepal |
| Total sample size (N) | 180 |
| Female (N) | 87 |
| Smoking status (N) | 0 |
| Length of follow-up (h) | 24 |
| Airway control | ETT |
| Cuff pressure (cm H₂O) | 25–30 |
| Intubation attempts allowed | ≤ 1 |
| Age (mean ± SD, yr) | 34.44 ± 12.75 |
| BMI (mean ± SD) | 21.85 ± 3.17 |
| Duration of anaesthetic (mean ± SD) | NR |
| Height (cm mean ± SD) | NR |
| Weight (kg mean ± SD) | NR |

Sujita 2018 – Efficacy of dexamethasone in reducing the incidence of postoperative sore throat: a double-blind randomized study

| **Characteristic** | **Value** |
| --- | --- |
| Country | Nepal |
| Total sample size (N) | 110 |
| Female (N) | 50 |
| Smoking status (N) | NR |
| Length of follow-up (h) | 24 |
| Airway control | ETT |
| Cuff pressure (cm H₂O) | ≤ 30 |
| Intubation attempts allowed | ≤ 3 |
| Age (mean ± SD, yr) | 44.41 ± 9.45 |
| BMI (mean ± SD) | NR |
| Duration of anaesthetic (mean ± SD) | 77.33 ± 9.28 |
| Height (cm mean ± SD) | NR |
| Weight (kg mean ± SD) | 55.80 ± 9.07 |

Teymourian 2015 – Magnesium and ketamine gargle and postoperative sore throat

| **Characteristic** | **Value** |
| --- | --- |
| Country | Iran |
| Total sample size (N) | 100 |
| Female (N) | 47 |
| Smoking status (N) | NR |
| Length of follow-up (h) | 24 |
| Airway control | ETT |
| Cuff pressure (cm H₂O) | 20–30 |
| Intubation attempts allowed | ≤ 1 |
| Age (mean ± SD, yr) | 26.95 ± 10.83 |
| BMI (mean ± SD) | 21.90 ± 4.01 |
| Duration of anaesthetic (mean ± SD) | 54.15 ± 22.28 |
| Height (cm mean ± SD) | NR |
| Weight (kg mean ± SD) | 72.80 ± 17.87 |

Teymourian 2020 – Magnesium gargle versus ketamine gargle in postoperative sore throat pain: a randomized placebo-controlled clinical trial

| **Characteristic** | **Value** |
| --- | --- |
| Country | Iran (Islamic Republic of) |
| Total sample size (N) | 60 |
| Female (N) | 38 |
| Smoking status (N) | NR |
| Length of follow-up (h) | 24 |
| Airway control | ETT |
| Cuff pressure (cm H₂O) | 20–25 |
| Intubation attempts allowed | NR |
| Age (mean ± SD, yr) | 51.47 ± 8.49 |
| BMI (mean ± SD) | NR |
| Duration of anaesthetic (mean ± SD) | NR |
| Height (cm mean ± SD) | NR |
| Weight (kg mean ± SD) | NR |

Thapa 2017 – Betamethasone gel compared with lidocaine jelly to reduce tracheal tube related postoperative airway symptoms: a randomized controlled trial

| **Characteristic** | **Value** |
| --- | --- |
| Country | Nepal |
| Total sample size (N) | 120 |
| Female (N) | 78 |
| Smoking status (N) | NR |
| Length of follow-up (h) | 24 |
| Airway control | ETT |
| Cuff pressure (cm H₂O) | 25–34 |
| Intubation attempts allowed | ≤ 2 |
| Age (mean ± SD, yr) | 40.25 ± 12.74 |
| BMI (mean ± SD) | NR |
| Duration of anaesthetic (mean ± SD) | 100.88 ± 39.85 |
| Height (cm mean ± SD) | NR |
| Weight (kg mean ± SD) | 54.62 ± 10.15 |

Vaghela 2019 – Comparative evaluation of incidence of post-operative sore throat after nebulization with ketamine and lignocaine in patients undergoing general anaesthesia

| **Characteristic** | **Value** |
| --- | --- |
| Country | India |
| Total sample size (N) | 50 |
| Female (N) | 28 |
| Smoking status (N) | NR |
| Length of follow-up (h) | 24 |
| Airway control | ETT |
| Cuff pressure (cm H₂O) | 15–20 |
| Intubation attempts allowed | ≤ 2 |
| Age (mean ± SD, yr) | 29.60 ± 8.28 |
| BMI (mean ± SD) | NR |
| Duration of anaesthetic (mean ± SD) | 85.20 ± 4.03 |
| Height (cm mean ± SD) | NR |
| Weight (kg mean ± SD) | 51.46 ± 7.24 |

Yadav 2016 – Effect of magnesium sulfate nebulization on the incidence of postoperative sore throat

| **Characteristic** | **Value** |
| --- | --- |
| Country | India |
| Total sample size (N) | 100 |
| Female (N) | 47 |
| Smoking status (N) | NR |
| Length of follow-up (h) | 24 |
| Airway control | ETT |
| Cuff pressure (cm H₂O) | ≤ 20 |
| Intubation attempts allowed | NR |
| Age (mean ± SD, yr) | 40.85 ± 10.41 |
| BMI (mean ± SD) | NR |
| Duration of anaesthetic (mean ± SD) | NR |
| Height (cm mean ± SD) | NR |
| Weight (kg mean ± SD) | 59.64 ± 2.36 |

Yadav 2018 – Comparison of the effect of ketamine, tramadol, 1.5% saline and normal saline gargle on post-operative sore throat after endotracheal intubation

| **Characteristic** | **Value** |
| --- | --- |
| Country | India |
| Total sample size (N) | 100 |
| Female (N) | 46 |
| Smoking status (N) | NR |
| Length of follow-up (h) | 24 |
| Airway control | ETT |
| Cuff pressure (cm H₂O) | ≤ 20 |
| Intubation attempts allowed | ≤ 2 |
| Age (mean ± SD, yr) | 51.77 ± 11.67 |
| BMI (mean ± SD) | NR |
| Duration of anaesthetic (mean ± SD) | NR |
| Height (cm mean ± SD) | NR |
| Weight (kg mean ± SD) | 63.00 ± 9.55 |

## Tracheal Tube – Non-pharmacological

Abdi 2010 – Sparing the larynx during gynecological laparoscopy: a randomized trial comparing the LMA Supreme and the ETT

| **Characteristic** | **Value** |
| --- | --- |
| Country | France |
| Total sample size (N) | 138 |
| Female (N) | 138 |
| Smoking status (N) | 9 |
| Length of follow-up (h) | 168 |
| Airway control | ETT, LMA Supreme |
| Cuff pressure (cm H₂O) | ≤ 25 (ETT) / ≤ 50 (LMA) |
| Intubation attempts allowed | NR |
| Age (mean ± SD, yr) | 33.00 ± 8.48 |
| BMI (mean ± SD) | NR |
| Duration of anaesthetic (min, mean ± SD) | 55.50 ± 21.99 |
| Height (cm mean ± SD) | 164.50 ± 704.52 |
| Weight (kg mean ± SD) | 66.00 ± 15.52 |

Al-Metwalli 2011 – Is sealing cuff pressure, easy, reliable and safe technique for endotracheal tube cuff inflation: a comparative study

| **Characteristic** | **Value** |
| --- | --- |
| Country | Saudi Arabia |
| Total sample size (N) | 75 |
| Female (N) | 38 |
| Smoking status (N) | NR |
| Length of follow-up (h) | 24 |
| Airway control | ETT |
| Cuff pressure (cm H₂O) | As per trial arm |
| Intubation attempts allowed | ≤ 1 |
| Age (mean ± SD, yr) | 36.27 ± 10.45 |
| BMI (mean ± SD) | NR |
| Duration of anaesthetic (mean ± SD) | 113.37 ± 16.92 |
| Height (cm mean ± SD) | 166.23 ± 8.86 |
| Weight (kg mean ± SD) | 79.90 ± 12.79 |

Aqil 2017 – Incidence and severity of postoperative sore throat: a randomized comparison of Glidescope with Macintosh laryngoscope

| **Characteristic** | **Value** |
| --- | --- |
| Country | Saudi Arabia |
| Total sample size (N) | 140 |
| Female (N) | 81 |
| Smoking status (N) | 35 |
| Length of follow-up (h) | 24 |
| Airway control | ETT |
| Cuff pressure (cm H₂O) | 20–25 |
| Intubation attempts allowed | ≤ 2 |
| Age (mean ± SD, yr) | 37.70 ± 10.80 |
| BMI (mean ± SD) | 27.35 ± 3.92 |
| Duration of anaesthetic (mean ± SD) | 84.55 ± 27.99 |
| Height (cm mean ± SD) | 164.65 ± 7.74 |
| Weight (kg mean ± SD) | 73.95 ± 11.12 |

Bi 2022 – Effects of thermal softening of double-lumen endobronchial tubes on the prevention of postoperative sore throat in smokers: a randomized controlled trial

| **Characteristic** | **Value** |
| --- | --- |
| Country | China |
| Total sample size (N) | 258 |
| Female (N) | 61 |
| Smoking status (N) | 258 |
| Length of follow-up (h) | 72 |
| Airway control | DLT |
| Cuff pressure (cm H₂O) | ≤ 25 tracheal / ≤ 44 bronchial |
| Intubation attempts allowed | NR |
| Age (mean ± SD, yr) | 56.50 ± 8.63 |
| BMI (mean ± SD) | 23.70 ± 3.63 |
| Duration of anaesthetic (mean ± SD) | 180.50 ± 49.00 |
| Height (cm mean ± SD) | 165.00 ± 6.51 |
| Weight (kg mean ± SD) | 64.00 ± 12.18 |

Bolzan 2014 – Clinical use of the volume-time curve for endotracheal tube cuff management

| **Characteristic** | **Value** |
| --- | --- |
| Country | Brazil |
| Total sample size (N) | 450 |
| Female (N) | 108 |
| Smoking status (N) | NR |
| Length of follow-up (h) | 120 |
| Airway control | ETT |
| Cuff pressure (cm H₂O) | As per trial arm |
| Intubation attempts allowed | NR |
| Age (mean ± SD, yr) | 62.29 ± 7.80 |
| BMI (mean ± SD) | 26.15 ± 3.01 |
| Duration of anaesthetic (mean ± SD) | NR |
| Height (cm mean ± SD) | NR |
| Weight (kg mean ± SD) | NR |

Braz 2004 – Does sealing endotracheal tube cuff pressure diminish the frequency of postoperative laryngotracheal complaints after nitrous oxide anesthesia?

| **Characteristic** | **Value** |
| --- | --- |
| Country | Brazil |
| Total sample size (N) | 50 |
| Female (N) | 49 |
| Smoking status (N) | 8 |
| Length of follow-up (h) | 24 |
| Airway control | ETT |
| Cuff pressure (cm H₂O) | As per intervention arm |
| Intubation attempts allowed | ≤ 1 |
| Age (mean ± SD, yr) | 47.00 ± 6.68 |
| BMI (mean ± SD) | 26.00 ± 2.97 |
| Duration of anaesthetic (mean ± SD) | 264.00 ± 72.05 |
| Height (cm mean ± SD) | 158.00 ± 6.02 |
| Weight (kg mean ± SD) | 65.00 ± 9.47 |

Chang 2017 – Effect of endotracheal tube cuff shape on postoperative sore throat after endotracheal intubation

| **Characteristic** | **Value** |
| --- | --- |
| Country | Korea (Republic of) |
| Total sample size (N) | 200 |
| Female (N) | 97 |
| Smoking status (N) | NR |
| Length of follow-up (h) | 24 |
| Airway control | ETT |
| Cuff pressure (cm H₂O) | ≤ 25 |
| Intubation attempts allowed | ≤ 2 |
| Age (mean ± SD, yr) | 51.99 ± 16.03 |
| BMI (mean ± SD) | NR |
| Duration of anaesthetic (mean ± SD) | 169.00 ± 80.79 |
| Height (cm mean ± SD) | 162.50 ± 8.99 |
| Weight (kg mean ± SD) | 64.00 ± 10.48 |

Christiansen 2021 – How does tube size affect patients’ experiences of postoperative sore throat and hoarseness? A randomised controlled blinded study

| **Characteristic** | **Value** |
| --- | --- |
| Country | Denmark |
| Total sample size (N) | 261 |
| Female (N) | 236 |
| Smoking status (N) | 172 |
| Length of follow-up (h) | 96 |
| Airway control | ETT |
| Cuff pressure (cm H₂O) | 20–30 |
| Intubation attempts allowed | ≤ 2 |
| Age (mean ± SD, yr) | 50.90 ± 14.00 |
| BMI (mean ± SD) | 50.90 ± 4.30 |
| Duration of anaesthetic (mean ± SD) | 86.00 ± 43.50 |
| Height (cm mean ± SD) | NR |
| Weight (kg mean ± SD) | NR |

Cui 2024 – The feasibility and accuracy of the method for selecting the optimal size of double-lumen tube in thoracic surgery: a prospective, randomized controlled trial

| **Characteristic** | **Value** |
| --- | --- |
| Country | China |
| Total sample size (N) | 60 |
| Female (N) | 31 |
| Smoking status (N) | NR |
| Length of follow-up (h) | 24 |
| Airway control | DLT |
| Cuff pressure (cm H₂O) | 20–25 |
| Intubation attempts allowed | NR |
| Age (mean ± SD, yr) | 55.31 ± 12.66 |
| BMI (mean ± SD) | 24.15 ± 3.47 |
| Duration of anaesthetic (mean ± SD) | 128.40 ± 50.81 |
| Height (cm mean ± SD) | 166.71 ± 8.59 |
| Weight (kg mean ± SD) | 67.31 ± 11.97 |

Dutta 2020 – The impact of tracheal-tube introducer guided intubation in anticipated non-difficult airway on postoperative sore throat: a randomized controlled trial

| **Characteristic** | **Value** |
| --- | --- |
| Country | India |
| Total sample size (N) | 450 |
| Female (N) | 232 |
| Smoking status (N) | NR |
| Length of follow-up (h) | 24 |
| Airway control | ETT |
| Cuff pressure (cm H₂O) | ≤ 34 |
| Intubation attempts allowed | NR |
| Age (mean ± SD, yr) | 39.00 ± 12.67 |
| BMI (mean ± SD) | 26.67 ± 4.02 |
| Duration of anaesthetic (mean ± SD) | 86.63 ± 43.18 |
| Height (cm mean ± SD) | 162.33 ± 8.67 |
| Weight (kg mean ± SD) | 70.33 ± 13.02 |

Ganason 2019 – Post-operative sore throat: comparing the monitored endotracheal tube cuff pressure and pilot balloon palpation methods

| **Characteristic** | **Value** |
| --- | --- |
| Country | Malaysia |
| Total sample size (N) | 292 |
| Female (N) | 184 |
| Smoking status (N) | NR |
| Length of follow-up (h) | 48 |
| Airway control | ETT |
| Cuff pressure (cm H₂O) | As per trial arm |
| Intubation attempts allowed | ≤ 1 |
| Age (mean ± SD, yr) | 44.47 ± 19.21 |
| BMI (mean ± SD) | 25.15 ± 4.76 |
| Duration of anaesthetic (mean ± SD) | 151.62 ± 63.77 |
| Height (cm mean ± SD) | 160.60 ± 7.96 |
| Weight (kg mean ± SD) | 65.20 ± 12.91 |

Huh 2021 – Influence of two-handed jaw thrust during tracheal intubation on postoperative sore throat: a prospective randomised study

| **Characteristic** | **Value** |
| --- | --- |
| Country | Korea (Republic of) |
| Total sample size (N) | 92 |
| Female (N) | 48 |
| Smoking status (N) | 33 |
| Length of follow-up (h) | 24 |
| Airway control | ETT |
| Cuff pressure (cm H₂O) | 27 |
| Intubation attempts allowed | ≤ 1 |
| Age (mean ± SD, yr) | 62.00 ± 11.03 |
| BMI (mean ± SD) | 24.35 ± 3.53 |
| Duration of anaesthetic (min, mean ± SD) | 168.50 ± 53.49 |
| Height (cm mean ± SD) | 160.50 ± 9.13 |
| Weight (kg mean ± SD) | 62.50 ± 10.95 |

Jeon 2015 – Comparison of postoperative sore throat and hoarseness between two types of double-lumen endobronchial tubes: a randomized controlled trial

| **Characteristic** | **Value** |
| --- | --- |
| Country | Korea (Republic of); Japan; Macao; Germany; France; USA |
| Total sample size (N) | 60 |
| Female (N) | 30 |
| Smoking status (N) | 27 |
| Length of follow-up (h) | 24 |
| Airway control | DLT |
| Cuff pressure (cm H₂O) | 20 |
| Intubation attempts allowed | ≤ 2 |
| Age (mean ± SD, yr) | 61.00 ± 7.15 |
| BMI (mean ± SD) | NR |
| Duration of anaesthetic (min, mean ± SD) | 177.95 ± 64.63 |
| Height (cm mean ± SD) | 159.55 ± 9.05 |
| Weight (kg mean ± SD) | 61.00 ± 10.43 |

Kim 2022 – Effects of bevel direction of endotracheal tube on the postoperative sore throat when performing fiberoptic-guided tracheal intubation: a randomized controlled trial

| **Characteristic** | **Value** |
| --- | --- |
| Country | Korea (Republic of) |
| Total sample size (N) | 86 |
| Female (N) | 79 |
| Smoking status (N) | NR |
| Length of follow-up (h) | 24 |
| Airway control | ETT |
| Cuff pressure (cm H₂O) | 20–25 |
| Intubation attempts allowed | ≤ 1 |
| Age (mean ± SD, yr) | 49.49 ± 10.01 |
| BMI (mean ± SD) | NR |
| Duration of anaesthetic (min, mean ± SD) | NR |
| Height (cm mean ± SD) | 162.00 ± 5.17 |
| Weight (kg mean ± SD) | 60.00 ± 4.83 |

Kim 2023 – Slow advancement of the endotracheal tube during fiberoptic-guided tracheal intubation reduces the severity of postoperative sore throat

| **Characteristic** | **Value** |
| --- | --- |
| Country | Korea (Republic of); Ireland; Japan; USA |
| Total sample size (N) | 66 |
| Female (N) | 58 |
| Smoking status (N) | NR |
| Length of follow-up (h) | 24 |
| Airway control | ETT |
| Cuff pressure (cm H₂O) | 20–25 |
| Intubation attempts allowed | NR |
| Age (mean ± SD, yr) | 49.00 ± 10.58 |
| BMI (mean ± SD) | NR |
| Duration of anaesthetic (min, mean ± SD) | 130.00 ± 22.66 |
| Height (cm mean ± SD) | 160.50 ± 3.66 |
| Weight (kg mean ± SD) | 58.00 ± 6.94 |

Kundra 2024 – Comparative evaluation of incidence, severity, and relation of sore throat after endotracheal intubation at different cuff pressures

| **Characteristic** | **Value** |
| --- | --- |
| Country | India |
| Total sample size (N) | 100 |
| Female (N) | 100 |
| Smoking status (N) | NR |
| Length of follow-up (h) | 72 |
| Airway control | ETT |
| Cuff pressure (cm H₂O) | As per trial arm |
| Intubation attempts allowed | ≤ 1 |
| Age (mean ± SD, yr) | NR |
| BMI (mean ± SD) | NR |
| Duration of anaesthetic (min, mean ± SD) | 76.05 ± 32.39 |
| Height (cm mean ± SD) | NR |
| Weight (kg mean ± SD) | NR |

Liu 2010 – Correlations between controlled endotracheal tube cuff pressure and postprocedural complications: a multicenter study

| **Characteristic** | **Value** |
| --- | --- |
| Country | China |
| Total sample size (N) | 509 |
| Female (N) | 316 |
| Smoking status (N) | NR |
| Length of follow-up (h) | 24 |
| Airway control | ETT |
| Cuff pressure (cm H₂O) | As per trial arm |
| Intubation attempts allowed | ≤ 1 |
| Age (mean ± SD, yr) | 52.46 ± 16.99 |
| BMI (mean ± SD) | NR |
| Duration of anaesthetic (mean ± SD) | 165.22 ± 79.60 |
| Height (cm mean ± SD) | 167.56 ± 7.64 |
| Weight (kg mean ± SD) | 64.69 ± 9.98 |

Ozhan-Akdemir 2023 – The effect of the endotracheal tube cuff shape on post-operative sore throat in surgeries longer than 120 min in supine position

| **Characteristic** | **Value** |
| --- | --- |
| Country | Turkey |
| Total sample size (N) | 104 |
| Female (N) | 65 |
| Smoking status (N) | NR |
| Length of follow-up (h) | 24 |
| Airway control | ETT |
| Cuff pressure (cm H₂O) | ≤ 25 |
| Intubation attempts allowed | NR |
| Age (mean ± SD, yr) | 46.45 ± 14.70 |
| BMI (mean ± SD) | 26.55 ± 3.21 |
| Duration of anaesthetic (mean ± SD) | 153.45 ± 33.80 |
| Height (cm mean ± SD) | NR |
| Weight (kg mean ± SD) | NR |

Park 2020 – Comparison of conventional and fibreoptic-guided advance of left-sided double-lumen tube during endobronchial intubation: a randomised controlled trial

| **Characteristic** | **Value** |
| --- | --- |
| Country | Korea (Republic of) |
| Total sample size (N) | 136 |
| Female (N) | 16 |
| Smoking status (N) | 77 |
| Length of follow-up (h) | 24 |
| Airway control | DLT |
| Cuff pressure (cm H₂O) | ≤ 25 tracheal / ≤ 44 bronchial |
| Intubation attempts allowed | NR |
| Age (mean ± SD, yr) | 55.13 ± 15.34 |
| BMI (mean ± SD) | 24.15 ± 3.39 |
| Duration of anaesthetic (mean ± SD) | 132.40 ± 60.11 |
| Height (cm mean ± SD) | 168.95 ± 6.58 |
| Weight (kg mean ± SD) | 69.15 ± 11.55 |

Park 2020 – Two-handed jaw thrust decreases postoperative sore throat in patients undergoing double-lumen endobronchial intubation: a randomised study

| **Characteristic** | **Value** |
| --- | --- |
| Country | Korea (Republic of) |
| Total sample size (N) | 106 |
| Female (N) | 48 |
| Smoking status (N) | 47 |
| Length of follow-up (h) | 24 |
| Airway control | DLT |
| Cuff pressure (cm H₂O) | ≤ 25 tracheal / ≤ 44 bronchial |
| Intubation attempts allowed | ≤ 3 |
| Age (mean ± SD, yr) | 62.50 ± 13.45 |
| BMI (mean ± SD) | 24.15 ± 3.53 |
| Duration of anaesthetic (mean ± SD) | 214.50 ± 100.53 |
| Height (cm mean ± SD) | 162.00 ± 9.62 |
| Weight (kg mean ± SD) | 63.50 ± 10.96 |

Radu 2008 – Pharyngo-laryngeal discomfort after breast surgery: comparison between orotracheal intubation and laryngeal mask

| **Characteristic** | **Value** |
| --- | --- |
| Country | France |
| Total sample size (N) | 53 |
| Female (N) | 53 |
| Smoking status (N) | 8 |
| Length of follow-up (h) | 24 |
| Airway control | ETT, LMA |
| Cuff pressure (cm H₂O) | ≤ 27 (ETT) / ≤ 25 (LMA) |
| Intubation attempts allowed | ≤ 4 |
| Age (mean ± SD, yr) | 52.75 ± 12.80 |
| BMI (mean ± SD) | NR |
| Duration of anaesthetic (mean ± SD) | 104.03 ± 35.19 |
| Height (cm mean ± SD) | 163.62 ± 5.38 |
| Weight (kg mean ± SD) | 65.21 ± 12.51 |

Seo 2016 – The effects of thermal softening of double-lumen endobronchial tubes on postoperative sore throat, hoarseness and vocal cord injuries: a prospective double-blind randomized trial

| **Characteristic** | **Value** |
| --- | --- |
| Country | Korea (Republic of) |
| Total sample size (N) | 140 |
| Female (N) | 63 |
| Smoking status (N) | 91 |
| Length of follow-up (h) | 72 |
| Airway control | DLT |
| Cuff pressure (cm H₂O) | 25 tracheal / 44 bronchial |
| Intubation attempts allowed | ≤ 1 |
| Age (mean ± SD, yr) | 57.50 ± 11.10 |
| BMI (mean ± SD) | 23.00 ± 3.00 |
| Duration of anaesthetic (mean ± SD) | 196.50 ± 66.31 |
| Height (cm mean ± SD) | 88.00 ± 75.80 |
| Weight (kg mean ± SD) | 61.00 ± 9.53 |

Sprague 1987 – Magill versus Mallinckrodt tracheal tubes: a comparative study of postoperative sore throat

| **Characteristic** | **Value** |
| --- | --- |
| Country | United Kingdom |
| Total sample size (N) | 100 |
| Female (N) | 100 |
| Smoking status (N) | 40 |
| Length of follow-up (h) | 24 |
| Airway control | ETT |
| Cuff pressure (cm H₂O) | As per trial arm |
| Intubation attempts allowed | NR |
| Age (mean ± SD, yr) | 38.10 ± 15.13 |
| BMI (mean ± SD) | NR |
| Duration of anaesthetic (mean ± SD) | NR |
| Height (cm mean ± SD) | NR |
| Weight (kg mean ± SD) | NR |

Tosh 2019 – Incidence and severity of postoperative pharyngolaryngeal complications following use of Baska Mask versus endotracheal intubation

| **Characteristic** | **Value** |
| --- | --- |
| Country | India |
| Total sample size (N) | 120 |
| Female (N) | 72 |
| Smoking status (N) | NR |
| Length of follow-up (h) | 24 |
| Airway control | ETT, Baska Mask |
| Cuff pressure (cm H₂O) | 20–22 |
| Intubation attempts allowed | ≤ 3 |
| Age (mean ± SD, yr) | 48.29 ± 10.59 |
| BMI (mean ± SD) | NR |
| Duration of anaesthetic (mean ± SD) | 93.74 ± 8.71 |
| Height (cm mean ± SD) | NR |
| Weight (kg mean ± SD) | 63.35 ± 9.68 |

Wang 2024 – Effect of continuous measurement and adjustment of endotracheal tube cuff pressure on postoperative sore throat in patients undergoing gynecological laparoscopic surgery: a randomized controlled trial

| **Characteristic** | **Value** |
| --- | --- |
| Country | China |
| Total sample size (N) | 60 |
| Female (N) | 60 |
| Smoking status (N) | 0 |
| Length of follow-up (h) | 48 |
| Airway control | ETT |
| Cuff pressure (cm H₂O) | As per trial arm |
| Intubation attempts allowed | ≤ 1 |
| Age (mean ± SD, yr) | 41.85 ± 10.49 |
| BMI (mean ± SD) | 23.10 ± 3.03 |
| Duration of anaesthetic (mean ± SD) | 187.50 ± 68.58 |
| Height (cm mean ± SD) | NR |
| Weight (kg mean ± SD) | NR |

Yan 2023 – Effect of thermal softening of double-lumen endobronchial tubes on postoperative sore throat in patients with prior SARS-CoV-2 infection: a randomized controlled trial

| **Characteristic** | **Value** |
| --- | --- |
| Country | China |
| Total sample size (N) | 120 |
| Female (N) | 56 |
| Smoking status (N) | 54 |
| Length of follow-up (h) | 24 |
| Airway control | DLT |
| Cuff pressure (cm H₂O) | ≤ 25 tracheal / ≤ 44 bronchial |
| Intubation attempts allowed | NR |
| Age (mean ± SD, yr) | 59.50 ± 10.48 |
| BMI (mean ± SD) | 23.50 ± 3.68 |
| Duration of anaesthetic (mean ± SD) | 155.00 ± 31.47 |
| Height (cm mean ± SD) | 162.00 ± 7.55 |
| Weight (kg mean ± SD) | 61.50 ± 9.14 |

Yoneda 1999 – A simple method to control tracheal cuff pressure in anaesthesia and in air evacuation

| **Characteristic** | **Value** |
| --- | --- |
| Country | Japan |
| Total sample size (N) | 125 |
| Female (N) | 72 |
| Smoking status (N) | NR |
| Length of follow-up (h) | 96 |
| Airway control | ETT |
| Cuff pressure (cm H₂O) | As per arm |
| Intubation attempts allowed | NR |
| Age (mean ± SD, yr) | 45.92 ± 17.39 |
| BMI (mean ± SD) | NR |
| Duration of anaesthetic (min, mean ± SD) | 170.49 ± 50.78 |
| Height (cm mean ± SD) | 162.83 ± 10.75 |
| Weight (kg mean ± SD) | 61.38 ± 11.69 |

Yoon 2019 – Postoperative sore throat and subglottic injury after McGrath R MAC videolaryngoscopic intubation with versus without a stylet in patients with a high Mallampati score: a randomized controlled trial

| **Characteristic** | **Value** |
| --- | --- |
| Country | Korea (Republic of) |
| Total sample size (N) | 104 |
| Female (N) | 54 |
| Smoking status (N) | NR |
| Length of follow-up (h) | 24 |
| Airway control | ETT |
| Cuff pressure (cm H₂O) | ≤ 25 |
| Intubation attempts allowed | ≤ 3 |
| Age (mean ± SD, yr) | 59.45 ± 14.89 |
| BMI (mean ± SD) | 25.40 ± 3.80 |
| Duration of anaesthetic (min, mean ± SD) | 216.95 ± 132.78 |
| Height (cm mean ± SD) | NR |
| Weight (kg mean ± SD) | NR |

Yu 2021 – Effects of thermal softening of endotracheal tubes on postoperative sore throat: a randomized double-blinded trial

| **Characteristic** | **Value** |
| --- | --- |
| Country | Korea (Republic of) |
| Total sample size (N) | 196 |
| Female (N) | 63 |
| Smoking status (N) | 45 |
| Length of follow-up (h) | 24 |
| Airway control | ETT |
| Cuff pressure (cm H₂O) | 20–25 |
| Intubation attempts allowed | NR |
| Age (mean ± SD, yr) | 49.48 ± 17.52 |
| BMI (mean ± SD) | 24.50 ± 3.21 |
| Duration of anaesthetic (min, mean ± SD) | 115.57 ± 54.22 |
| Height (cm mean ± SD) | 166.12 ± 9.49 |
| Weight (kg mean ± SD) | 68.13 ± 13.44 |

Yuzkat 2019 – Effect of using the suction above cuff endotracheal tube (SACETT) on postoperative respiratory complications in rhinoplasty: a randomized prospective controlled trial

| **Characteristic** | **Value** |
| --- | --- |
| Country | Turkey |
| Total sample size (N) | 132 |
| Female (N) | 77 |
| Smoking status (N) | 43 |
| Length of follow-up (h) | 1 |
| Airway control | ETT (per arm) |
| Cuff pressure (cm H₂O) | 20–30 |
| Intubation attempts allowed | NR |
| Age (mean ± SD, yr) | 25.95 ± 6.80 |
| BMI (mean ± SD) | 23.25 ± 2.93 |
| Duration of anaesthetic (min, mean ± SD) | 98.80 ± 10.14 |
| Height (cm mean ± SD) | NR |
| Weight (kg mean ± SD) | 67.05 ± 11.94 |

Zhu 2024 – The effect of different endotracheal tube cuff pressure monitoring systems on postoperative sore throat in patients undergoing tracheal intubation: a randomized clinical trial

| **Characteristic** | **Value** |
| --- | --- |
| Country | China |
| Total sample size (N) | 114 |
| Female (N) | 57 |
| Smoking status (N) | 0 |
| Length of follow-up (h) | 48 |
| Airway control | ETT |
| Cuff pressure (cm H₂O) | As per trial arm |
| Intubation attempts allowed | ≤ 1 |
| Age (mean ± SD, yr) | 51.70 ± 11.23 |
| BMI (mean ± SD) | 24.70 ± 2.80 |
| Duration of anaesthetic (min, mean ± SD) | 169.81 ± 54.44 |
| Height (cm mean ± SD) | NR |
| Weight (kg mean ± SD) | NR |

## Supraglottic airway devices

Altinsoy 2020 – The effects of topical chlorhexidine-benzydamine spray on laryngeal mask airway application

| **Characteristic** | **Value** |
| --- | --- |
| Country | Turkey |
| Total sample size (N) | 100 |
| Female (N) | 27 |
| Smoking status (N) | NR |
| Length of follow-up (h) | 24 |
| Airway control | LMA (weight-based sizing) |
| Cuff pressure (cm H₂O) | NR |
| Intubation attempts allowed | ≤ 3 |
| Age (mean ± SD, yr) | 45.35 ± 16.34 |
| BMI (mean ± SD) | 26.70 ± 4.30 |
| Duration of anaesthetic (min, mean ± SD) | 44.05 ± 16.12 |
| Height (cm mean ± SD) | 170.65 ± 8.30 |
| Weight (kg mean ± SD) | 77.65 ± 12.60 |

Amini 2007 – A comparison of the disposable vs the reusable laryngeal tube in paralysed adult patients

| **Characteristic** | **Value** |
| --- | --- |
| Country | Iran (Islamic Republic of) |
| Total sample size (N) | 100 |
| Female (N) | 17 |
| Smoking status (N) | NR |
| Length of follow-up (h) | 4 |
| Airway control | Laryngeal tube (disposable vs reusable) |
| Cuff pressure (cm H₂O) | NR |
| Intubation attempts allowed | ≤ 3 |
| Age (mean ± SD, yr) | 32.61 ± 12.38 |
| BMI (mean ± SD) | NR |
| Duration of anaesthetic (min, mean ± SD) | NR |
| Height (cm mean ± SD) | 174.07 ± 7.51 |
| Weight (kg mean ± SD) | 72.24 ± 11.64 |

Burgard 1996 – The effect of laryngeal mask cuff pressure on postoperative sore throat incidence

| **Characteristic** | **Value** |
| --- | --- |
| Country | Germany |
| Total sample size (N) | 200 |
| Female (N) | 200 |
| Smoking status (N) | NR |
| Length of follow-up (h) | 24 |
| Airway control | LMA |
| Cuff pressure (cm H₂O) | As per trial arm |
| Intubation attempts allowed | ≤ 3 |
| Age (mean ± SD, yr) | 42.00 ± 13.16 |
| BMI (mean ± SD) | NR |
| Duration of anaesthetic (min, mean ± SD) | 104.50 ± 53.96 |
| Height (cm mean ± SD) | 166.50 ± 6.67 |
| Weight (kg mean ± SD) | 65.00 ± 6.58 |

Chairatthanawanit 2017 – Benzydamine hydrochloride spray for reducing postoperative sore throat after general anaesthesia with laryngeal mask airway

| **Characteristic** | **Value** |
| --- | --- |
| Country | Thailand |
| Total sample size (N) | 80 |
| Female (N) | 43 |
| Smoking status (N) | NR |
| Length of follow-up (h) | 4 |
| Airway control | LMA |
| Cuff pressure (cm H₂O) | NR |
| Intubation attempts allowed | ≤ 3 |
| Age (mean ± SD, yr) | 47.75 ± 15.42 |
| BMI (mean ± SD) | 23.65 ± 3.45 |
| Duration of anaesthetic (min, mean ± SD) | 102.75 ± 51.65 |
| Height (cm mean ± SD) | NR |
| Weight (kg mean ± SD) | NR |

Chandra 2018 – Comparison between lidocaine inhalation and intravenous dexamethasone in reducing postoperative sore throat frequency after laryngeal mask insertion

| **Characteristic** | **Value** |
| --- | --- |
| Country | Indonesia |
| Total sample size (N) | 128 |
| Female (N) | 55 |
| Smoking status (N) | 0 |
| Length of follow-up (h) | 2 |
| Airway control | LMA |
| Cuff pressure (cm H₂O) | NR |
| SAD cuff pressure (cm H₂O) | 40 |
| Intubation attempts allowed | ≤ 1 |
| Age (mean ± SD, yr) | 42.00 ± 10.02 |
| BMI (mean ± SD) | 22.29 ± 1.70 |
| Duration of anaesthetic (min ± SD) | 68.13 ± 15.47 |
| Height (cm ± SD) | 162.50 ± 5.41 |
| Weight (kg ± SD) | 61.13 ± 6.08 |

Deepak 2021 – Comparison of Ambu AuraGain at low cuff pressure, Ambu AuraGain at high cuff pressure and i-gel in relation to incidence of postoperative upper airway complications

| **Characteristic** | **Value** |
| --- | --- |
| Country | India |
| Total sample size (N) | 200 |
| Female (N) | 38 |
| Smoking status (N) | NR |
| Length of follow-up (h) | 72 |
| Airway control | I-gel |
| Cuff pressure (cm H₂O) | NR |
| SAD cuff pressure (cm H₂O) | as per trial arm |
| Intubation attempts allowed | ≤ 3 |
| Age (mean ± SD, yr) | 34.90 ± 10.66 |
| BMI (mean ± SD) | 22.62 ± 3.44 |
| Duration of anaesthetic (min ± SD) | 69.53 ± 36.03 |
| Height (cm ± SD) | NR |
| Weight (kg ± SD) | NR |

Figueredo 1999 – Laryngo-pharyngeal complaints after use of the laryngeal mask airway

| **Characteristic** | **Value** |
| --- | --- |
| Country | Spain |
| Total sample size (N) | 120 |
| Female (N) | 32 |
| Smoking status (N) | NR |
| Length of follow-up (h) | 24 |
| Airway control | LMA |
| Cuff pressure (cm H₂O) | NR |
| SAD cuff pressure (cm H₂O) | Measured |
| Intubation attempts allowed | NR |
| Age (mean ± SD, yr) | 46.33 ± 16.38 |
| BMI (mean ± SD) | NR |
| Duration of anaesthetic (min ± SD) | 59.05 ± 14.73 |
| Height (cm ± SD) | 170.94 ± 7.01 |
| Weight (kg ± SD) | 76.04 ± 8.52 |

Gunenc 2015 – Effect of laryngeal mask cuff pressure on postoperative pharyngo-laryngeal morbidity in geriatric patients

| **Characteristic** | **Value** |
| --- | --- |
| Country | Turkey |
| Total sample size (N) | 90 |
| Female (N) | 45 |
| Smoking status (N) | NR |
| Length of follow-up (h) | 24 |
| Airway control | SAD |
| Cuff pressure (cm H₂O) | NR |
| SAD cuff pressure (cm H₂O) | as per trial arm |
| Intubation attempts allowed | ≤ 2 |
| Age (mean ± SD, yr) | 72.35 ± 5.80 |
| BMI (mean ± SD) | NR |
| Duration of anaesthetic (min ± SD) | 56.30 ± 32.18 |
| Height (cm ± SD) | NR |
| Weight (kg ± SD) | 72.40 ± 12.16 |

Hu 2017 – Comparison of laryngeal mask airway insertion methods, including the external larynx lift with pre-inflated cuff, on postoperative pharyngolaryngeal complications

| **Characteristic** | **Value** |
| --- | --- |
| Country | USA |
| Total sample size (N) | 450 |
| Female (N) | 185 |
| Smoking status (N) | NR |
| Length of follow-up (h) | 24 |
| Airway control | LMA Unique |
| Cuff pressure (cm H₂O) | As measured individually |
| SAD cuff pressure (cm H₂O) | 120 |
| Intubation attempts allowed | NR |
| Age (mean ± SD, yr) | 46.00 ± 15.98 |
| BMI (mean ± SD) | 27.63 ± 5.49 |
| Duration of anaesthetic (min ± SD) | 73.66 ± 38.63 |
| Height (cm ± SD) | 174.00 ± 11.32 |
| Weight (kg ± SD) | 84.00 ± 19.34 |

Jeon 2011 – Effect of continuous cuff pressure regulator in general anaesthesia with laryngeal mask airway

| **Characteristic** | **Value** |
| --- | --- |
| Country | Korea (Republic of) |
| Total sample size (N) | 60 |
| Female (N) | 50 |
| Smoking status (N) | NR |
| Length of follow-up (h) | 24 |
| Airway control | LMA Classic |
| Cuff pressure (cm H₂O) | NR |
| SAD cuff pressure (cm H₂O) | as per trial arm |
| Intubation attempts allowed | ≤ 3 |
| Age (mean ± SD, yr) | 51.05 ± 14.73 |
| BMI (mean ± SD) | NR |
| Duration of anaesthetic (min ± SD) | 89.00 ± 27.05 |
| Height (cm ± SD) | 158.40 ± 8.08 |
| Weight (kg ± SD) | 60.25 ± 9.42 |

Joe 2012 – The effect of cuff pressure on postoperative sore throat after Cobra perilaryngeal airway

| **Characteristic** | **Value** |
| --- | --- |
| Country | Korea (Republic of) |
| Total sample size (N) | 174 |
| Female (N) | 75 |
| Smoking status (N) | NR |
| Length of follow-up (h) | 24 |
| Airway control | Cobra-PLA |
| Cuff pressure (cm H₂O) | NR |
| SAD cuff pressure (cm H₂O) | as per trial arm |
| Intubation attempts allowed | ≤ 2 |
| Age (mean ± SD, yr) | 36.00 ± 15.11 |
| BMI (mean ± SD) | 23.64 ± 5.01 |
| Duration of anaesthetic (min ± SD) | 55.52 ± 22.55 |
| Height (cm ± SD) | 167.00 ± 8.50 |
| Weight (kg ± SD) | 64.99 ± 11.53 |

Kang 2014 – Postoperative pharyngolaryngeal adverse events with laryngeal mask airway (LMA Supreme) in laparoscopic surgical procedures with cuff pressure limiting 25 cm H₂O: prospective, blind, and randomised study

| **Characteristic** | **Value** |
| --- | --- |
| Country | Korea (Republic of) |
| Total sample size (N) | 101 |
| Female (N) | 62 |
| Smoking status (N) | NR |
| Length of follow-up (h) | 48 |
| Airway control | LMA |
| Cuff pressure (cm H₂O) | NR |
| SAD cuff pressure (cm H₂O) | as per trial arm |
| Intubation attempts allowed | ≤ 3 |
| Age (mean ± SD, yr) | 41.50 ± 14.44 |
| BMI (mean ± SD) | NR |
| Duration of anaesthetic (min ± SD) | 86.00 ± 31.34 |
| Height (cm ± SD) | 163.50 ± 7.49 |
| Weight (kg ± SD) | 64.50 ± 11.99 |

Kati 2004 – Does benzydamine hydrochloride applied preemptively reduce sore throat due to laryngeal mask airway?

| **Characteristic** | **Value** |
| --- | --- |
| Country | Turkey |
| Total sample size (N) | 100 |
| Female (N) | 35 |
| Smoking status (N) | 0 |
| Length of follow-up (h) | 4 |
| Airway control | LMA Classic |
| Cuff pressure (cm H₂O) | NR |
| SAD cuff pressure (cm H₂O) | as per Table 1 |
| Intubation attempts allowed | ≤ 1 |
| Age (mean ± SD, yr) | 39.83 ± 14.10 |
| BMI (mean ± SD) | NR |
| Duration of anaesthetic (min ± SD) | NR |
| Height (cm ± SD) | NR |
| Weight (kg ± SD) | NR |

Kihara 2001 – Routine use of the intubating laryngeal mask air-way results in increased upper airway morbidity

| **Characteristic** | **Value** |
| --- | --- |
| Country | Japan |
| Total sample size (N) | 65 |
| Female (N) | 65 |
| Smoking status (N) | 49 |
| Length of follow-up (h) | 48 |
| Airway control | LMA Classic vs Intubating LMA |
| Cuff pressure (cm H₂O) | NR |
| SAD cuff pressure (cm H₂O) | 60 |
| Intubation attempts allowed | ≤ 3 |
| Age (mean ± SD, yr) | 42.03 ± 14.06 |
| BMI (mean ± SD) | NR |
| Duration of anaesthetic (min ± SD) | 103.98 ± 48.32 |
| Height (cm ± SD) | 157.49 ± 6.96 |
| Weight (kg ± SD) | 58.48 ± 11.51 |

Kim 2007 – A heated humidifier does not reduce laryngo-pharyngeal complaints after brief laryngeal mask anesthesia

| **Characteristic** | **Value** |
| --- | --- |
| Country | Korea (Republic of) |
| Total sample size (N) | 200 |
| Female (N) | 90 |
| Smoking status (N) | 46 |
| Length of follow-up (h) | 24 |
| Airway control | LMA |
| Cuff pressure (cm H₂O) | NR |
| SAD cuff pressure (cm H₂O) | as per arm |
| Intubation attempts allowed | ≤ 2 |
| Age (mean ± SD, yr) | 38.55 ± 13.90 |
| BMI (mean ± SD) | 23.20 ± 3.51 |
| Duration of anaesthetic (min ± SD) | 89.99 ± 32.28 |
| Height (cm ± SD) | 165.01 ± 7.86 |
| Weight (kg ± SD) | 63.35 ± 11.99 |

Kiran 2012 – Postoperative sore throat with 0.05% betamethasone gel and 2% lignocaine jelly used as a lubricant for ProSeal LMA (PLMA) insertion

| **Characteristic** | **Value** |
| --- | --- |
| Country | India |
| Total sample size (N) | 60 |
| Female (N) | 29 |
| Smoking status (N) | NR |
| Length of follow-up (h) | 24 |
| Airway control | PLMA |
| Cuff pressure (cm H₂O) | NR |
| SAD cuff pressure (cm H₂O) | 60 |
| Intubation attempts allowed | NR |
| Age (mean ± SD, yr) | 31.48 ± 12.41 |
| BMI (mean ± SD) | NR |
| Duration of anaesthetic (min ± SD) | 47.50 ± 6.61 |
| Height (cm ± SD) | NR |
| Weight (kg ± SD) | 61.51 |

Koay 2001 – A randomized trial comparing two laryngeal mask airway insertion techniques

| **Characteristic** | **Value** |
| --- | --- |
| Country | Singapore |
| Total sample size (N) | 149 |
| Female (N) | NR |
| Smoking status (N) | NR |
| Length of follow-up (h) | 24 |
| Airway control | LMA |
| Cuff pressure (cm H₂O) | NR |
| SAD cuff pressure (cm H₂O) | as per trial arm |
| Intubation attempts allowed | ≤ 2 |
| Age (mean ± SD, yr) | 35.98 ± 12.50 |
| BMI (mean ± SD) | NR |
| Duration of anaesthetic (min ± SD) | NR |
| Height (cm ± SD) | NR |
| Weight (kg ± SD) | 63.47 ± 11.62 |

Li 2015 – Application of minimum effective cuff inflating volume for laryngeal mask airway and its impact on postoperative pharyngeal complications

| **Characteristic** | **Value** |
| --- | --- |
| Country | China |
| Total sample size (N) | 105 |
| Female (N) | 27 |
| Smoking status (N) | NR |
| Length of follow-up (h) | 48 |
| Airway control | LMA Classic |
| Cuff pressure (cm H₂O) | NR |
| SAD cuff pressure (cm H₂O) | as per trial arm |
| Intubation attempts allowed | ≤ 2 |
| Age (mean ± SD, yr) | 60.85 ± 13.20 |
| BMI (mean ± SD) | 23.40 ± 2.10 |
| Duration of anaesthetic (min ± SD) | 74.30 ± 36.72 |
| Height (cm ± SD) | NR |
| Weight (kg ± SD) | NR |

Li 2021 – Incidence of postoperative sore throat after using a new technique of insertion of a second generation Laryngeal Mask Airway: a randomised controlled trial

| **Characteristic** | **Value** |
| --- | --- |
| Country | China |
| Total sample size (N) | 408 |
| Female (N) | 196 |
| Smoking status (N) | NR |
| Length of follow-up (h) | 1 |
| Airway control | SLMA |
| Cuff pressure (cm H₂O) | NR |
| SAD cuff pressure (cm H₂O) | 60 |
| Intubation attempts allowed | ≤ 2 |
| Age (mean ± SD, yr) | 40.85 ± 13.39 |
| BMI (mean ± SD) | 24.25 ± 3.45 |
| Duration of anaesthetic (min ± SD) | 110.80 ± 11.97 |
| Height (cm ± SD) | NR |
| Weight (kg ± SD) | NR |

Lv 2023 – Effect of superior laryngeal nerve block in alleviating sore throat after application of i-gel supraglottic airway: a randomized controlled trial

| **Characteristic** | **Value** |
| --- | --- |
| Country | China |
| Total sample size (N) | 140 |
| Female (N) | 134 |
| Smoking status (N) | NR |
| Length of follow-up (h) | 24 |
| Airway control | I-Gel |
| Cuff pressure (cm H₂O) | NR |
| SAD cuff pressure (cm H₂O) | NR |
| Intubation attempts allowed | ≤ 1 |
| Age (mean ± SD, yr) | 53.50 ± 8.45 |
| BMI (mean ± SD) | 23.61 ± 1.70 |
| Duration of anaesthetic (min ± SD) | 96.50 ± 42.45 |
| Height (cm ± SD) | NR |
| Weight (kg ± SD) | NR |

Naseem 2022 – Evaluation of the efficacy of turmeric-based lozenges for the prevention of postoperative sore throat in surgeries done under laryngeal mask airway insertion

| **Characteristic** | **Value** |
| --- | --- |
| Country | India |
| Total sample size (N) | 139 |
| Female (N) | 55 |
| Smoking status (N) | NR |
| Length of follow-up (h) | 24 |
| Airway control | LMA |
| Cuff pressure (cm H₂O) | NR |
| SAD cuff pressure (cm H₂O) | ≤ 34 |
| Intubation attempts allowed | ≤ 1 |
| Age (mean ± SD, yr) | 42.32 ± 11.33 |
| BMI (mean ± SD) | NR |
| Duration of anaesthetic (min ± SD) | 73.97 ± 16.61 |
| Height (cm ± SD) | NR |
| Weight (kg ± SD) | 61.93 ± 7.72 |

Peng 2024 – Comparison of lidocaine viscous gargle and topical application on laryngeal mask airway in general anesthesia

| **Characteristic** | **Value** |
| --- | --- |
| Country | China |
| Total sample size (N) | 90 |
| Female (N) | 35 |
| Smoking status (N) | NR |
| Length of follow-up (h) | 24 |
| Airway control | LMA |
| Cuff pressure (cm H₂O) | NR |
| SAD cuff pressure (cm H₂O) | 60 |
| Intubation attempts allowed | ≤ 2 |
| Age (mean ± SD, yr) | 52.05 ± 11.55 |
| BMI (mean ± SD) | 24.15 ± 2.51 |
| Duration of anaesthetic (min ± SD) | 57.65 ± 27.44 |
| Height (cm ± SD) | 166.05 ± 8.10 |
| Weight (kg ± SD) | 67.00 ± 11.21 |

Rashwan 2014 – Effect of tramadol gargle on postoperative sore throat: a double blinded randomized placebo controlled study

| **Characteristic** | **Value** |
| --- | --- |
| Country | Kuwait |
| Total sample size (N) | 50 |
| Female (N) | 18 |
| Smoking status (N) | 0 |
| Length of follow-up (h) | 24 |
| Airway control | LMA |
| Cuff pressure (cm H₂O) | NR |
| SAD cuff pressure (cm H₂O) | 50–60 |
| Intubation attempts allowed | NR |
| Age (mean ± SD, yr) | 43.50 ± 12.99 |
| BMI (mean ± SD) | NR |
| Duration of anaesthetic (min ± SD) | 51.50 ± 7.46 |
| Height (cm ± SD) | NR |
| Weight (kg ± SD) | 79.50 ± 6.63 |

Rieger 1997 – Intracuff pressures do not predict laryngopharyngeal discomfort after use of the laryngeal mask airway

| **Characteristic** | **Value** |
| --- | --- |
| Country | Germany |
| Total sample size (N) | 70 |
| Female (N) | 61 |
| Smoking status (N) | NR |
| Length of follow-up (h) | 48 |
| Airway control | LMA |
| Cuff pressure (cm H₂O) | NR |
| SAD cuff pressure (cm H₂O) | as per trial arm |
| Intubation attempts allowed | ≤ 1 |
| Age (mean ± SD, yr) | 52.02 ± 15.40 |
| BMI (mean ± SD) | 25.00 ± 2.97 |
| Duration of anaesthetic (min ± SD) | 100.52 ± 40.61 |
| Height (cm ± SD) | 165.98 ± 5.06 |
| Weight (kg ± SD) | 67.02 ± 8.07 |

Seet 2010 – Use of manometry for laryngeal mask airway reduces postoperative pharyngolaryngeal adverse events: a prospective, randomized trial

| **Characteristic** | **Value** |
| --- | --- |
| Country | Canada |
| Total sample size (N) | 203 |
| Female (N) | 90 |
| Smoking status (N) | NR |
| Length of follow-up (h) | 24 |
| Airway control | LMA |
| Cuff pressure (cm H₂O) | NR |
| SAD cuff pressure (cm H₂O) | as per trial arm |
| Intubation attempts allowed | ≤ 3 |
| Age (mean ± SD, yr) | 45.97 ± 15.99 |
| BMI (mean ± SD) | NR |
| Duration of anaesthetic (min ± SD) | 51.45 ± 27.98 |
| Height (cm ± SD) | 170.97 ± 11.09 |
| Weight (kg ± SD) | 81.94 ± 16.09 |

Sundaraj 2022 – Preoperative amylmetacresol and dichlorobenzyl alcohol with lignocaine lozenge reduces postoperative sore throat following general anaesthesia using supraglottic airway devices: a double-blinded, randomised, placebo-controlled trial

| **Characteristic** | **Value** |
| --- | --- |
| Country | Malaysia |
| Total sample size (N) | 104 |
| Female (N) | 65 |
| Smoking status (N) | NR |
| Length of follow-up (h) | 24 |
| Airway control | LMA |
| Cuff pressure (cm H₂O) | NR |
| SAD cuff pressure (cm H₂O) | ≤ 60 |
| Intubation attempts allowed | ≤ 1 (per protocol) |
| Age (mean ± SD, yr) | 45.79 ± 14.82 |
| BMI (mean ± SD) | 25.22 ± 4.49 |
| Duration of anaesthetic (min ± SD) | 66.43 ± 45.41 |
| Height (cm ± SD) | NR |
| Weight (kg ± SD) | NR |

Taghavi Gilani 2015 – Reducing sore throat following laryngeal mask airway insertion: comparing lidocaine gel, saline, and washing mouth with the control group

| **Characteristic** | **Value** |
| --- | --- |
| Country | Iran (Islamic Republic of) |
| Total sample size (N) | 240 |
| Female (N) | 108 |
| Smoking status (N) | NR |
| Length of follow-up (h) | 24 |
| Airway control | LMA |
| Cuff pressure (cm H₂O) | as per trial arm |
| SAD cuff pressure (cm H₂O) | ≤ 200 (as per trial arm) |
| Intubation attempts allowed | NR |
| Age (mean ± SD, yr) | 61.42 ± 8.99 |
| BMI (mean ± SD) | NR |
| Duration of anaesthetic (min ± SD) | 52.52 ± 19.64 |
| Height (cm ± SD) | NR |
| Weight (kg ± SD) | NR |

Uzture 2014 – The effect of flurbiprofen on postoperative sore throat and hoarseness after LMA-ProSeal insertion: a randomised, clinical trial

| **Characteristic** | **Value** |
| --- | --- |
| Country | Turkey |
| Total sample size (N) | 80 |
| Female (N) | 39 |
| Smoking status (N) | NR |
| Length of follow-up (h) | 24 |
| Airway control | LMA |
| Cuff pressure (cm H₂O) | NR |
| SAD cuff pressure (cm H₂O) | ≤ 60 |
| Intubation attempts allowed | ≤ 1 |
| Age (mean ± SD, yr) | 41.00 ± 13.95 |
| BMI (mean ± SD) | NR |
| Duration of anaesthetic (min ± SD) | 44.00 ± 17.27 |
| Height (cm ± SD) | NR |
| Weight (kg ± SD) | 74.00 ± 9.95 |

Vasanth Karthik 2014 – Does cuff pressure monitoring reduce postoperative pharyngolaryngeal adverse events after LMA-ProSeal insertion? A parallel group randomised trial

| **Characteristic** | **Value** |
| --- | --- |
| Country | India |
| Total sample size (N) | 120 |
| Female (N) | 105 |
| Smoking status (N) | NR |
| Length of follow-up (h) | 24 |
| Airway control | LMA-ProSeal |
| Cuff pressure (cm H₂O ETT tube) | NR |
| Cuff pressure (cm H₂O SAD) | as per trial arm |
| Intubation attempts allowed | ≤ 3 |
| Age (mean ± SD, yr) | 49.09 ± 13.01 |
| BMI (mean ± SD) | NR |
| Duration of anaesthetic (min ± SD) | 110.11 ± 47.89 |
| Height (cm mean ± SD) | NR |
| Weight (kg mean ± SD) | 59.41 ± 10.21 |

Wang 2020 – Effects of preoperative gum chewing on sore throat after general anesthesia with a supraglottic airway device: a randomized controlled trial

| **Characteristic** | **Value** |
| --- | --- |
| Country | China |
| Total sample size (N) | 140 |
| Female (N) | 138 |
| Smoking status (N) | 0 |
| Length of follow-up (h) | 24 |
| Airway control | SLIPA |
| Cuff pressure (cm H₂O ETT tube) | NR |
| Cuff pressure (cm H₂O SAD) | NR |
| Intubation attempts allowed | ≤ 2 |
| Age (mean ± SD, yr) | 43.20 ± 12.50 |
| BMI (mean ± SD) | 21.60 ± 1.31 |
| Duration of anaesthetic (min ± SD) | 13.15 ± 11.07 |
| Height (cm mean ± SD) | NR |
| Weight (kg mean ± SD) | NR |

Waruingi 2019 – A randomised controlled trial of the effect of laryngeal mask airway manometry on postoperative sore throat in spontaneously breathing adult patients presenting for surgery at a university teaching hospital

| **Characteristic** | **Value** |
| --- | --- |
| Country | Kenya |
| Total sample size (N) | 100 |
| Female (N) | 37 |
| Smoking status (N) | NR |
| Length of follow-up (h) | 12 |
| Airway control | LMA |
| Cuff pressure (cm H₂O ETT tube) | NR |
| Cuff pressure (cm H₂O SAD) | as per trial arm |
| Intubation attempts allowed | ≤ 2 |
| Age (mean ± SD, yr) | 34.00 ± 3.17 |
| BMI (mean ± SD) | NR |
| Duration of anaesthetic (min ± SD) | NR |
| Height (cm mean ± SD) | NR |
| Weight (kg mean ± SD) | 71.50 ± 3.08 |

Wong 2013 – New supraglottic airway with built-in pressure indicator decreases postoperative pharyngolaryngeal symptoms: a randomized controlled trial

| **Characteristic** | **Value** |
| --- | --- |
| Country | Canada |
| Total sample size (N) | 175 |
| Female (N) | 80 |
| Smoking status (N) | NR |
| Length of follow-up (h) | 24 |
| Airway control | LMA, AES Ultra CPV |
| Cuff pressure (cm H₂O ETT tube) | NR |
| Cuff pressure (cm H₂O SAD) | as per trial arm |
| Intubation attempts allowed | ≤ 2 |
| Age (mean ± SD, yr) | 50.50 ± 15.03 |
| BMI (mean ± SD) | 28.50 ± 5.53 |
| Duration of anaesthetic (min ± SD) | 47.50 ± 10.98 |
| Height (cm mean ± SD) | NR |
| Weight (kg mean ± SD) | NR |

## Paediatric

Amucheazi 2019 – Intracuff alkalinized lidocaine and the incidence of cough and postoperative sore throat after anesthesia in children: a randomized clinical trial

| **Characteristic** | **Value** |
| --- | --- |
| Country | Nigeria |
| Total sample size (N) | 100 |
| Female (N) | 51 |
| Smoking status (N) | NR |
| Length of follow-up (h) | 24 |
| Airway control | ETT |
| Cuff pressure (cm H₂O) | 27-30 |
| Intubation attempts allowed | ≤ 1 |
| Age (mean ± SD, yr) | 7.22 ± 3.52 |
| BMI (mean ± SD) | NR |
| Duration of anaesthetic (min ± SD) | 149.50 ± 27.16 |
| Height (cm mean ± SD) | NR |
| Weight (kg mean ± SD) | 26.50 ± 7.76 |

Kaur 2024 – Intracuff alkalinized 2 % lignocaine versus air for endotracheal tube induced postoperative sore throat and other laryngotracheal morbidities: a randomized, controlled trial

| **Characteristic** | **Value** |
| --- | --- |
| Country | India |
| Total sample size (N) | 64 |
| Female (N) | 27 |
| Smoking status (N) | NR |
| Length of follow-up (h) | 24 |
| Airway control | ETT |
| Cuff pressure (cm H₂O) | ≤ 20 |
| Intubation attempts allowed | ≤ 2 |
| Age (mean ± SD, yr) | 7.31 ± 2.57 |
| BMI (mean ± SD) | 11.41 ± 9.64 |
| Duration of anaesthetic (min ± SD) | 152.81 ± 35.56 |
| Height (cm mean ± SD) | NR |
| Weight (kg mean ± SD) | 21.84 ± 7.63 |

Moham Mokhtar 2013 – Postoperative sore throat in children: comparison between ProSeal™ LMA and Classic™ LMA

| **Characteristic** | **Value** |
| --- | --- |
| Country | Malaysia |
| Total sample size (N) | 200 |
| Female (N) | 103 |
| Smoking status (N) | NR |
| Length of follow-up (h) | 6 |
| Airway control | LMA (ProSeal vs Classic) |
| Cuff pressure (cm H₂O) | NR |
| SAD cuff pressure (cm H₂O) | ≤ 60 |
| Intubation attempts allowed | ≤ 3 |
| Age (mean ± SD, yr) | 9.15 ± 1.99 |
| BMI (mean ± SD) | NR |
| Duration of anaesthetic (min ± SD) | 40.45 ± 17.23 |
| Height (cm mean ± SD) | NR |
| Weight (kg mean ± SD) | 31.90 ± 8.18 |

Soares 2017 – The effects of tracheal tube cuffs filled with air, saline or alkalinised lidocaine on haemodynamic changes and laryngotracheal morbidity in children: a randomised, controlled trial

| **Characteristic** | **Value** |
| --- | --- |
| Country | Brazil |
| Total sample size (N) | 164 |
| Female (N) | 69 |
| Smoking status (N) | NR |
| Length of follow-up (h) | 8 |
| Airway control | ETT |
| Cuff pressure (cm H₂O) | ≤ 20 |
| Intubation attempts allowed | ≤ 2 |
| Age (mean ± SD, yr) | 7.63 ± 2.30 |
| BMI (mean ± SD) | NR |
| Duration of anaesthetic (min ± SD) | 129.75 ± 60.22 |
| Height (cm mean ± SD) | 121.69 ± 17.58 |
| Weight (kg mean ± SD) | 32.75 ± 13.70 |

Yhim 2020 – Effects of benzydamine hydrochloride on postoperative sore throat after extubation in children: a randomized controlled trial

| **Characteristic** | **Value** |
| --- | --- |
| Country | Korea (Republic of) |
| Total sample size (N) | 144 |
| Female (N) | 82 |
| Smoking status (N) | NR |
| Length of follow-up (h) | 0.5 |
| Airway control | ETT |
| Cuff pressure (cm H₂O) | 20 |
| Intubation attempts allowed | ≤ 2 |
| Age (mean ± SD, yr) | 9.40 ± 1.99 |
| BMI (mean ± SD) | NR |
| Duration of anaesthetic (min ± SD) | 153.05 ± 66.90 |
| Height (cm mean ± SD) | 137.85 ± 14.27 |
| Weight (kg mean ± SD) | 36.85 ± 12.75 |

h – hours, SD – standard deviation
